# MAT2B regulates the protein level of MAT2A to preserve RNA N6-methyladenosine

**DOI:** 10.1038/s41419-024-07093-8

**Published:** 2024-10-01

**Authors:** Xinyi Wan, Weiwu Zeng, Haonan Fan, Chenliang Wang, Shixun Han, Zhongxing Sun, Mei Tang, Juejia Shao, Yu Liu, Yuan Fang, Junqi Jia, Yin Tang, Yanjun Zhang, Bin Zhao, Dong Fang

**Affiliations:** 1 The Second Affiliated Hospital of Zhejiang University School of Medicine, Life Sciences Institute, Zhejiang University, Hangzhou, China; 2 Life Sciences Institute, Zhejiang University, Hangzhou, China; 3 Department of Medical Oncology, Key Laboratory of Cancer Prevention and Intervention, Ministry of Education, The Second Affiliated Hospital, Zhejiang University School of Medicine, Hangzhou, Zhejiang China; 4 Key Laboratory of Cancer Prevention and Intervention, China National Ministry of Education, Hangzhou, China

**Keywords:** Epigenetics, Mechanisms of disease

## Abstract

MAT2B works together with MAT2A to synthesize S-Adenosyl methionine (SAM) as the primary methyl donor. MAT2B, despite lacking catalytic activity, exerts regulatory control over the enzymatic activity of MAT2A. In addition to the enzymatic activity regulation, we find that, in an NADP^+^-dependent manner, MAT2B binds and stabilizes MAT2A. Disruption of the cellular NADP^+^ remodels the protein level of MAT2A. The pentose phosphatase pathway regulates the level of MAT2A protein through the interaction of NADP^+^ with MAT2B. Additionally, MAT2B-MAT2A interaction regulates the mRNA m6A modification and stability. In liver tumors, the *Mat2a* mRNA level is elevated but the protein level is decreased by the restricted NADP^+^. Blocking the interaction between MAT2B and MAT2A by the keto diet can suppress liver tumor growth. These findings reveal that MAT2B is essential for regulating the protein levels of MAT2A and connecting SAM synthesis to mRNA m6A.

## Introduction

Epigenetics is the process of modifying gene expression in a heritable manner without altering the DNA sequence. Methylations give rise to modified histones, DNA, and RNA, all of which play significant roles in the epigenetic regulation of gene expression [1–3]. Methylation of mRNA to form the most abundant internal modification, N6-methyladenosine (m6A), has become a widespread regulatory mechanism that controls gene expression in diverse physiological processes [4–6].

Although different methyltransferases catalyze the methylation of various substrates, such as nucleic acids, proteins, lipids, and secondary metabolites, they all use S-Adenosyl methionine (SAM) as the primary methyl donor [7, 8]. Methionine adenosyltransferase (MAT), also known as *S*-adenosine methionine synthase, is an essential enzyme that catalyzes the synthesis of SAM from ATP and methionine [9–11]. Mammals possess two distinct genes, namely *Mat1a* and *Mat2a*, which encode two catalytic subunits that share a homologous nature [9]. *Mat2a* is mainly expressed in various tissues. In contrast, *Mat1a* is primarily expressed in the liver, maintaining the differentiated states of bile duct epithelial cells and hepatocytes [12, 13]. In addition, only *Mat2a* is expressed in the fetal liver. During liver development, the expression of MAT genes shifts from *Mat2a* to *Mat1a*. However, in cases of liver malignant transformation, the pattern is reversed, with *Mat2a* becoming the predominantly expressed gene [10, 14, 15].

MAT2B, which is documented as the regulatory partner of MAT2A, is another MAT protein without catalytic activity [13, 14, 16]. MAT2B is known to regulate the catalytic activity of MAT2A by altering its kinetic properties [17], which increases its binding to L-methionine [10, 18, 19]. Although previous studies have indicated that MAT2B can regulate MAT2A activity by altering its affinity for substrates and sensitivity to product inhibition, there are also studies demonstrating that the binding of MAT2B to MAT2A does not significantly affect MAT2A’s catalytic activity or physiologically relevant inhibition of SAM [20]. Therefore, the exact mechanism by which MAT2B regulates the activity of MAT2A remains unknown.

Here, we find MAT2B binds and stabilizes MAT2A in an NADP^+^-dependent manner by repressing its acetylation. The dysregulation of their interaction by NADP^+^ restriction has a significant impact on mRNA m6A modification and stability. In liver tumors, although the *Mat2a* mRNA level is elevated, the decrease of NADP^+^ represses the stabilizing effect of MAT2B on MAT2A, resulting in decreased MAT2A protein levels. The suppression of liver tumor growth can be achieved through the blocking of the interaction between MAT2B and MAT2A by the keto diet. These findings suggest that MAT2B plays a crucial role in regulating the protein levels of MAT2A and the integration of SAM synthesis with mRNA m6A.

## Results

### MAT2B regulates the protein stability of MAT2A

**Fig. 1:**
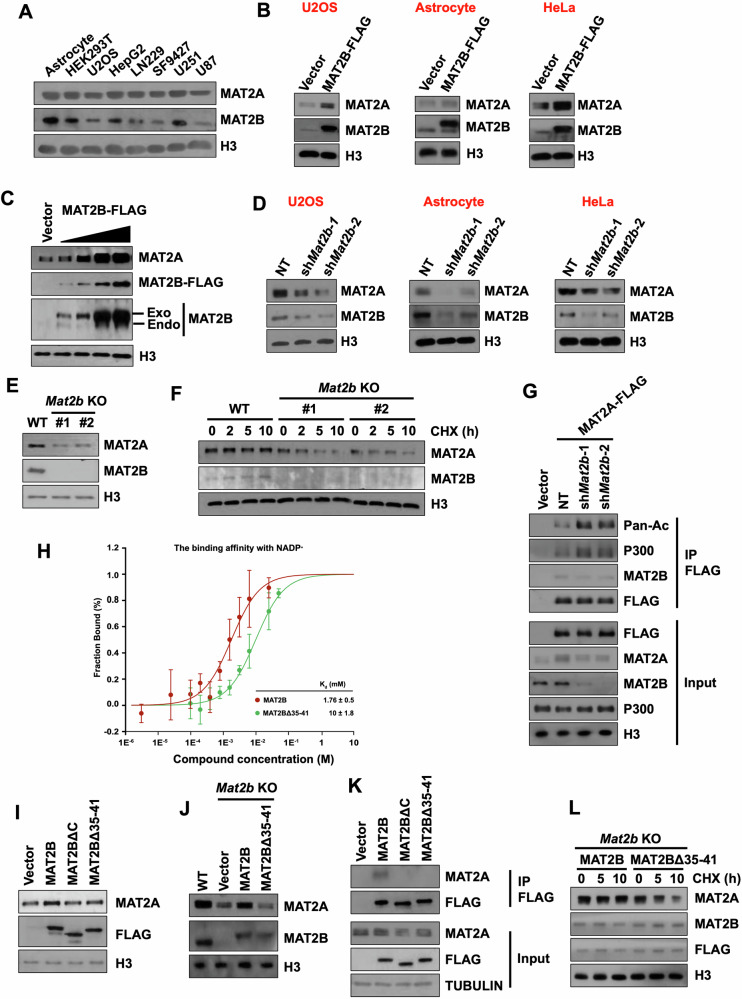
MAT2B regulates the protein level of MAT2A. **A** Western blotting results showing the protein levels of MAT2A and MAT2B in different cell lines. H3 was used as the loading control. The assay was repeated twice with similar results. **B** Western blotting results showing MAT2A and MAT2B protein levels after MAT2B was over-expressed in U2OS, Astrocyte, and HeLa cells. An empty vector was used as the negative control. Each assay was repeated three times with similar results. **C** The protein levels of MAT2A gradually increased after different amounts of MAT2B were over-expressed. An empty vector was used as the negative control. The U2OS cells were used and were infected with varying amounts of Lentivirus expressing MAT2B to create a protein gradient overexpression. The assay was repeated twice with similar results. **D** Western blotting results showing MAT2A and MAT2B protein levels after *Mat2b* was knocked down in U2OS, Astrocyte, and HeLa cells. *Mat2b* was knocked down by two independent shRNAs. NT, non-target control. Each assay was repeated three times with similar results. **E** Western blotting results showing MAT2A and MAT2B protein levels after *Mat2b* was knocked out in U2OS cells. *Mat2b* was knocked out by two independent sgRNAs. The assay was repeated at least three times with similar results. **F** MAT2A degraded slower in the WT U2OS cell line than in two *Mat2b* KO U2OS cell lines. Cells were collected at intervals of 0, 2, 5, and 10 h after 25 μg/mL cycloheximide treatment. Western blotting was conducted using the indicated antibodies. The assay was repeated three times with similar results. **G** Depletion of MAT2B increased the acetylation of MAT2A and the interaction between MAT2A and P300. MAT2A was purified by FLAG IP in HEK293T cells overexpressing FLAG-tagged MAT2A. HEK293T cells transfected with empty vectors were used as negative controls. Proteins from input and IP samples were analyzed by Western blotting using the indicated antibodies. The assay was repeated twice with similar results. **H** The K_d_ value between MAT2B and NADP^+^ decreased when the NADP^+^-binding domain was truncated. Microscale Thermophoresis (MST) assay was conducted with purified MAT2B. Data were mean ± SD (N = 3 independent experiments). **I** Western blotting results showing MAT2A protein levels after over-expression of WT and mutant MAT2B. The empty vector was used as the negative control. WT MAT2B, MAT2BΔC, and MAT2BΔ35-41 were transfected into WT U2OS cells, respectively. The assay was repeated three times with similar results. **J** Re-expression of MAT2B Δ35-41 did not restore the protein level of MAT2A in *Mat2b* KO cells. Western blotting results showed the protein levels of MAT2A in indicated cell lines. The assay was repeated at least three times with similar results. **K** The truncation of MAT2A or NADP^+^-binding domain disrupted the binding between MAT2A and MAT2B. WT and mutant MAT2B were purified by FLAG IP in HEK293T cells overexpressing FLAG-tagged proteins. Proteins from input and IP samples were analyzed by Western blotting using the indicated antibodies. The assay was repeated three times with similar results. **L** MAT2A degraded faster in MAT2BΔ35-41 re-expressed cells compared to WT MAT2B re-expressed U2OS cells. Cells were collected at intervals of 0, 5, and 10 h after 25 μg/ml cycloheximide treatment. Western blotting was conducted using the indicated antibodies. The assay was repeated twice with similar results.

To analyze how MAT2B and MAT2A are orchestrated in cells, we detected their protein levels in different cell lines by Western Blotting. The result showed that the protein levels of MAT2A and MAT2B were correlatively higher or lower among these analyzed cell lines, indicating that MAT2B and MAT2A may regulate each other (Fig. 1A). In addition, we investigated the relationship between the expression levels of *Mat2a* and *Mat2b* using RNA-Seq data from all available human tissues, cell lines, and primary cells in the ENCODE project. The result revealed a strong correlation between *Mat2a* and *Mat2b* (Supplementary Fig. 1A). We then over-expressed MAT2B in three different cell lines and found that the protein levels of MAT2A were increased in all three cell lines (Fig. 1B). When different amounts of MAT2B were over-expressed in cells, the protein levels of MAT2A were correspondingly increased with the elevated MAT2B (Fig. 1C). In addition, we knocked down *Mat2b* in five cell lines and found that the protein levels of MAT2A were decreased in all analyzed cell lines (Fig. 1D and Supplementary Fig. 1B). However, when we over-expressed or depleted MAT2A, the protein levels of MAT2B were not affected (Supplementary Fig. 1C, D).

To further analyze how MAT2A and MAT2B interplayed with each other, we utilized the CRISPR/Cas9 system to knock out (KO) *Mat2b* in U2OS cells. Two KO clones were established from two independent sgRNAs bearing 1 and 2 base-pair insertions, respectively (Supplementary Fig. 1E). Consistent with our previous observations, the protein levels of MAT2A decreased when *Mat2b* was knocked out (Fig. 1E). Because the alternations of MAT2A may result from changes of mRNA or protein degradation, we then analyzed how *Mat2b* KO affected the expression and protein stability of MAT2A. The results showed that the protein stability but not mRNA levels of MAT2A were decreased in *Mat2b* KO cells, indicating that MAT2B directly regulated the protein level of MAT2A (Fig. 1F and Supplementary Fig. 1F). The levels of MAT2A and MAT2B did not decrease in WT cells, likely because these proteins are very stable and were not degraded. Moreover, the over-expression or depletion of MAT2B didn’t affect the mRNA levels of *Mat2a* (Supplementary Fig. 1G, H). Previous studies showed that P300 acetylated MAT2A to promote its ubiquitylation and subsequently proteasomal degradation [21]. We then depleted MAT2B and analyzed the acetylated level of ectopically expressed MAT2A by the pan acetyl-lysine antibody. Indeed, the acetylation of MAT2A and co-purified P300 with MAT2A were increased in MAT2B depleted cells (Fig. 1G). Additionally, we discovered that knocking down P300 increased the binding between MAT2A and MAT2B (Supplementary Fig. 1I). These findings suggest that MAT2B and P300 competitively bind with MAT2A.

As previously reported [22, 23], MAT2A can be immunoprecipitated with MAT2B (Supplementary Fig. 1J). Please note that to detect the binding between MAT2A and MAT2B, we expressed MAT2B at a low level to avoid increasing the protein level of MAT2A. We then tested the idea that MAT2B regulated the protein level of MAT2A through its interaction with MAT2A. We over-expressed wild-type (WT) MAT2B and MAT2B with a deletion of C-terminal domain (MAT2BΔC) [23], which was reported as a potential MAT2A binding domain, and analyzed the protein levels of MAT2A, respectively. In addition, because MAT2B is an NADP^+^ binding protein [23, 24] (Fig. 1H and Supplementary Fig. 1K), we also analyzed the function of MAT2B with a truncation of NADP^+^-binding GXXGXXG domain (MAT2BΔ35-41). The over-expression of MAT2BΔC showed no effect on the protein level of MAT2A (Fig. 1I). Interestingly, MAT2B could not increase the protein level of MAT2A when the NADP^+^-binding domain was truncated. To further confirm this, we re-expressed MAT2B and MAT2BΔ35-41 in *Mat2b* KO cells. Only WT MAT2B but not MAT2BΔ35-41 mutant could rescue the decreased MAT2A in *Mat2b* KO cells (Fig. 1J). Consistent with these observations, the binding between MAT2A and MAT2B was largely abolished when the NADP^+^-binding domain or the C-terminal domain was truncated (Fig. 1K). Moreover, the protein stability of MAT2A was higher in WT MAT2B re-expressed cells than in MAT2BΔ35-41 re-expressed cells, leaving the mRNA level of *Mat2a* unchanged (Fig. 1L and Supplementary Fig. 1L). We also analyze how NADP(H) and its relative NAD(H) were affected by the alternation of MAT2A and MAT2B. The results showed that the contents of NADP(H) and NAD(H) remained unchanged when *Mat2a* or *Mat2b* were up- and down-regulated (Supplementary Fig. 1M–Q). Together, these results suggest that the protein level of MAT2A is regulated by MAT2B, which is facilitated by MAT2B’s NADP^+^-binding domain.

### The regulation of MAT2A by MAT2B is highly reliant on NADP^+^

**Fig. 2:**
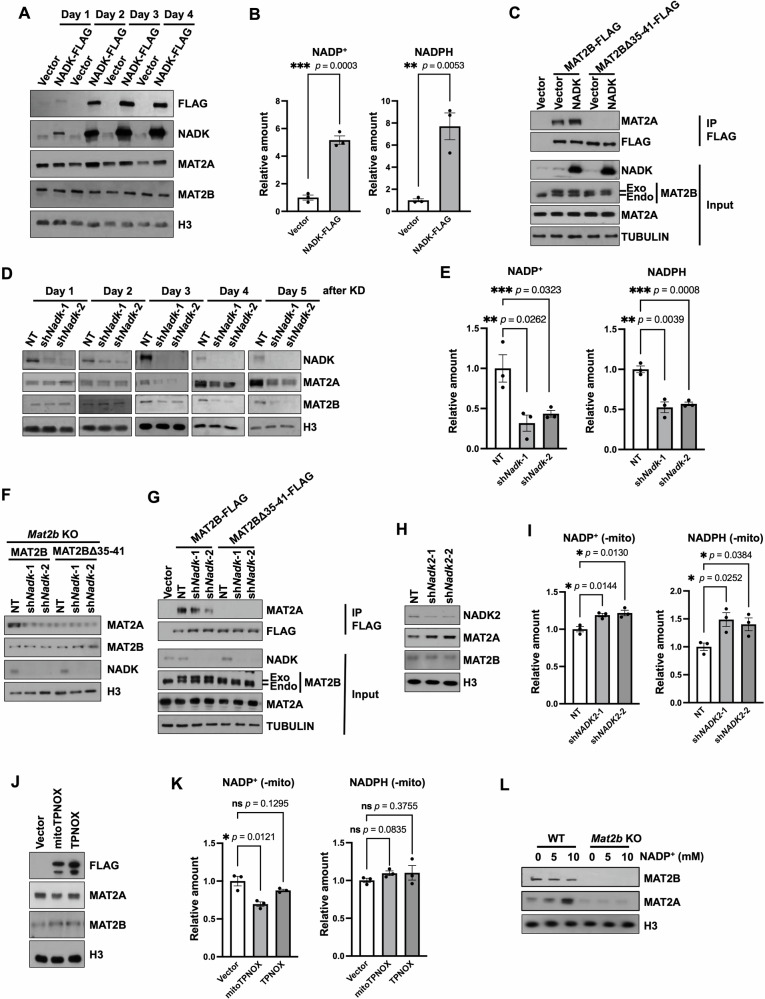
MAT2B regulates MAT2A in an NADP^+^-dependent manner. **A** MAT2A increased two days after NADK over-expression. The U2OS cells with or without NADK overexpression were collected daily for 4 days after virus infection. The assay was repeated twice with similar results. **B** The total level of NADP(H) increased two days after NADK over-expression. The contents of individual compounds in empty-vector transfected U2OS cells, as determined by mass spectrometry, were normalized as 1. The data were represented by the mean ± SEM (N = 3 independent replicates). The *p* value was determined by two-sided unpaired t-test. **C** NADK overexpression increased the interaction between MAT2B and MAT2A. MAT2B was purified by FLAG IP. HEK293T cells transfected with empty vectors were used as negative controls. Proteins from input and IP samples were analyzed by Western blotting using the indicated antibodies. To avoid the changes of MAT2A, cells were collected one day after NADK over-expression. The assay was repeated twice with similar results. **D** Depletion of NADK reduced the protein level of MAT2A. U2OS cells with or without NADK depletion were collected daily for 5 days after virus infection. The assay was repeated twice with similar results. **E** The total level of NADP(H) decreased after NADK depletion. The contents of individual compounds in NT shRNA treated U2OS cells, as determined by mass spectrometry, were normalized as 1. The data were represented by the mean ± SEM (N = 3 independent replicates). The *p* value was determined by two-sided unpaired t-test. **F** Western blotting results showing the protein levels of MAT2A in WT MAT2B and MAT2BΔ35-41 rescued U2OS cell lines with NADK depletion. The assay was repeated three times with similar results. **G** Depletion of NADK reduced the interaction between MAT2B and MAT2A. To avoid the changes of MAT2A and MAT2B, cells were collected one day after the depletion of NADK. Proteins from input and IP samples were analyzed by Western blotting using the indicated antibodies. The assay was repeated twice with similar results. **H** NADK2 depletion increased the protein level of MAT2A. Two independent shRNAs were used to deplete NADK2 in U2OS cells. The assay was repeated three times with similar results. **I** The contents of NADP(H) outside mitochondria increased after *Nadk2* was knocked down. The contents of individual compounds in NT shRNA treated U2OS cells, as determined by mass spectrometry, were normalized as 1. The data were represented by the mean ± SEM (N = 3 independent replicates). The *p* value was determined by two-sided unpaired t-test. **J** The overexpression of mitoTPNOX decreased the protein level of MAT2A in U2OS cells. The assay was repeated four times with similar results. **K** Overexpression of mitoTPNOX decreased the contents of NADP^+^ outside mitochondria. The contents of individual compounds in empty-vector transfected U2OS cells, as determined by mass spectrometry, were normalized as 1. The data were represented by the mean ± SEM (N = 3 independent replicates). The *p* value was determined by two-sided unpaired t-test. **L** NADP^+^ incubation increased MAT2A in WT U2OS cells but not in *Mat2b* KO U2OS cells. 0, 5, and 10 mM NADP^+^ were transfected into cells for 48 h and cell extracts were analyzed by Western blotting using the indicated antibodies. The assay was repeated twice with similar results.

To further examine the in vivo function of NADP^+^ on protein levels of MAT2A, we over-expressed NADK, the kinase responsible for the phosphorylation of NAD^+^ to generate NADP^+^ in the cytoplasm [25, 26], and then examined the protein level of MAT2A. After two days of NADK over-expression, the protein levels of MAT2A were increased (Fig. 2A). The content of NADP(H) increased one day after NADK was over-expressed (Fig. 2B). It’s possible that the MAT2A is stabilized and showed elevated levels at a subsequent time. Consistently, over-expression of NADK in astrocyte cells also increased the protein level of MAT2A (Supplementary Fig. 2A). Since we found the binding between MAT2A and MAT2B was important for the stabilization of MAT2A, we further analyzed the interaction between MAT2A and MAT2B when NADK was over-expressed. To minimize the changes in protein levels of MAT2A, we expressed MAT2B at low levels and conducted MAT2B immunoprecipitation (IP) one day after NADK overexpression. While MAT2A was not immunoprecipitated with MAT2BΔ35-41, the over-expression of NADK increased the co-purified MAT2A with MAT2B (Fig. 2C).

We further knocked down *Nadk* and found the protein level of MAT2A decreased, leaving the mRNA level unchanged (Supplementary Fig. 2B, C). However, the protein level of MAT2B also decreased, likely being caused by the secondary effects of the shortage of NADP^+^ (Supplementary Fig. 2C). We then performed a time course analysis after *Nadk* knockdown to determine whether MAT2A decreased before MAT2B. After two days of NADK depletion, we observed a slight decrease in MAT2A, leaving MAT2B unchanged. The MAT2B and MAT2A both diminished with a prolonged time of *Nadk* knockdown (Fig. 2D). Accordingly, the amount of NADP(H) reduced after two days of NADK depletion (Fig. 2E). In addition, we knocked down *Nadk* in HEK293T and Astrocyte cells. Like what was observed in U2OS cells, the depletion of NADK decreased MAT2A after two days and diminished MAT2B after three days (Supplementary Fig. 2D, E). To further reduce the impact of NADK depletion on protein levels of MAT2B, we knocked down *Nadk* in WT MAT2B and MAT2BΔ35-41 re-expressed cells in which endogenous MAT2B was knocked out. Since the expression of MAT2B was controlled by an exogenous promoter, the protein levels of WT MAT2B and MAT2BΔ35-41 were not altered when NADK was depleted. The protein levels of MAT2A decreased in WT MAT2B re-expressed cells but not in MAT2BΔ35-41 rescued cells upon NADK depletion (Fig. 2F). Moreover, we analyzed the interaction between MAT2A and MAT2B when NADK was depleted. To minimize the changes of MAT2A, we expressed MAT2B at a low level and conducted MAT2B immunoprecipitation one day after *Nadk* depletion. The depletion of NADK largely abolished the co-purified MAT2A with MAT2B (Fig. 2G), further supporting the idea that MAT2B regulates the protein level of MAT2A depending on NADP^+^.

NADK2, which is the mitochondrial NAD(H) kinase, mediates the mitochondrial NADP(H) generation [27–29]. When we depleted NADK2 to decrease the NADP(H) in mitochondria, we detected an increase of MAT2A which seems to contradict the reduction of mitochondrial NADP(H) generation (Fig. 2H). Previous reports showed that the contents of NADP^+^ in the whole cell remained unchanged when NADK2 was depleted [28]. We speculated that the amount of NADP^+^ outside mitochondria was increased. We then fractionated the NADK2 depleted cells and found that the NADP(H) level outside mitochondria was indeed increased, which was correlated with the elevated MAT2A (Fig. 2I). Moreover, we expressed TPNOX, which is an NADPH oxidase, and its mitochondrial signal-tagged form (mitoTPNOX) [30, 31]. Only mitoTPNOX, which was located in mitochondria to increase the total level of NADP^+^ in cells, decreased the amount of MAT2A (Fig. 2J and Supplementary Fig. 2F). More importantly, only the contents of NADP^+^, but not NADPH, outside mitochondria were decreased in mitoTPNOX-expressed cells, which was consistent with the decreased MAT2A (Fig. 2K).

To further verify the above hypothesis that NADP^+^ is critical for the regulation of MAT2A, we planned to alter the content of NAD(H), a relative of NADP(H). Aminocarboxymuconate Semialdehyde Decarboxylase (ACMSD) catalyzes the generation of key precursors for NAD(H) [32]. We depleted or over-expressed ACMSD in cells and found that the protein levels of MAT2A were unaffected (Supplementary Fig. 2G–I). Nicotinamide phosphoribosyltransferase (NAMPT) is the rate-limiting enzyme in the NAD^+^ salvage pathway [33]. We treated cells with NAMPT inhibitors (FK866) or activators (P7C3) to reduce or increase NAD(H), respectively (Supplementary Fig. 2J). The protein levels of MAT2A were unchanged by the incubation of either small molecule (Supplementary Fig. 2K, L), indicating the contents of NAD(H) had little effect on the protein level of MAT2A. We conducted cell transfection experiments using varying amounts of NADP^+^ in both WT and *Mat2b* KO cells. The results showed that MAT2A increased in WT cells after NADP^+^ treatment, but not in *Mat2b* KO cells (Fig. 2L). Together, these results indicate that MAT2B regulates the protein level of MAT2A in an NADP^+^-dependent manner.

### The pentose phosphate pathway regulates the level of MAT2A protein

**Fig. 3:**
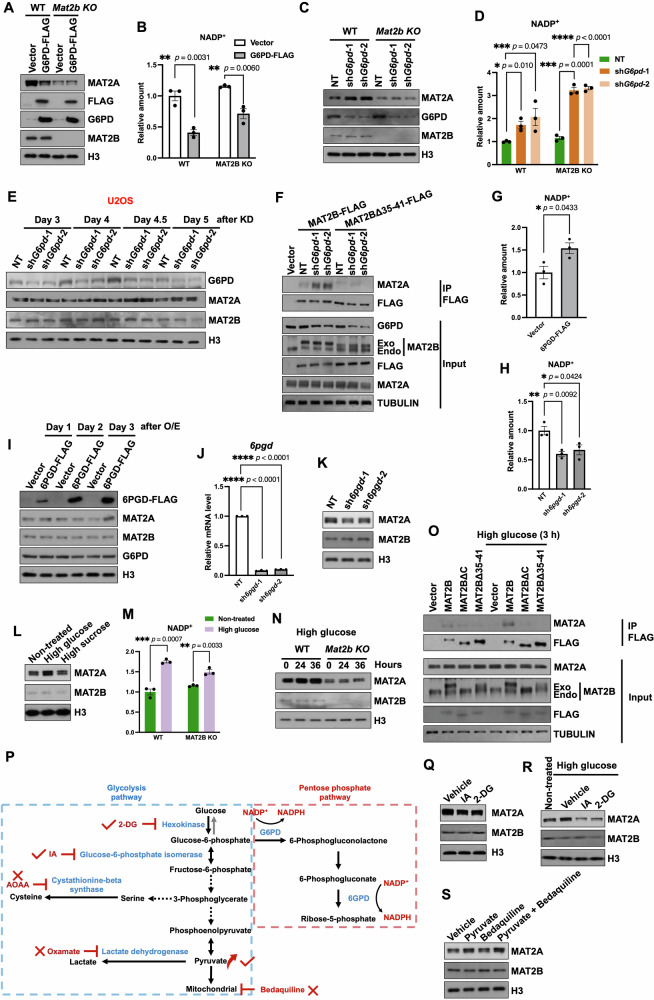
The regulation of MAT2A by PPP depends on NADP^+^. **A** G6PD overexpression decreased MAT2A in WT U2OS cells but not in *Mat2b* KO U2OS cells. The assay was repeated twice with similar results. **B** Mass spectrometry results showing that the levels of NADP^+^ decreased when G6PD was over-expressed. The contents of individual compounds in empty-vector transfected WT U2OS cells were normalized as 1. The data were represented by the mean ± SEM (N = 3 independent replicates). The *p* value was determined by two-sided unpaired t-test. **C** Depletion of G6PD increased the level of MAT2A in WT but not in *Mat2b* KO U2OS cells. The assay was repeated twice with similar results. **D** Mass spectrometry results showing that the levels of NADP^+^ increased when G6PD was depleted. NT, non-target control. The contents of individual compounds in NT shRNA treated WT U2OS cells were normalized as 1. The data were represented by the mean ± SEM (N = 3 independent replicates). The *p* value was determined by two-sided unpaired t-test. **E** Western blotting results showing that, after *G6pd* knocked down, MAT2A increased after 4.5 days. U2OS cells were collected for Western blotting at indicated time points after *G6pd* was knocked down. The assay was repeated twice with similar results. **F** Depletion of G6PD increased the interaction between MAT2B and MAT2A. To avoid the changes of MAT2A, cells were collected 4 days after G6PD depletion. The assay was repeated twice with similar results. Mass spectrometry results showing that the levels of NADP^+^ increased and decreased with 6PGD overexpression (**G**) and depletion (**H**), respectively. The contents of individual compounds in empty vector (**G**) or NT shRNA treated (**H**) U2OS cells were normalized as 1, respectively. The data were represented by the mean ± SEM (N = 3 independent replicates). The *p* value was determined by two-sided unpaired t-test. **I** 6PGD overexpression increased the protein level of MAT2A in U2OS. The cells were collected daily for Western blotting following 6PGD overexpression. The assay was repeated twice with similar results. **J** *6pgd* was knocked down in U2OS cells as detected by RT-qPCR. *6pgd* was knocked down by 2 independent shRNAs. NT, non-target control. Gene expressions were normalized to *β-actin* and the expression levels in NT cells were further normalized as 1. The data were represented by the mean ± SEM (N = 3 independent replicates). The *p* value was determined by two-sided paired t-test. **K** Western blotting results showing that, with 6PGD depletion, the protein level of MAT2A decreased in U2OS cells. The assay was repeated twice with similar results. **L** The protein level of MAT2A was elevated by high glucose treatment in U2OS cells. High glucose treatment, 40 g/L glucose incubation for 36 h. High sucrose treatment, 20 g/L sucrose (same osmotic pressure as 40 g/L glucose) incubation for 36 h. The assay was repeated three times with similar results. **M** High glucose treatment increased MAT2A in WT but not *Mat2b* KO U2OS cells. Samples were collected at the indicated time points after high glucose (40 g/L) treatment. The assay was repeated twice with similar results. **N** Mass spectrometry results showing that the levels of NADP(H) increased with high glucose incubation. High glucose treatment, 40 g/L glucose incubation for 36 h. The contents of individual compounds in non-treated WT U2OS cells were normalized as 1. The data were represented by the mean ± SEM (N = 3 independent replicates). The *p* value was determined by two-sided unpaired t-test. **O** The interaction between MAT2A and MAT2B was elevated by high glucose treatment. To avoid the changes of MAT2A, cells were collected 3 h after high glucose treatment. The assay was repeated twice with similar results. **P** The illustration of pathways of glycolysis and Pentose phosphate pathway (PPP). “**√**” represented that the compounds changed MAT2A protein levels and “**×**” represented that the compounds showed no obvious alternations in MAT2A protein levels. **Q** IA (10 μM for 24 h) and 2-DG (10 mM for 24 h) treatments decreased MAT2A in U2OS cells. Cells treated with 0.1% DMSO were used for the vehicle control. The assay was repeated twice with similar results. **R** Co-incubation with IA (10 μM for 36 h) or 2-DG (10 mM for 36 h) inhibited the increase of MAT2A induced by high glucose (40 g/L for 36 h) in U2OS cells. The assay was repeated twice with similar results. **S** Pyruvate (10 mM for 24 h) increased the level of MAT2A while the mitochondrial function inhibitor, Bedaquiline (10 μM for 24 h), showed no obvious effect on MAT2A levels in U2OS cells. Co-incubation with Bedaquiline did not inhibit the increase of MAT2A induced by pyruvate. Cells treated with 0.1% DMSO were used for the vehicle control. The assay was repeated twice with similar results.

In mammalian cells, NADP^+^ is mainly consumed through the pentose phosphate pathway (PPP) [34]. To investigate the function of PPP in regulating MAT2A, we over-expressed G6PD, the first step enzyme of PPP [35], resulting in decreased MAT2A protein levels (Supplementary Fig. 3A, B). Moreover, we over-expressed G6PD in WT and *Mat2b* KO cells. MAT2A levels decreased only in WT cells but not in *Mat2b* KO cells. (Fig. 3A). Accordingly, the contents of NADP^+^ were diminished when G6PD was elevated (Fig. 3B). After knocking down *G6pd*, we observed an increase in protein levels of MAT2A (Supplementary Fig. 3C–F). Consistent with the over-expression results, depletion of G6PD increased NADP^+^ levels and elevated protein levels of MAT2A only in WT cells, not in *Mat2b* KO cells, indicating a dependence on MAT2B (Fig. 3C, D). To investigate the role of G6PD in regulating MAT2A, we sought to validate the impact of G6PD depletion on the interaction between MAT2A and MAT2B. First, we analyzed the time required for the shRNA against *G6pd* to increase the levels of MAT2A protein. This step is important because altered MAT2A levels can interfere with interactions between MAT2A and MAT2B. Five days after the shRNA transduction, an increase in MAT2A was detected in both HEK293T and U2OS cells (Fig. 3E and Supplementary Fig. 3G). Then, we chose to perform the IP analysis four days after the shRNA transduction. The results showed that the knockdown of *G6pd* increased the co-purified MAT2A with MAT2B and showed little effect on the binding between MAT2A and MAT2BΔ35-41 (Fig. 3F). We also over-expressed and depleted 6PGD, which is the downstream regulator of PPP [36], to detect the changes of MAT2A. In contrast to G6PD, the excessive production and shortage of 6PGD resulted in increased and decreased levels of NADP^+^, respectively (Fig. 3G, H). It’s possible that a feedback system of PPP was activated when 6PGD was dysfunctional. Nevertheless, MAT2A changed accordingly with the NADP^+^ (Fig. 3I–K).

Generally, there are two other direct routes for NADP^+^ consumption: malic enzymes (MEs) in glutaminolysis and methylenetetrahydrofolate dehydrogenase (MTHFD) in folate metabolism [37]. We depleted ME1, ME2, or MTHFD1 in U2OS and astrocyte cells, respectively. The total amounts of NADP^+^ and MAT2A remained unaltered (Supplementary Fig. 3H–O), suggesting that glutaminolysis and folate metabolism played less important roles than PPP in the regulation of NADP^+^ in these cells.

The PPP is a metabolic pathway parallel to glycolysis [38]. We then asked how the glycolysis pathway would affect MAT2A. We treated the cells with high glucose and found that the protein levels of MAT2A were elevated (Fig. 3L and Supplementary Fig. 3P). As a control for the changes of osmotic pressure under high glucose conditions, the high sucrose treatment which had the same osmotic pressure didn’t change MAT2A levels. In addition, the high glucose treatment did not increase MAT2A levels in *Mat2b* KO cells, although the NADP^+^ levels were both elevated in two cells (Fig. 3M, N), suggesting that the regulation of high glucose on MAT2A is dependent on MAT2B. We then analyzed whether high glucose treatment increased interactions between MAT2A and MAT2B. Cells were treated with high glucose for a short time (3 h) to prevent an increase of MAT2A (Supplementary Fig. 3Q). MAT2A bound with more MAT2B proteins under high glucose treatment (Fig. 3O). Moreover, the high glucose treatment showed little effect on the binding between MAT2A and MAT2B mutants with truncations at the C-terminal domain or NADP^+^ binding domain.

To further evaluate glycolysis’ function in regulating MAT2A, we examined MAT2A protein levels in cells treated with glycolysis-targeting compounds [39] (Fig. 3P). The tested chemicals that hindered the glycolysis pathway altered the levels of MAT2A protein (Fig. 3Q, R, and Supplementary Fig. 3R, S). 2-DG (Hexokinase inhibitor) and IA (Glucose-6-phosphate isomerase inhibitor) lowered MAT2A levels by inhibiting glycolysis. Pyruvate, which may accelerate glycolysis [40], increased MAT2A protein levels (Fig. 3S and Supplementary Fig. 3T). We analyzed how NADP^+^ was affected by various reagents that could regulate cell metabolism. Consistent with the changes in MAT2A, IA and 2DG decreased NADP^+^ levels, while pyruvate increased NADP^+^ levels. The other reagents tested had little effect on NADP^+^ levels (Supplementary Fig. 3U). Together, these data suggest that the protein level of MAT2A is regulated by PPP and parallel glycolysis depending on NADP^+^.

### NADP^+^ regulates MAT2A to control the m6A modification and stability of mRNA

Depleting MAT2A reduced levels of histone methylation and mRNA m6A modification, but not DNA 5mC modification (Supplementary Fig. 4A–C). When *Mat2b* was knocked down or knocked out, the total levels of these analyzed methylations remained unchanged (Supplementary Fig. 4D–H). Moreover, the knockdown of *Nadk* had minimal impact on the levels of histone methylation, mRNA m6A modification, and DNA 5mC modification. (Supplementary Fig. 4I–K). Consistent with these observations, the amount of SAM was only affected by MAT2A depletion (Supplementary Fig. 4L–N). These results indicate that the methylation of these molecules is preserved when MAT2A is restricted by a lower level of MAT2B.

**Fig. 4:**
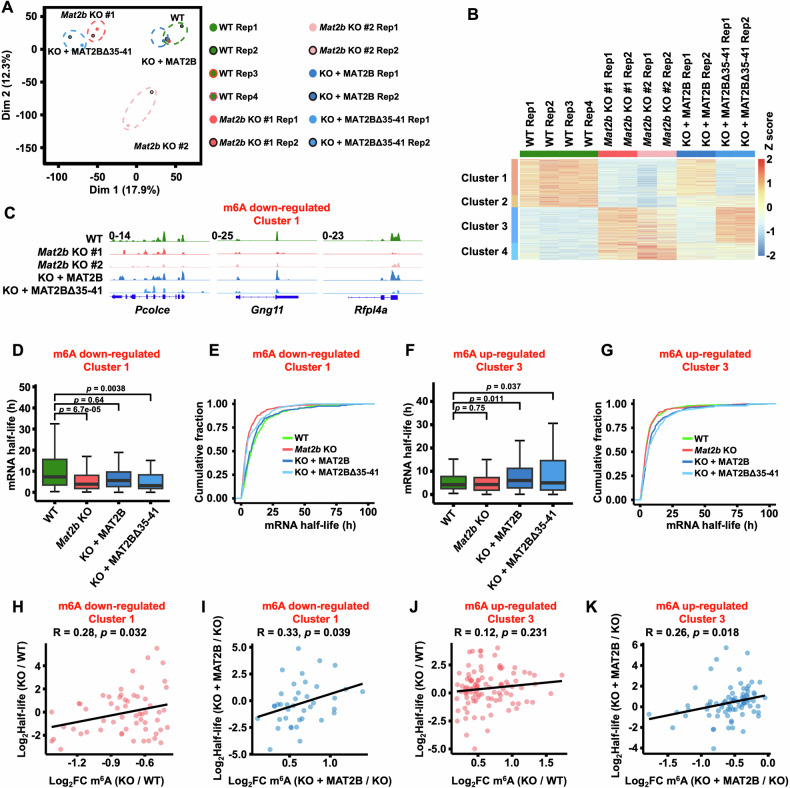
NADP^+^ regulates the m6A modification and stability of mRNA. **A** The principal component analysis (PCA) plot of the m6A MeRIP-Seq data from WT, *Mat2b* KO, WT MAT2B rescued, and MAT2BΔ35-41 rescued U2OS cells. At least two replicates of each cell line were presented. **B** Heatmap showing genes with different m6A levels in mRNA. These genes were divided into 4 clusters by K-Means clustering. **C** IGV tracks presenting the enrichments of m6A modification of genes in m6A down-regulated Cluster 1. Boxplots (**D**) and cumulative distributions (**E**) showing the half-life of genes in m6A down-regulated Cluster 1. The *p* values were determined by a two-tailed t-test. **F** Same as in (**D**), except genes in m6A up-regulated Cluster 3 were shown. **G** Same as in (**E**), except genes in m6A up-regulated Cluster 3 were shown. **H** Correlations between the changes of half-life and m6A modification in *Mat2b* KO U2OS cells compared with WT U2OS cells. Genes in m6A down-regulated Cluster 1 were shown. R, correlation coefficients that were assessed by Pearson product-moment correlation. The *p* values were calculated by a two-sided paired t-test. **I** Correlations between the changes of half-life and m6A modification in WT MAT2B re-expressed U2OS cells compared with *Mat2b* KO U2OS cells. Genes in m6A down-regulated Cluster 1 were shown. R, correlation coefficients that were assessed by Pearson product-moment correlation. The *p* values were calculated by a two-sided paired t-test. **J** Same as in (**H**), except genes in m6A up-regulated Cluster 3 were shown. **K** Same as in (**I**), except genes in m6A up-regulated Cluster 3 were shown.

We then speculated that m6A levels in individual mRNA were altered. We performed MeRIP-Seq to profile the mRNA m6A in WT, *Mat2b* KO, MAT2B re-expressed, and MAT2BΔ35-41 re-expressed cells, where the analyzed m6A methyltransferases and demethylases were unchanged (Supplementary Fig. 5A). The sequencing results from the two biological replicates showed a high correlation (Fig. 4A and Supplementary Table S1). The distribution of m6A in mRNA was similar across these cell lines (Supplementary Fig. 5B). Interestingly, we found that knocking out *Mat2b* resulted in both up- and down-regulation of m6A in mRNA (Fig. 4B). In addition, genes with altered m6A could be further divided into 4 clusters. When WT MAT2B was re-expressed, the m6A levels of genes in Cluster 1 and Cluster 3 were restored, but not when MAT2BΔ35-41 was re-expressed. This suggests that the interaction between MAT2B and MAT2A is responsible for regulating the m6A levels of these genes. This result was further confirmed by the Integrative Genomics Viewer (IGV) views (Fig. 4C and Supplementary Fig. 5C). As revealed by the Gene Ontology (GO) analysis, the m6A down-regulated Cluster 1 genes were enriched in chromatin remodeling, while the m6A up-regulated Cluster 3 genes were associated with cell junction assembly and synapse organization (Supplementary Fig. 5D). It would be interesting to evaluate the regulation of *Mat2b* and m6A levels in different cell types. We may not be able to perform *Mat2b* knockout and m6A sequencing for every type of cancer, but we have examined the relationship between *Mat2b* expression and m6A levels in various cancer types. We obtained the gene expression and m6A sequencing data from the GEO database. To evaluate the m6A levels directly influenced by MAT2B, we generated plots for genes in Cluster 1 and Cluster 3. These genes’ m6A levels were restored by re-expressing MAT2B in *Mat2b* knockout cells. Our findings revealed that the m6A levels of these genes were associated with the expression levels of *Mat2b* (Supplementary Fig. 5E, F). When *Mat2b* was highly or lowly expressed, the m6A levels were correspondingly high or low. This data suggests that the role and mechanism of MAT2B in maintaining RNA m6A levels could be a common trend across different types of cancer.

Because m6A modification regulates mRNA stability [41, 42], we further analyzed how mRNA decay was regulated in these cells. The half-life of m6A down-regulated Cluster 1 genes decreased in *Mat2b* KO cells. This decrease in half-life was reversed by re-expressing WT MAT2B, but not by re-expressing MAT2BΔ35-41 which lacked the NADP^+^ binding domain (Fig. 4D, E). For m6A up-regulated Cluster 3 genes, we did not observe such a trend in half-life changes (Fig. 4F, G). More genes had a decreased half-life in Cluster 1, whereas more genes had an increased half-life in Cluster 3 (Supplementary Fig. 5G). Furthermore, in m6A down-regulated Cluster 1 genes, changes in m6A and half-life were highly correlated between WT and *Mat2b* KO cells (Fig. 4H). This high correlation was also observed between *Mat2b* KO and WT MAT2B re-expressed cells, but not between *Mat2b* KO and MAT2BΔ35-41 re-expressed cells (Fig. 4I and Supplementary Fig. 5H). The m6A up-regulated Cluster 3 genes did not show a strong correlation in changes of m6A and half-life under these conditions (Fig. 4J, K and Supplementary Fig. 5I). We validated the changes of half-life for four genes in Cluster 1 using RT-qPCR (Supplementary Fig. 5J). m6A modulates mRNA stability by interacting with reader proteins YTHDF2 and IGF2BP1/2/3. YTHDF2 promotes RNA degradation while IGF2BP1/2/3 represses it [42, 43]. Compared with YTHDF2, IGF2BP1/2/3 interacted with more m6A down-regulated Cluster 1 genes (Supplementary Fig. 5K, L). It’s possible that the reduction of m6A in Cluster1 genes prevented IGF2BP1/2/3 from repressing RNA degradation, leading to a shorter half-life in *Mat2b* KO cells. Together, these findings suggest that NADP^+^, by regulating the interaction between MAT2B and MAT2A, controls m6A and stability of mRNA.

### NADP^+^ regulates the tumorigenesis of liver tumors

In liver tumor progression, there is a transition of gene expression from *Mat1a* to *Mat2a* [12]. The up-regulation of *Mat2a* mRNA levels was believed to be a causal effect of tumorigenesis. Additionally, we observed up-regulation of *Mat2a* and *Mat2b* mRNA levels in liver tumors (Supplementary Fig. 6A). Based on these findings and previous reports, we opted to validate the role of MAT2B/m6A modification in liver cancer. The higher expression levels of *Mat2a* were significantly linked to shorter overall survival times in liver tumors, while the expression levels of *Mat2b* were not (Supplementary Fig. 6B). Furthermore, the expression levels of *Mat2a* and *Mat2b* did not show significant changes from patients with stage I disease to patients with stage IV disease (Supplementary Fig. 6C). This suggests that the expression levels of MAT2A and MAT2B may not be closely correlated with the formation of liver tumors.

**Fig. 5:**
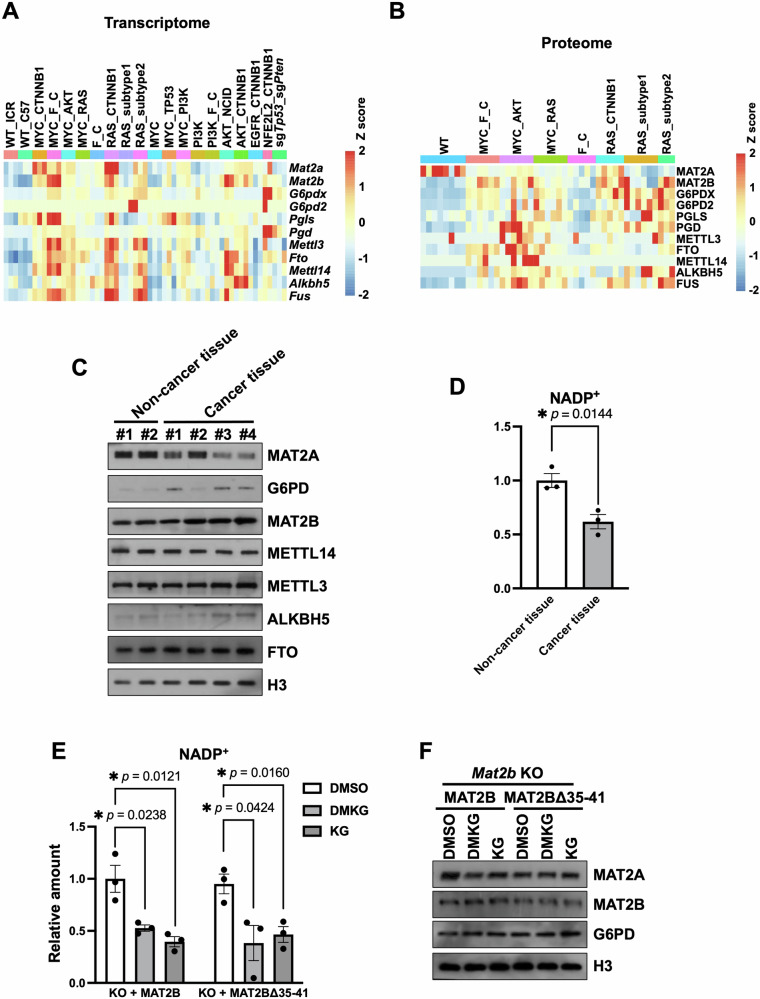
The protein level of MAT2A is decreased in liver tumors. The heatmaps showing the transcriptome (**A**) and proteome (**B**) in different mouse tumor models. WT, wild type mice. ICR and C57 were two breeds of mice. **C** Western blotting showing the protein levels between non-cancer tissue and cancer tissue in *Myc-Ctnnb1* driving tumor model. The tissues were ground with steel balls and boiled with SDS loading buffer. **D** The relative amounts of NADP^+^ in non-cancer tissue and cancer tissue in *Myc-Ctnnb1* driving tumor model. The amounts of NADP^+^ were detected by Coenzyme II NADP(H) Content Assay Kit (Sangon Biotech, Cat. #D799249). The contents of NDAP^+^ in non-cancer tissue were normalized as 1. The data were represented by the mean ± SEM (N = 3 independent replicates). The *p* value was determined by two-sided paired t-test. **E** The relative amounts of NADP^+^ in MAT2B rescued and MAT2BΔ3541 rescued U2OS cells treated with DMSO, KG (alpha-Ketoglutaric, 5 mM for 48 h) and DMKG (dimethyl-α-ketoglutarate, 5 mM for 48 h). The amounts of NADP^+^ were detected by Coenzyme II NADP(H) Content Assay Kit (Sangon Biotech, Cat. #D799249). The contents of NDAP^+^ in WT MAT2B rescued U2OS treated with DMSO were normalized as 1. The data were represented by the mean ± SEM (N = 3 independent replicates). The *p* value was determined by two-sided paired t-test. **F** Western blotting results showing MAT2A decreased in WT MAT2B rescued but not MAT2BΔ35-41 rescued U2OS cells after being treated with KG and DMKG. Cell extracts were analyzed by Western blotting using the indicated antibodies. The assay was repeated three times with similar results.

We then analyzed transcriptome and proteome results of previously reported mouse liver tumor models [44], which were generated by hydrodynamic tail vein injection (HTVI). The expressions of *Mat2a* and *Mat2b* were increased in almost all models (Fig. 5A). Surprisingly, the protein levels of MAT2A were largely reduced, even though there was an increase in MAT2B protein/mRNA and *Mat2a* mRNA (Fig. 5B). More importantly, we observed an increase in G6PDX, the homologous mouse protein of G6PD, in mouse liver tumors (Fig. 5B). We have found that the levels of NADP^+^ are critical for regulating the stability of the MAT2A protein by MAT2B. An increase in G6PDX could lead to a decrease in NADP^+^, causing a reduced interaction between MAT2A and MAT2B and ultimately leading to the depletion of MAT2A. *G6pd2*, an allele of *G6pdx*, was barely detected by RNA-seq, and mass spectrometry failed to detect its protein, indicating its gene expression and protein level were very low. Because we found that MAT2B regulated mRNA m6A, we specifically analyzed these mouse models to look for tumors without significant changes of m6A methyltransferases or demethylases. Under this criterion, we found the mouse model generated by over-expression of *Myc* and *Ctnnb1* was suitable for functional studies of MAT2B in tumorigenesis (Fig. 5C). More importantly, the NADP^+^ was decreased in tumors compared with non-tumor liver tissue in this tumor model, likely caused by the elevated G6PD (Fig. 5D).

In addition, previous reports have shown that α-Ketoglutarate (KG) reduced the glycolysis pathway in cells [45–47], it’s possible that KG would reduce the NADP^+^ levels by restricting the glycose-6-phosphate for PPP. Supporting this idea, KG and its esterified analog, dimethyl-α-ketoglutarate (DMKG) [48], decreased NADP^+^ (Fig. 5E). Moreover, the KG and DMKG treatment reduced MAT2A levels in WT or WT MAT2B re-expressed cells, leaving the MAT2A unchanged in MAT2BΔ35-41 re-expressed cells (Fig. 5F and Supplementary Fig. 7A). We then speculated that the keto diet (KD) could decrease NADP^+^ in conjunction with high G6PD, leading to a severe shortage of MAT2B-dependent MAT2A supplement and, subsequently, repression of tumorigenesis.

**Fig. 6:**
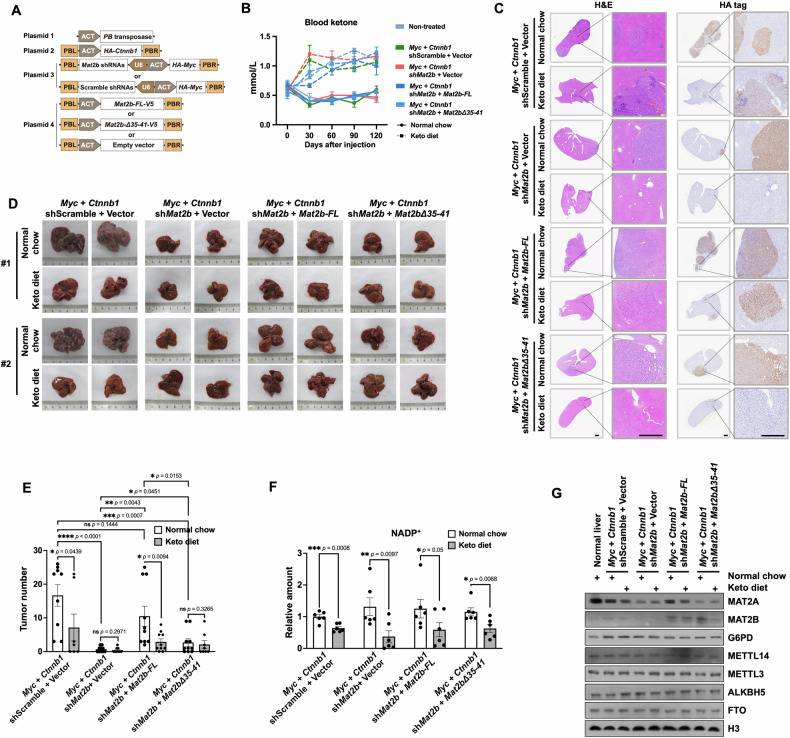
NADP^+^ regulates tumorigenesis in liver tumors. **A** Schema showing the plasmids used for mouse work. ACT, actin promoter. PB, piggyBac. PBL and PBR, piggyBac repeat termini. U6, U6 promoter. **B** The blood ketone levels of mice measured at intervals of 0, 30, 60, 90, and 120 days after the injection. The data were represented by the mean ± SEM (N = 3 independent mice). Normal chow, the control normal diet. The mice were fed different diets upon injection. **C** H&E and HA tag staining of mouse livers. Mouse livers from different cohorts were fixed, paraffin-embedded, sectioned, and stained. CTNNB1 and MYC were HA-tagged and could be stained by HA-tag staining. Representative livers were shown. Scale bars, 1 mm and 0.4 mm in scanned and zoom-in figures, respectively. **D** MAT2B functions in the tumorigenesis of liver tumors. High expressions of *Myc* and *Ctnnb1* in the liver were introduced into mice by HTVI. *Mat2b* was knocked down by shRNAs or re-expressed by full length (FL) or NADP^+^ binding domain truncationMAT2B (MAT2BΔ35-41). Mice were sacrificed around 120 days after injection and livers were pictured. Two representative livers in each cohort were shown. **E** The tumor number in different cohorts. 7–10 mice in each group were summarized. Data are mean ± SEM. The *p* value was determined by paired t-test, one-sided. **F** The relative amounts of NADP^+^ in different cohorts. The amounts of NADP^+^ were detected by Coenzyme II NADP(H) Content Assay Kit (Sangon Biotech, Cat. #D799249). The contents of NDAP^+^ in shScramble + Vector (*Myc* + *Ctnnb1* driven tumor model) cohort treated with normal chow were normalized as 1. The data were represented by the mean ± SEM (N = 6 independent replicates). The *p* value was determined by two-sided paired t-test. **G** Western blotting showing the indicated protein levels in different cohorts. Tissues were ground with steel balls and boiled with the SDS loading buffer.

Therefore, we used HTVI to introduce high expression of *Myc* and *Ctnnb1* into mouse livers. To demonstrate the function of *Mat2b* in tumorigenesis, we knocked down *Mat2b* and restored its expression with WT *Mat2b* or *Mat2bΔ35-41*, respectively. Four plasmids were used as a sleeping beauty system to express *Myc* and *Ctnnb1* together with *Mat2b* shRNAs and *Mat2b* cDNAs (Fig. 6A). After injection, every cohort was randomly divided into two groups to be fed with or without KD. KD treatment dramatically increased the blood keto and kept the blood sugar unchanged (Fig. 6B and Supplementary Fig. 7B). Additionally, KD treatment increased the fat content but had no impact on the weight of mice (Supplementary Fig. 7C, D).

Mice were sacrificed 120 days after injection and livers were pictured. H&E- and HA-stained liver sections confirmed tumor formation and HA-tagged MYC and CTNNB1 expression in tumors (Fig. 6C). While over-expression of *Myc* and *Ctnnb1* promoted tumorigenesis in mouse liver, knockdown of *Mat2b* largely repressed tumor formation. Restoring WT *Mat2b* expression fully restored tumorigenesis, while restoring *Mat2bΔ35-41* expression partially restored tumorigenesis (Fig. 6D, E). KD suppressed tumorigenesis in both non-target shRNA treated and WT MAT2B rescued mouse models. Most importantly, KD did not further suppress the tumorigenesis in MAT2BΔ35-41 re-expressed mice. Consistent with the tumor growth, KD treatment lowered NADP^+^ content in tumors (Fig. 6F). Accordingly, KD decreased protein levels of MAT2A in tumors with non-target shRNA or WT *Mat2b* re-expression, but had no effect on MAT2A in tumors with *Mat2b* depletion or *Mat2bΔ35-41* re-expression (Fig. 6G and Supplementary Fig. 7E). These data indicate that KD represses tumor formation, at least partially, through the MAT2B-dependent regulation of MAT2A.

In line with these observations, the expressions of *Mat2a, Mat2b*, and *G6pdx* were elevated in tumors, while *Mat2b* shRNA treatment reduced the expression of *Mat2b* (Supplementary Fig. 7F). Re-expression of *Mat2b* and *Mat2b*Δ35-41 restored the mRNA levels. The reduced tumor formation in MAT2BΔ35-41 rescued cohort was unlikely attributed to the different expression levels between WT and mutant *Mat2b*. In addition to the protein levels of analyzed m6A-associated enzymes, mRNA m6A levels were not altered by KD treatment (Supplementary Fig. 7G). Together, these data suggest that NADP^+^ is critical for liver tumor formation, and KD repressed tumorigenesis, at least partially, via MAT2B-MAT2A interaction.

## Discussion

The control of SAM levels is crucial for many cell functions [49–52], but the process of SAM generation is not well understood. MAT2B has been known to be the regulator of MAT2A for several decades [10, 14, 53]. Previous studies have investigated the regulatory function of MAT2B through large amounts of in vitro assays. In vitro, the regulatory effect on catalytic enzymes is usually dependent on two mechanisms: substrate affinity and inhibition from catalytic products. As a result, previous researches have indicated that MAT2B can regulate MAT2A’s activity by altering its affinity toward substrates and sensitivity toward product inhibition [17, 22, 54]. Nevertheless, further studies have revealed that the binding of MAT2B with MAT2A does not considerably affect its catalytic activity or the physiological inhibition of SAM [20]. Here, we find that in addition to the regulation of enzymatic activity, MAT2B can regulate the protein level of MAT2A in an NADP^+^-dependent manner. Disruption of MAT2B-MAT2A interaction results in the retention of m6A levels. Moreover, MAT2B is critical for the tumorigenesis of liver cancer and KD repressed the tumor formation through, at least partially, the MAT2B-MAT2A interaction. We propose that MAT2B is a central factor in linking SAM synthesis to RNA m6A, through which the m6A level of mRNA is safeguarded.

Various trans-activating factors such as Sp1, c-Mybl2, NF-kB, and AP-1 participate in *MAT2A* transcriptional upregulation in hepatocellular carcinoma (HCC) [55]. However, the regulatory mechanisms underlying MAT2B transcription in HCC remain insufficiently characterized. In the HepG2 liver cancer cell line, TNF-α upregulates MAT2B mRNA through AP-1 and NF-κβ pathways [56]. Sirtuin 1 (SIRT1), a NAD^+^-dependent deacetylase, also induces MAT2B expression [57], while leptin modulates MAT2B expression via ERK and AKT signaling pathways [58]. Sp1 has been identified as a critical activator of the MAT2B promoter [14]. Despite these findings, the regulation of MAT2B expression, particularly the mechanisms governing its variant-specific expression in HCC, remains poorly understood. Previous studies have primarily focused on the mRNA level of MAT2A rather than the protein level. Our findings indicate that while the mRNA level of MAT2A is increased in liver tumors, the protein level is decreased due to the overexpression of G6PD and subsequent reduction in NADP + . Our data aligns with previous research showing that increased MAT2A mRNA may have an oncogenic effect. In liver tumors, G6PD restricts the protein level of MAT2A. Further reduction in MAT2A levels is likely to inhibit tumorigenesis, as the total MAT1/2A protein levels are too low to produce sufficient SAM.

Depletion of MAT2A decreases the content of SAM and methylation of histones and RNA. Although depletion of MAT2B represses the protein level of MAT2A, the content of SAM and methylations of histones and RNA are unchanged. Previous studies show that MAT2B increases MAT2A’s sensitivity to product inhibition [18]. It’s possible that, when MAT2B is depleted, the inhibitory effect of SAM on MAT2A’s catalytic activity is repressed, leading to the retention of SAM levels. Through this regulation, cells generate sufficient SAM to maintain m6A and stability. In addition, when MAT2B is up-regulated to increase MAT2A levels, the inhibitory effect of SAM on MAT2A’s catalytic activity is elevated, resulting in a stable SAM level and m6A. Cells can get ready to generate more SAM to provide a rapid regulation of m6A and, at the same time, repress the overactivation of MAT2A to generate exceeded SAM. Similar to the MAT proteins, SAM-dependent methylases utilize SAM as a substrate and generate S-adenosyl homocysteine (SAH) as a product. Interestingly, SAH acts as a robust inhibitor for nearly all SAM-dependent methylases, despite their varied biological functions [59]. This intricate interplay between SAM and SAH establishes a feedback mechanism that effectively regulates the enzymatic activity of SAM-dependent methylases.

Previous research indicates that NADP^+^ plays a role in regulating FTO [60]. Additionally, reducing the levels of MTHFD1, ME1, or ME2 has been shown to decrease the amount of FTO protein and enzymatic activity, ultimately affecting m6A. In our study, we find that knocking down *G6pd* does not affect the protein level of FTO. This is likely because the metabolism pathway varies among different cell lines. Nevertheless, since manipulating G6PD leaves FTO unchanged, we are able to detect changes in m6A that were not dependent on FTO.

Regardless of the reason why the MAT2B-regulated MAT2A affects RNA m6A, we propose that, compared to histone methylation and DNA methylation, RNA methylation, which is much more dynamic in regulating gene expression and RNA decay, requires frequent turnover. However, it is possible that MAT2B controls MAT2A’s enzymatic activity, leading to confounding effects on RNA m6A regulation. In any case, further understanding of the mechanisms involving MAT2B will provide insights into SAM homeostasis and RNA m6A regulation.

## Methods

### Cell culture and cell lines

U2OS, Astrocyte, HEK293T, LN229, HepG2, and SF9427 were cultured in DMEM (Thermofisher Scientific, Cat. #C11965500BT) supplemented with 1% glutamax, 10% FBS, and 1% antibiotic solution (penicillin/streptomycin). U87 and U251 were cultured in MEM (Thermofisher Scientific, Cat. #41500018) supplemented with 1% glutamax, 10% FBS, 1% antibiotic solution (penicillin/streptomycin), 1% sodium pyruvate, and 2.2 g/L sodium bicarbonate. All cells were cultured at 37 °C with 5% CO_2_. U2OS, HEK293T, LN229, HepG2, U87, and U251 were from ATCC and Astrocyte was from Dr. Jennifer Westendorf. All cells were authenticated by RNA seq. All cell lines were negative for mycoplasma contamination.

*Mat2b* KO cell lines were constructed by sgRNAs, which were cloned in pSpCas9(BB)-2A-Puro from Dr. Feng Zhang (Addgene plasmid #48139). shRNAs against *Mat2a, Mat2b, Nadk, Nadk2, G6pd, 6pgd, Mthfd1, Me1, Me2*, and *Acmsd* were constructed into pLKO.1 vector using primers listed in Supplementary Table S2.

### Protein purification

The wild-type and mutant *Mat2a*, and *Mat2b* cDNA were cloned into GFP-His-tagged pET28a-smt3, His-tagged pET28a-smt3, and GST-tagged pGEX-6p-2 vector respectively. The plasmids were transformed into *E.coli* BL21 cells. Cells were grown to OD600 around 0.6 before 0.1 mM isopropyl β-d-1-thiogalactopyranoside (IPTG) was added to induce protein expression [61]. The cell lysates were bound with homemade GFP or NI-NTA (Sangon Biotech, Cat. #C600033) or GST Seflnose Resin beads (Sangon Biotech, #C600031) at 4 °C for 2 to 4 h. The beads were washed extensively. The GFP Seflnose Resin beads bound with MAT2A or MAT2A mutants were used for in vitro pull-down.

### In vivo immunoprecipitation

Immunoprecipitation was performed as described before [61]. Cells were lysed in RIPA buffer (50 mM Tris-HCl pH 7.5, 150 mM NaCl, 1% NP-40, 0.5% sodium deoxycholate, 0.1% SDS, 1 mM EDTA, and 1× protease inhibitor). The lysates were dounced with 50 passages followed by rotation at 4 °C for 0.5 h. Samples were centrifugated at 12,000 *g* for 15 min at 4 °C. 5% of supernatants were taken as input and boiled with SDS loading buffer. Pre-washed FLAG-Seflnose Resin beads (Sigma, Cat. #M8823) were added into the supernatants and rotated at 4 °C overnight. Then the samples were washed by washing buffer (50 mM HEPES pH 7.4, 100 mM NaCl, 0.01% NP-40, 10% glycerol, and 1 mM EDTA) 5 times before being boiled for Western blotting.

### Quantitative reverse transcription PCR

Total RNA was extracted with TRIzol reagent (Sangon Biotech, Cat. #B610409) and was reversely transcribed into cDNA by HiScript II Q RT SuperMix Kit according to the manufacturer’s instructions (Vazyme, Cat. #R222-01). cDNA was proceeded for quantitative PCR with SYBR Green (Yeasen, Cat. #11201ES03) by using Quantagene q225 qPCR system (Kubo Technology, Beijing) as described before [62, 63]. *β-actin* was used as a control to normalize the expression of other target genes. The 2^-(△-△control)^ method was used to represent the relative gene expression. Primers used were listed in Supplementary Table S2.

### Mass spectrometry analysis

For the detection of NADP(H), NAD(H), and SAM, cells were equally seeded into 12-well plates the day before extraction. The dishes were washed with ice-cold PBS once and briefly soaked in liquid nitrogen. 1 ml −40 °C 75% mass spectrometry grade ethanol was added into the dish to transfer cells into 1.5 ml screw-cap tubes. After heat blocking at 75 °C for 3 min, the samples were centrifugated at 15,000 *g* at 4 °C for 10 min twice. The supernatants were dried and resuspended with 5 mM ammonium acetate followed by 6 rounds of vigorous 30 s vortex and 30 s on ice. After being incubated on ice for 30 min, the samples were centrifugated at 15,000 *g* at 4 °C for 10 min twice. The supernatants were then collected for HPLC/LC-MS.

For the detection of m6A in mRNA, the total RNA was extracted with TRIzol reagent (Sangon Biotech, Cat. #B610409), and mRNA was further purified by firstly heated at 65 °C for 5 min and quickly chilled on ice for 2 min, followed by adding samples into the binding buffer (0.5 M NaCl, 20 mM Tris-HCl pH 7.5, and 1 mM EDTA) containing Streptavidin Magnetic Beads (Smart-Lifesciences, Cat. #SM01710) which were coupled with oligo dT. After incubation at room temperature for 10 min, the beads were washed with binding buffer twice and low salt buffer once (0.15 M NaCl, 20 mM Tris-HCl pH 7.5, and 1 mM EDTA), which were then resuspended with water. Samples were incubated at 94 °C for 15 min and immediately chilled on ice for 1 min. After the removal of beads, the solution was purified as mRNA. For the detection of 5mC in DNA, genomic DNA was extracted with TRIzol reagent (Sangon Biotech, Cat. #B610409) according to the manufacturer’s instructions. mRNA and DNA were digested by Nuclease S1 (Sigma, Cat. #N5661-50KU, ~212 U/μl) into nucleotides for HPLC/LC-MS.

### Dot blot

mRNA was dropped onto the Nylon membrane. After 20 min UV irradiation, the membrane was blocked with 5% bovine serum albumin (Sangon Biotech, Cat. #A602440 BSA) in TBS-T at room temperature for 1 h followed by the incubation with m6A primary antibody at 4 °C overnight. After washing with TBS-T for three times, the membrane was incubated with the secondary antibody at room temperature for 1 h followed by three additional washes with TBS-T. The m6A signals were detected using an ECL detection reagent.

To further detect the amount of spotted mRNA, the membrane was washed with 100% ethanol for 5 min followed by water and 0.05% (w/v) methylene blue (Aladdin, Cat. #M134389) staining for 5 min. The dots could be seen after distaining with water several times.

### RNA decay

RNA decay assay was performed as described [64]. The cells were seeded into different wells of 6-well plates. 10 μg/ml of Actinomycin D (Sigma, Cat. #A9415) was added into each well once the cells were fully adherent to the plates. Samples were collected at intervals of 0, 3, and 6 h after drug treatments for RNA extraction. RNA was then reversely transcribed into cDNA by HiScript II Q RT SuperMix Kit (Vazyme, Cat. #R222-01) and HiScript II 1st Strand cDNA Synthesis Kit (Vazyme, Cat. #R211-01) for qPCR or library construction respectively according to the manufacturer’s instructions.

### Protein degradation

Cells were seeded into 6-well plates with an equal number in each well. After 24 h, 25 μg/ml of cycloheximides (Aladdin, Cat. #C112766) was added into each well, and the cells were collected into SDS loading buffer at intervals of 0, 2, 5, and 10 h after drug treatments.

### Microscale Thermophoresis (MST)

Protein-molecule interactions were analyzed by MST. MAT2B and MAT2BΔ35-41 were labeled with fluorescein isothiocyanate (FITC) and dissolved in MST buffer (PBS containing 0.5% BSA and 0.05% Tween-20) with a concentration of 50 nM. NADP^+^ (Sangon Biotech, Cat. #A600760) and NADPH (Aladdin, Cat. #N276326) were also dissolved in MST buffer and diluted from 2.5e-5 to 50 mM. Assays were performed with the NT.115 Monolith instrument (Nano Temper Technologies) using a blue LED for excitation in three independent replicates.

### NADP(H) detection

Besides HPLC/LC-MS, Coenzyme II NADP(H) Content Assay Kit (Sangon Biotech, Cat. #D799249-0059) was also used to detect NADP(H). The cells and tissues were lysed by NADP(H) extraction buffer followed by sonication or vigorous vortex respectively, and then corresponding reagents were added into samples in sequence according to the manufacturer’s instructions. The contents of NADP(H) were determined by the absorption at 570 nm.

### Animal model

The hydrodynamic tail-vein injection was performed as reported previously [65, 66]. The experiments were not blinded as the identities of treatment and food. Liver tumors were induced by transposon-mediated integration and expression of HA-tagged oncogenes *Myc* and *Ctnnb1*. shRNAs were then expressed by the U6 promoter in tandem with *Myc*. For MAT2B rescue, V5-tagged mouse MAT2B and MAT2BΔ35-41 were expressed by ACT promoter in an individual transposon plasmid. Plasmids used for hydrodynamic tail-vein injection were prepared by FastPure EndoFree Plasmid Maxi Kit according to the manufacturer’s instructions (Vazyme, Cat. # #DC202-01) and were dissolved in sterile Ringer’s solution (5.6 mM KCl, 154 mM NaCl, 2.2 mM CaCl_2_, and 2.4 mM NaHCO_3_) equal to 10% of mice body weight. 62.5 μg of total transposon plasmids and 17 μg piggyBac transposase plasmids were delivered. Mice were sacrificed around 120 days after injection. Livers were pictured, weighted, and fixed or snap-frozen in liquid nitrogen for further usage. In this study, four-week-old male specific-pathogen-free ICR mice were purchased by Shanghai SLAC Laboratory Animal Company and kept in SPF facilities at Zhejiang University Laboratory Animal Center. All the mice study methods were approved by Zhejiang University Animal Care and Use Committee.

### Hematoxylin-Eosin staining

The paraffin slides were deparaffined and rehydrated by xylene and alcohol gradients. Tissues were stained with Hematoxylin staining solution for 5 min, incubated with 0.1% hydrochloric acid-ethanol for 10 s followed by staining with Eosin for 30 s. Slides were dehydrated in alcohol and xylene gradients and then sealed for future usage. The slides were scanned under the digital section scanner (KFBio, KF-PRO-005).

### Immunohistochemistry staining

Immunohistochemistry (IHC) staining of paraffin sections was performed by the avidin-biotin complex (ABC) method as previously described [67]. The paraffin slides were deparaffined and rehydrated by xylene and alcohol gradients. The slides were boiled in citrate buffer (pH 6.0) for antigen retrieval followed by incubation in 2% H_2_O_2_/distilled H_2_O for 30 min and blocking in 10% goat normal serum with Avidin for 30 min. Primary Rabbit monoclonal anti-HA-tag antibody was added by 1:200 dilution with biotin and incubated at 4 °C overnight. Avidin and biotin were from the Avidin/Biotin Blocking kit (Vector laboratories, Cat#SP-2001). After washing with PBS 3 times, the slides were treated by VECTASTAIN® Elite ABC-Peroxidase kits according to the manufacturer’s instructions (Vector laboratories, Cat#PK-6100) for 30 min. Afterwards, slides were stained with peroxidase substrate solution, DAB kit (Sangon Biotech, Cat. #E670033), for 2 to 10 min before being stopped with PBS. In addition, slides were stained with hematoxylin, de-stained with hydrochloric acid-ethanol, and reversed blue in ammonium solution. Slides were dehydrated in alcohol and xylene gradients, sealed with a mounting medium, and then analyzed under the digital section scanner (KFBio, KF-PRO-005).

### Blood sugar and blood ketone detection

Blood was collected from mouse tails at intervals of 0, 30, 60, 90, and 120 days after injection. Around 0.6 μl blood was dropped into blood sugar or ketone test strips respectively, and the strips were inserted into the Blood Glucose and Ketone Monitoring System (Abbott, FreeStyle Optium Neo) to measure the concentration of blood sugar or ketone.

### Contents of mice body detection

Around 120 days after plasmid injection, mice were taken into the Animal Body Composition analyzer (NIUMAI, QMR06-090H-PRO), and the weights of fat, muscle, and free water were detected.

### Mitochondrial isolation

Cells were washed with cold PBS twice and lysed with ice-cold IBcells-1 (225 mM mannitol, 75 mM sucrose, 0.1 mM EGTA, and 30 mM Tris–HCl pH 7.4). Samples were homogenized with teflon pestle for 50 strokes, and centrifuged at 600 g for 5 min at 4 °C. After removal of the pellets, the sample was centrifuged at 7000 *g* for additional 10 min at 4 °C. The supernatants and pellets were cytoplasm extraction and mitochondrial extraction, respectively.

### Antibodies

Rabbit polyclonal anti-Histone H3 (Abcam, Cat. #ab1791); Rabbit polyclonal anti-MAT2B (Thermofisher Scientific, Cat. #703221); Rabbit polyclonal anti-MAT2A (NOVUS, Cat. #nb110-94158); NADK (Cell Signaling Technology, Cat. #55948); NADK2 (Abcam, Cat. #ab181028); Mouse monoclonal anti-FLAG(GenScript, Cat. #A00187); Rabbit polyclonal anti-FLAG Tag (Sangon Biotech, Cat. #D110005); Rabbit polyclonal anti-Histone H3K36me3 (Cell Signaling Technology, Cat. #4909); Rabbit polyclonal anti-Histone H3K4me3 (Abcam, Cat. #ab8580); Rabbit monoclonal anti-Histone H3K27me3 (Cell Signaling Technology, Cat. #9733); Rabbit polyclonal anti-Histone H3K27ac (Abcam, Cat. #ab4729); Rabbit polyclonal anti-Histone H3K9me3 (Abcam, Cat. #ab8898); Acetylated-Lysine Antibody(pan-ac) (Cell Signaling Technology, Cat. #9441); Rabbit monoclonal anti-P300 (Santa Cruz BiotechnologyInc, Cat. #sc585); mouse monoclonal anti-β-Tubulin (Cell Signaling Technology, Cat. #86298); Rabbit polyclonal anti-Histone H4K20me3 (Abcam, Cat. #ab9051); Rabbit polyclonal anti-Histone H3K4me1 (Abcam, Cat. #ab8895); Rabbit polyclonal anti-METTL3 (Cell Signaling Technology, Cat. #86132); Rabbit polyclonal anti-METTlL14 (Cell Signaling Technology, Cat. #51104); Rabbit polyclonal anti-FTO (Cell Signaling Technology, Cat. #31687); Rabbit polyclonal anti-ALKBH5 (Cell Signaling Technology, Cat. #80283); Rabbit polyclonal anti-MTHFD1 (Santa Cruz BiotechnologyInc, Cat. #sc-271412); Rabbit polyclonal anti-ME1 (Santa Cruz BiotechnologyInc, Cat. #sc-365891); Rabbit polyclonal anti-ME2 (Santa Cruz BiotechnologyInc, Cat. #sc-514850); Rabbit monoclonal anti-HA Tag C29F4A (Cell Signaling Technology, Cat. # 3724); Rabbit polyclonal anti-G6PD (Cell Signaling Technology, Cat. #12263); Anti-N6-methyladenosine (m6A) (Abcam, Cat# ab284103); Peroxidase AffiniPure Goat anti-Rabbit IgG (H + L) (Peroxidase AffiniPure Goat anti-Rabbit IgG (H + L), Cat. #111-035-003); Peroxidase AffiniPure Goat anti-mouse IgG (H + L) (Peroxidase AffiniPure Goat anti-Rabbit IgG (H + L), Cat. #115-035-003).

### MeRIP-Seq

Total RNA was subjected to RiboMinus treatment according to the manufacturer’s instructions (Invitrogen). RNA was then fragmented into 100 nts using Vazyme Fragmentation Buffer according to the manufacturer’s instructions and was subjected to m6A immunoprecipitation. Sequencing libraries were prepared using the RNA-seq library prep kit (VAHTS, Cat. #NR604).

### RIP-seq analysis

Raw reads were trimmed to remove adapters and low-quality sequences using Trim Galore (version 0.6.6) (https://www.bioinformatics.babraham.ac.uk/projects/trim_galore/) with the parameter ‘-q 20 --paired’. The trimmed reads were then uniquely mapped to the human genome (hg19) using STAR [68] (version 2.7.7a). The output SAM files were converted to sorted BAM files using SAMtools [69] (version 1.11). Expression matrixes were counted by featureCounts [70] (version 2.0.1) and normalized as fragments per kilobase per million mapped reads (FPKM) by StringTie [71] (version 2.1.7). R package DESeq2 [72] (version 1.34.0) was used to find genes with different expression levels, which were filtered by the threshold ‘*p* < 0.05’. Genome coverage bigwig files were generated by BEDtools (version 3.5.0) genomecov and bedGraphToBigWig (version 4) (https://www.encodeproject.org/software/bedgraphtobigwig/) with the parameter ‘-scale 10,000,000/mapped_reads_number -split’. A 1 Kb sliding window across the whole genome was used to calculate the Pearson product moment correlation. Gene Ontology (GO) analysis was performed by clusterProfiler [73] (version 4.2.2).

For peak calling, repeats were merged and trimmed reads were uniquely mapped to the human reference genome (hg19) using Bowtie2 [74] (version 2.4.2). Peaks were identified using MACS2 [75] (version 2.2.7.1) with the parameters ‘--nomodel -f BAMPE -g hg --keep-dup 5’. Peak annotation was performed according to the gene positions extracted from UCSC genome gtf. Genome coverage bigwig files were generated by BEDtools genomecov and bedGraphToBigWig. Aggregation plots of coding genes were generated by deepTools [76] (version 3.5.0) computeMatrix and plotProfile with the parameter ‘--metagene’, whose bed files of 5’ UTR, CDS and 3’ UTR were extracted from gtf. The Venn plots were generated by the R package VennDiagram (version 1.7.1) (https://github.com/cran/VennDiagram).

### Lifetime-seq analysis

Adaptors and low-quality reads were removed by Trim Galore with the parameter ‘-paired’. Trimmed reads were then mapped to the human genome (hg19) using STAR and *E.coli* spike-in reads were mapped using Bowtie2. Expression matrixes were normalized as fragments per kilobase per million mapped reads (FPKM) by StringTie. FKPM were converted to attomole by linear-fitting of the *E.coli* spike-in reads. The half-life of RNA was estimated using the method described before [77]. Specifically, as actinomycin D was a potent transcription inhibitor, the rate of RNA concentration changes at a given time (${dC}/{dt}$) was proportional to the constant of RNA decay (${K}_{{decay}}$) and the RNA concentration ($C$). This relation was described by the following equation:

$$\frac{{dC}}{{dt}}=-{K}_{{decay}}C$$

Thus, the RNA degradation rate ${K}_{{decay}}$ was estimated by:

$${\mathrm{ln}}\left(\frac{C}{{C}_{0}}\right)=-{K}_{{decay}}t$$

To calculate the RNA half-life (${t}_{1/2}$) when 50% of the RNA was decayed (that was $\frac{C}{{C}_{0}}=\frac{1}{2}$), the equation was:

$${\mathrm{ln}}\left(\frac{1}{2}\right)=-{K}_{{decay}}{t}_{\frac{1}{2}}$$

From this we got:

$${t}_{\frac{1}{2}}=\frac{\mathrm{ln}2}{{K}_{{decay}}}$$

### CLIP-seq binding sites

m6A readers regulated RNA stability in an m6A-dependent manner [41, 42, 78]. To determine the enrichment of m6A readers, CLIP-seq binding sites of YTHDF2, IGF2BP1, IGF2BP2, and IGF2BP3 were obtained from POSTAR3 CLIPdb [79] with the option ‘PAR-CLIP, Piranha_0.01’.

### Sample size

The sample and animal sizes were determined based on prior published data from similar experiments [66]. Animals were randomized before being treated based on the similar weight.

## Supplementary information

Supplementary information

Raw WB Data

## Data Availability

The raw sequence data reported in this paper have been deposited in the Genome Sequence Archive in the National Genomics Data Center, China National Center for Bioinformation/Beijing Institute of Genomics, Chinese Academy of Sciences (GSA-Human: HRA005698) that are publicly accessible at https://ngdc.cncb.ac.cn/gsa.
