# Supplementary material for: MAT2B regulates the protein level of MAT2A to preserve RNA N6-methyladenosine
Source: Cell Death Dis. 2024 Oct 1;15(10):714. doi: 10.1038/s41419-024-07093-8 (PMC11445541; doi:10.1038/s41419-024-07093-8)
Supplement: Supplementary file 1 — Supplementary information [file 41419_2024_7093_MOESM1_ESM.pdf]

MAT2B regulates the protein level of MAT2A to preserve RNA m6A methylation

Xinyi Wan[1, 5], Weiwu Zeng[1, 5], Haonan Fan[1, 5], Chenliang Wang[2], Shixun Han[2], Zhongxing Sun[1], Mei Tang[2], Juejia Shao[1], Yu Liu[1], Yuan Fang[1], Junqi Jia[1], Yin Tang[1], Yanjun Zhang[1], Bin Zhao[2], Dong Fang[1, 3, 4, *]

[1] The Second Affiliated Hospital of Zhejiang University School of Medicine, Life Sciences Institute, Zhejiang University, Hangzhou, China.

[2] Life Sciences Institute, Zhejiang University, Hangzhou, China.

[3] Department of Medical Oncology, Key Laboratory of Cancer Prevention and Intervention, Ministry of Education, The Second Affiliated Hospital, Zhejiang University School of Medicine, Hangzhou, Zhejiang, China.

[4] Key Laboratory of Cancer Prevention and Intervention, China National Ministry of Education.

[5] These authors contribute equally

[*] Correspondence: dfang@zju.edu.cn

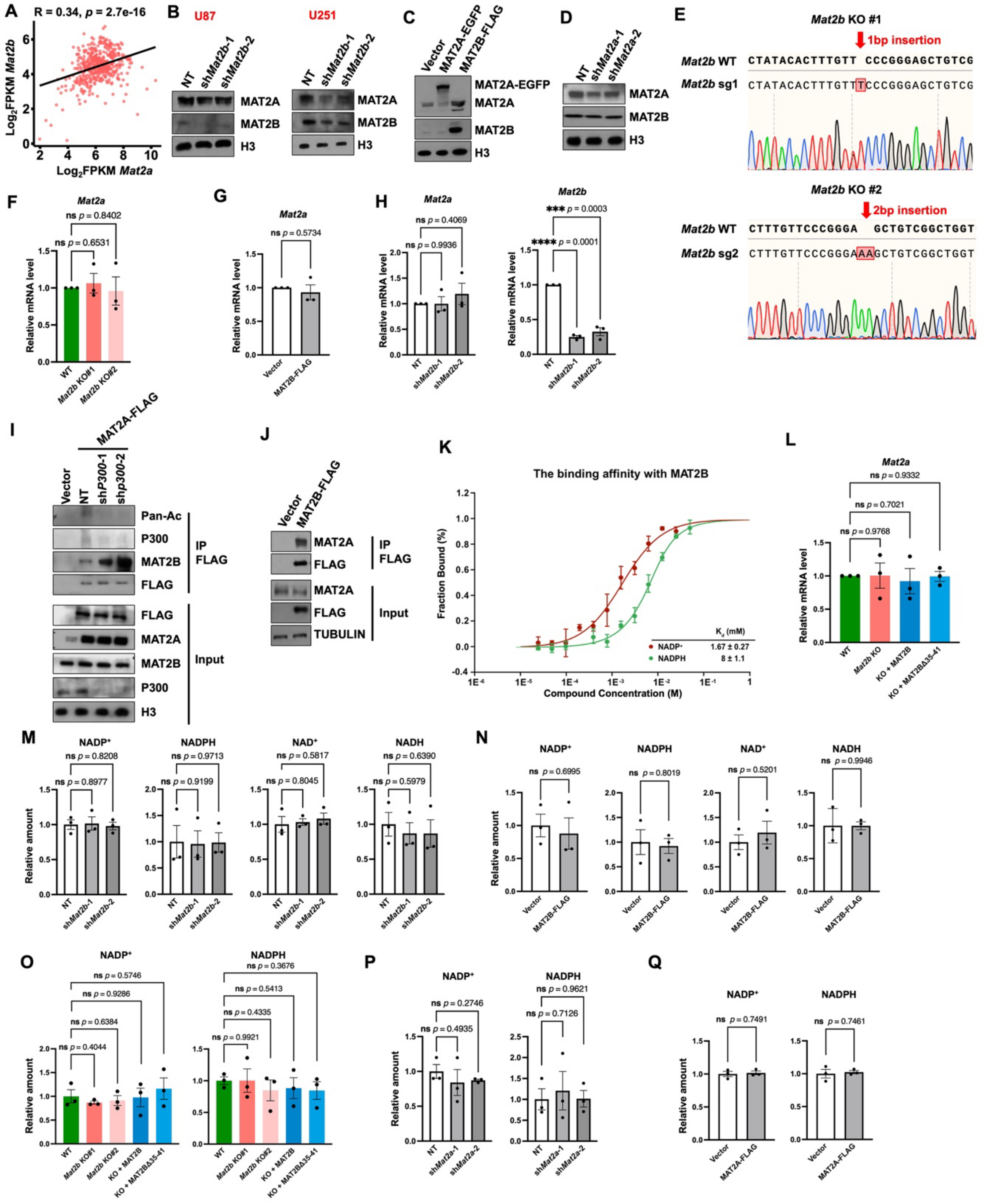

Supplementary Figure 1. Manipulating MAT2B changes the protein level of MAT2A.

(A) Correlation between *Mat2a* and *Mat2b* expression levels, based on RNA-Seq data from tissues, cell lines, and primary cells in the ENCODE project. FPKM, fragments per kilobase per million mapped reads. R, correlation coefficients that were assessed by Pearson product-moment correlation. The p values were calculated by a two-sided paired t-test.

(B) Western blotting results showing MAT2A and MAT2B protein levels after MAT2B depletion in U87 and U251 cells. *Mat2b* was knocked down by two independent shRNAs. NT, non-target control. The assay was repeated twice with similar results.

(C) Western blotting results showing that the protein level of MAT2A increased after MAT2B over-expression and MAT2B remained unchanged after MAT2A over-expression in U2OS cells. The assay was repeated three times with similar results.

(D) Western blotting results showing that MAT2B remained unchanged after MAT2A depletion. *Mat2a* was knocked down by two independent shRNAs in U2OS cells. The assay was repeated twice with similar results.

(E) The Sanger sequencing results of two *Mat2b* KO U2SO cell lines, which were generated by two independent sgRNAs. *Mat2b* KO #1 was generated through a 1-base pair insertion, while *Mat2b* KO #2 had a 2-base pair insertion.

(F) RT-qPCR results of the *Mat2a* gene expression in WT and *Mat2b* KO U2OS cell lines. Gene expressions were normalized to *β-actin* and the expression levels in WT cells were further normalized as 1. The data were represented by the mean ± SEM (N = 3 independent replicates). The *p* value was determined by two-sided paired t-test.

(G) RT-qPCR results of the *Mat2a* gene expression in empty vector and *Mat2b* expression vector transfected U2OS cells. Gene expressions were normalized to *β-actin* and the expression levels in cells transfected with empty vector were further normalized as 1. The data were represented by the mean ± SEM (N = 3 independent replicates). The *p* value was determined by two-sided paired t-test.

(H) RT-qPCR results of the *Mat2a* and *Mat2b* gene expression in MAT2B depleted U2OS cells. Gene expressions were normalized to *β-actin* and the expression levels in cells treated with NT (non-target) shRNA were further normalized as 1. The data were represented by the mean ± SEM (N = 3 independent replicates). The *p* value was determined by two-sided paired t-test.

(I) Depletion of P300 increased the interaction between MAT2A and MAT2B. MAT2A was purified by FLAG IP in HEK293T cells overexpressing FLAG-tagged MAT2A. HEK293T cells transfected with empty vectors were used as negative controls. Proteins from input and IP samples were analyzed by Western blotting using the indicated antibodies. The assay was repeated twice with similar results.

(J) MAT2B bound with MAT2A *in vivo*. MAT2B was purified by FLAG IP in HEK293T cells overexpressing FLAG-tagged MAT2B. Proteins from input and IP samples were analyzed by Western blotting using the indicated antibodies. The assay was repeated at least three times with similar results.

(**K**) MAT2B bound preferably with $NADP^+$ over NADPH. Microscale Thermophoresis (MST) assay was conducted with purified MAT2B. Data were mean ± SD (N = 3 independent experiments).

(L) RT-qPCR results of the *Mat2a* expression in WT, *Mat2b* KO, WT MAT2B rescued, and MAT2BΔ35-41 rescued U2OS cells. Gene expressions were normalized to *β-actin* and the expression levels in WT cells were further normalized as 1. The data were represented by the mean ± SEM (N = 3 independent replicates). The *p* value was determined by two-sided paired t-test.

(M and N) Mass spectrometry results showing that the total level of NADP(H) and NAD(H) remained unchanged after MAT2B depletion (M) or over-expression (N). The contents of individual compounds in NT or empty-vector treated cells were normalized as 1, respectively. The data were represented by the mean ± SEM (N = 3 independent replicates). The *p* value was determined by two-sided unpaired t-test.

(O - Q) Mass spectrometry results showing the total level of NADP(H) in *Mat2b* KO and rescued cells (O), MAT2A depletion cells (P), and MAT2A overexpression cells (Q). The contents of individual compounds in WT, NT, or empty-vector treated U2OS cells were normalized as 1, respectively. The data were represented by the mean ± SEM (N = 3 independent replicates). The *p* value was determined by two-sided unpaired t-test.

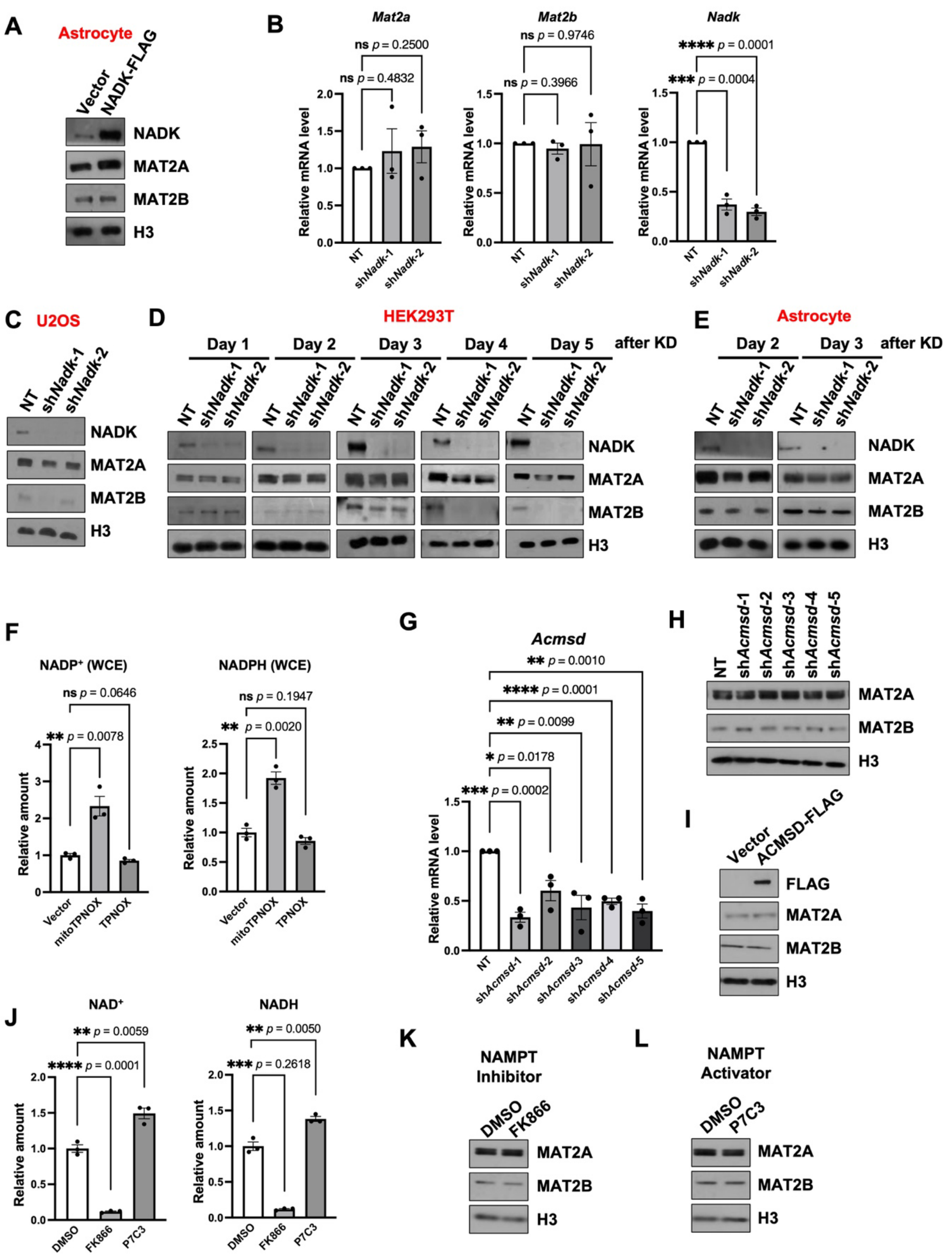

Supplementary Figure 2. NADP$^+$ is critical for the protein level of MAT2A.

(A) Western blotting results showing MAT2A and MAT2B protein levels after NADK-FLAG were over-expressed in astrocyte cells. The assay was repeated three times with similar results. **(B)** RT-qPCR analysis of *Mat2a*, *Mat2b*, and *Nadk* expression levels in *Nadk* knockdown U2OS cells. NT, non-target control Gene expressions were normalized to *β-actin* and the

expression levels in NT cells were further normalized as 1. The data were represented by the mean ± SEM (N = 3 independent replicates). The *p* value was determined by two-sided paired t-test.

(C) MAT2A and MAT2B protein levels were decreased after a prolonged depletion of NADK. The assay was repeated three times with similar results.

(D and E) Western blotting showing the protein levels of MAT2A and MAT2B after indicated days of NADK depletion in HEK293T (D) and astrocyte cells (E). NT, non-target control. These assays were repeated twice with similar results.

(F) NADP(H) increased with mitoTPNOX overexpression. The contents of individual compounds in empty-vector transfected U2OS, as determined by mass spectrometry, were normalized as 1. The data were represented by the mean ± SEM (N = 3 independent replicates). The *p* value was determined by two-sided unpaired t-test. WCE, whole cell extraction.

(G) Gene expression of *Acmsd* in NT and *Acmsd* knockdown U2OS as determined by RT-qPCR. *Acmsd* was knocked down by 5 independent shRNAs. NT, non-target control. Gene expressions were normalized to *β-actin* and the expression levels in NT cells were further normalized as 1. The data were represented by the mean ± SEM (N = 3 independent replicates). The *p* value was determined by two-sided paired t-test.

(H) Western blotting results showing MAT2A and MAT2B protein levels after *Acmsd* was knocked down in U2OS cells. The assay was repeated twice with similar results.

(I) Western blotting results showing MAT2A and MAT2B protein levels after *Acmsd* was over-expressed in U2OS cells. The assay was repeated twice with similar results.

(J) The total levels of NAD(H) decreased after NAMPT inhibitor (FK866) incubation and increased after NAMPT activator (P7C3) incubation. FK866 was added to a final concentration of 200 nM for 24 hours and P7C3 was added to a final concentration of 10 μM for 24 hours. The contents of individual compounds in DMSO treated U2OS cells, as determined by mass spectrometry, were normalized as 1. The data were represented by the mean ± SEM (N = 3 independent replicates). The *p* value was determined by two-sided unpaired t-test.

(K and L) MAT2A and MAT2B remained unchanged after the NAMPT inhibitor (K) and NAMPT activator (L) incubation in U2OS cells. FK866 was added to a final concentration of 200 nM for 24 hours and P7C3 was added to a final concentration of 10 μM for 24 hours. The assay was repeated twice with similar results.

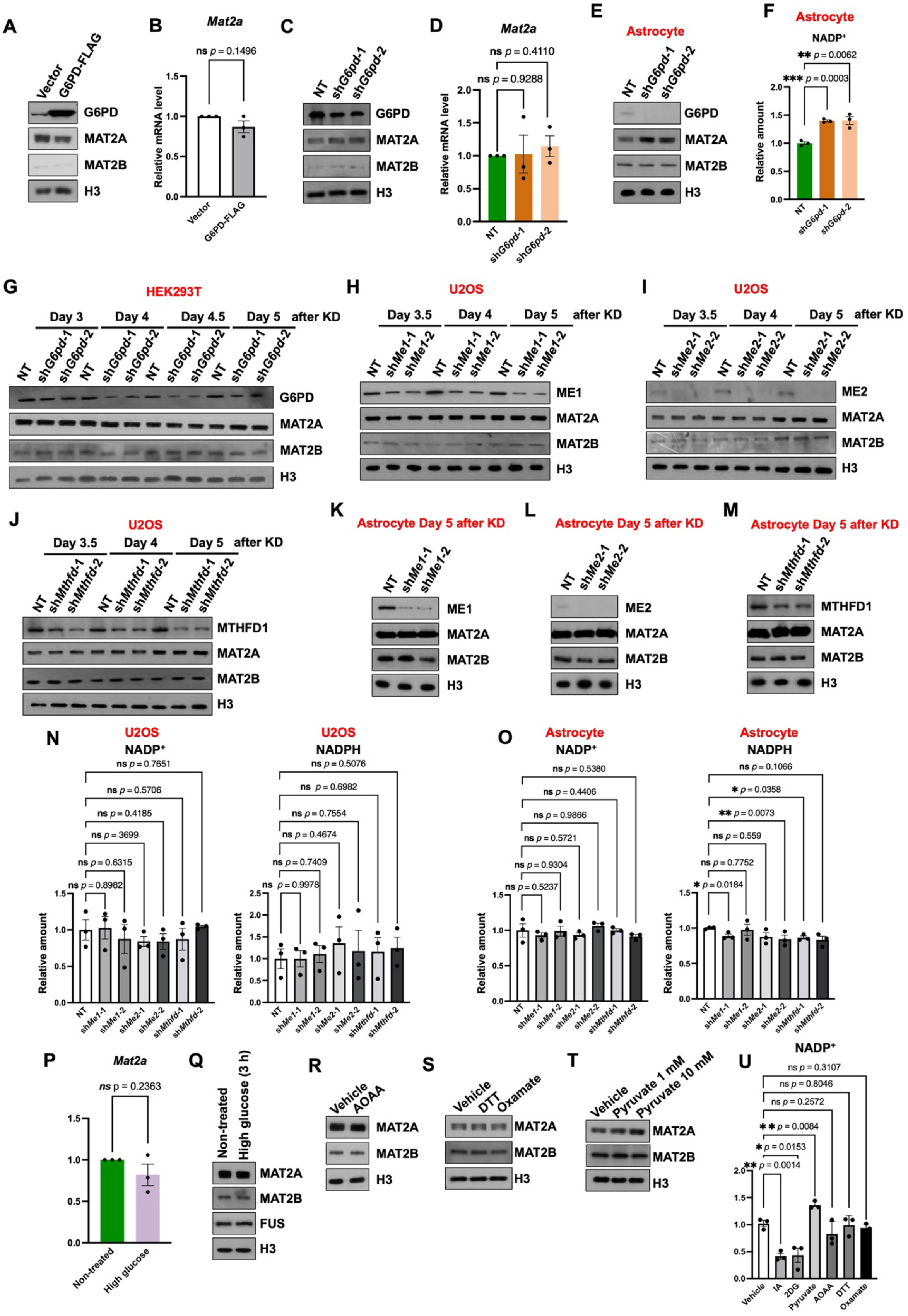

Supplementary Figure 3. PPP regulates NADP(H) and MAT2A levels.

(A) G6PD over-expression decreased MAT2A levels in U2OS cells. The assay was repeated three times with similar results.

(B) The gene expression of *Mat2a* remained unchanged after G6PD over-expression in U2OS cells. Gene expressions were normalized to *β-actin* and the expression levels in empty-vector transfected cells were further normalized as 1. The data were represented by the mean ± SEM (N = 3 independent replicates). The *p* value was determined by two-sided paired t-test.

(C) Western blotting showing MAT2A increased after G6PD depletion in U2OS cells. The assay was repeated four times with similar results.

(D) The gene expression of *Mat2a* remained unchanged after G6PD depletion in U2OS cells. *G6pd* was knocked down by two independent shRNAs. NT, non-target control. Gene expressions were normalized to *β-actin* and the expression levels in NT cells were further normalized as 1. The data were represented by the mean ± SEM (N = 3 independent replicates). The *p* value was determined by two-sided paired t-test.

(E) Same as (C), except astrocyte cells were used. The assay was repeated twice with similar results.

(F) Mass spectrometry results showing that the levels of $NADP^+$ increased with G6PD depletion in astrocyte cells. The contents of $NADP^+$ in NT cells were normalized as 1. The data were represented by the mean ± SEM (N = 3 independent replicates). The *p* value was determined by two-sided unpaired t-test.

(G) Western blotting results showing that MAT2A increased 5 days after G6PD depletion in HEK293T cells. The cells were collected at the indicated time points after *G6pd* was knocked down. The assay was repeated twice with similar results.

(H - J) Western blotting results showing that knocking down *Me1* (H), *Me2* (I), and *Mthfd1* (J) had no obvious effect on the levels of MAT2A in U2OS cells. The cells were collected at the indicated time points after depletion. These assays were repeated twice with similar results.

(K - M) Depletion of ME1 (K), ME2 (L), and MTHFD1 (M) showed no obvious effect on the levels of MAT2A in astrocyte cells. The cells were collected at the indicated time points after depletion. These assays were repeated twice with similar results.

(N and O) Mass spectrometry results showing the levels of NADP(H) after knocking down *Me1*, *Me2*, and *Mthfd1* in U2OS (N) and astrocyte cells (O). The contents of NADP(H) in NT cells were normalized as 1. The data were represented by the mean ± SEM (N = 3 independent replicates). The *p* value was determined by two-sided unpaired t-test.

(P) The expression of *Mat2a* remained unchanged after 40 g/L glucose incubation for 36 hours. Gene expressions were normalized to *β-actin* and the expression levels in non-treated U2OS

cells were further normalized as 1. The data were represented by the mean ± SEM (N = 3 independent replicates). The *p* value was determined by two-sided paired t-test.

(Q) High glucose (40 g/L) incubation for 3 hours did not alter protein levels of MAT2A in U2OS cells. The assay was repeated three times with similar results.

(R) Western blotting showing protein levels of MAT2A and MAT2B after incubation with 5 mM AOAA for 9 hours in U2OS cells. The assay was repeated three times with similar results.

(**S**) Western blotting showing protein levels of MAT2A and MAT2B after incubation with 200 μM DTT or 1 mM oxamate for 36 hours in U2OS cells. The assay was repeated twice with similar results.

(**T**) 10 mM pyruvate incubation for 48 hours increased the protein level of MAT2A in U2OS cells. The assay was repeated twice with similar results.

(**U**) The relative amounts of $NADP^+$ in cells treated with glycolysis inhibitors or accelerators. IA (10 μM for 24 h) and 2-DG (10 mM for 24 h) treatments decreased $NADP^+$ in U2OS cells. Pyruvate (10 mM for 24 h) increased the $NADP^+$ in U2OS cells. AOAA (5 mM for 9 h), DTT (200 μM for 36h), and oxamate (1 mM for 36 h) had no change in U2OS cells. The amounts of $NADP^+$ were detected by Coenzyme Ⅱ NADP(H) Content Assay Kit (Sangon Biotech, Cat. #D799249). The contents of $NDAP^+$ in cells treated with 0.1% DMSO were normalized as 1. The data were represented by the mean ± SEM (N = 3 independent replicates). The *p* value was determined by two-sided unpaired t-test.

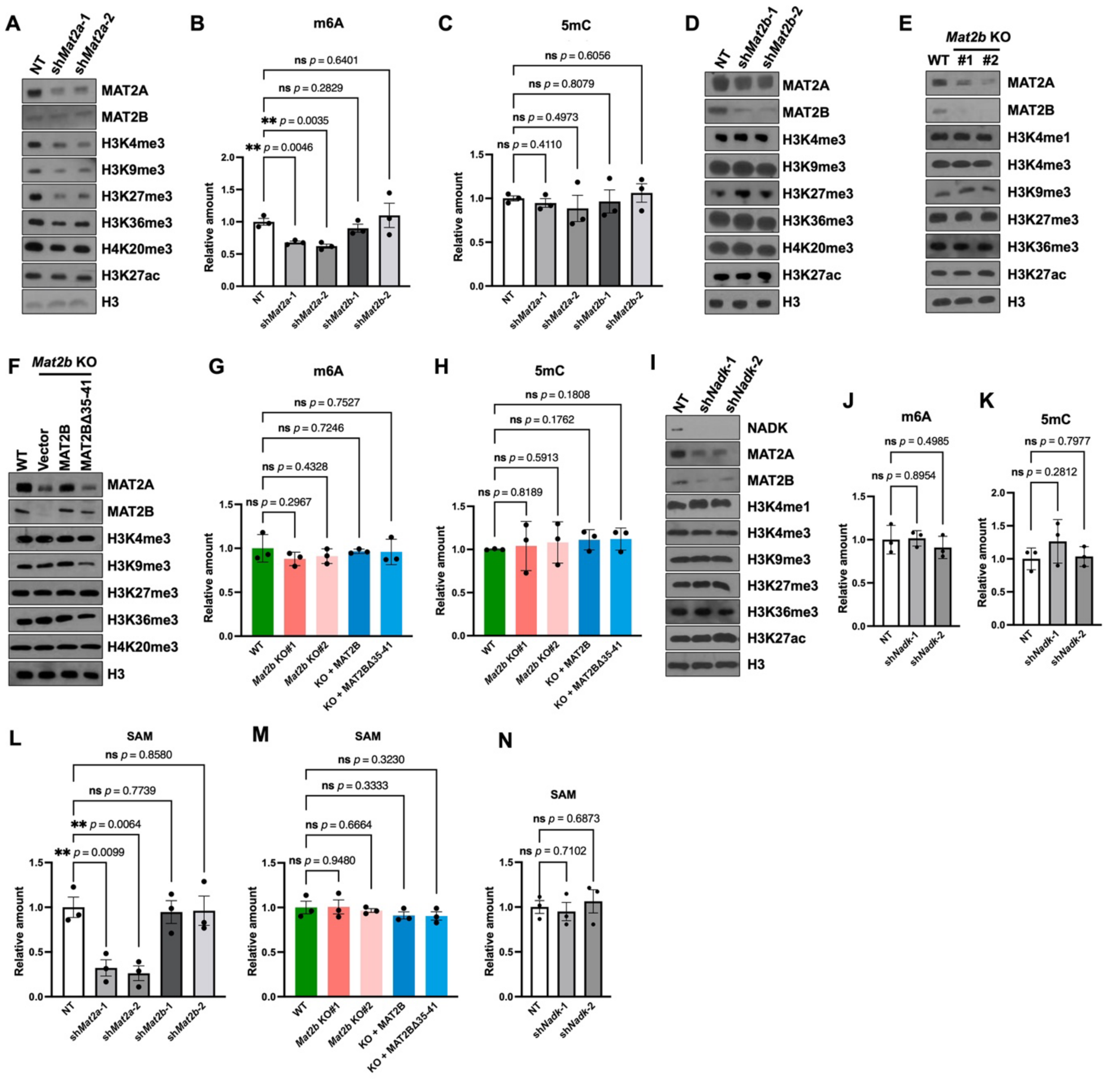

Supplementary Figure 4. MAT2B bind with MAT2A.

(A) Western blotting showing that knocking down of *Mat2a* decreased the levels of all tested histone methylation marks in U2OS cells. NT, non-target control. Two independent shRNAs were used to deplete MAT2A. The assay was repeated twice with similar results.

(B and C) Mass spectrometry results showing the contents of mRNA m6A (B) and DNA 5mC (C) after MAT2A or MAT2B depletion in U2OS cells. The contents of individual modifications in NT cells were normalized as 1. The data were represented by the mean ± SEM (N = 3 independent replicates). The *p* value was determined by two-sided unpaired t-test.

(D) Depletion of MAT2B showed little effect on the total levels of tested histone methylations. The assay was repeated at least three times with similar results.

(E and F) Western blotting showing that *Mat2b* KO (E) and re-expression of WT MAT2B or MAT2BΔ35-41 (F) did not alter the levels of tested histone methylations in U2OS cells. The assays were repeated twice with similar results respectively.

(G and H) Same as in (B) and (C), except *Mat2b* KO and re-expressed cells were used.

(I) Depletion of NADK showed little effect on the total levels of tested histone methylations. The assay was repeated three times with similar results.

(J and K) Same as in (B) and (C), except *Nadk* was knocked down.

(L) Mass spectrometry results showing the contents of SAM after MAT2A or MAT2B depletion in U2OS cells. The contents of SAM in NT (non-target) cells were normalized as 1. The data were represented by the mean ± SEM (N = 3 independent replicates). The *p* value was determined by two-sided unpaired t-test.

(M) Same as in (L), except *Mat2b* KO and re-expressed cells were used.

(N) Same as in (L), except NADK depleted cells were used.

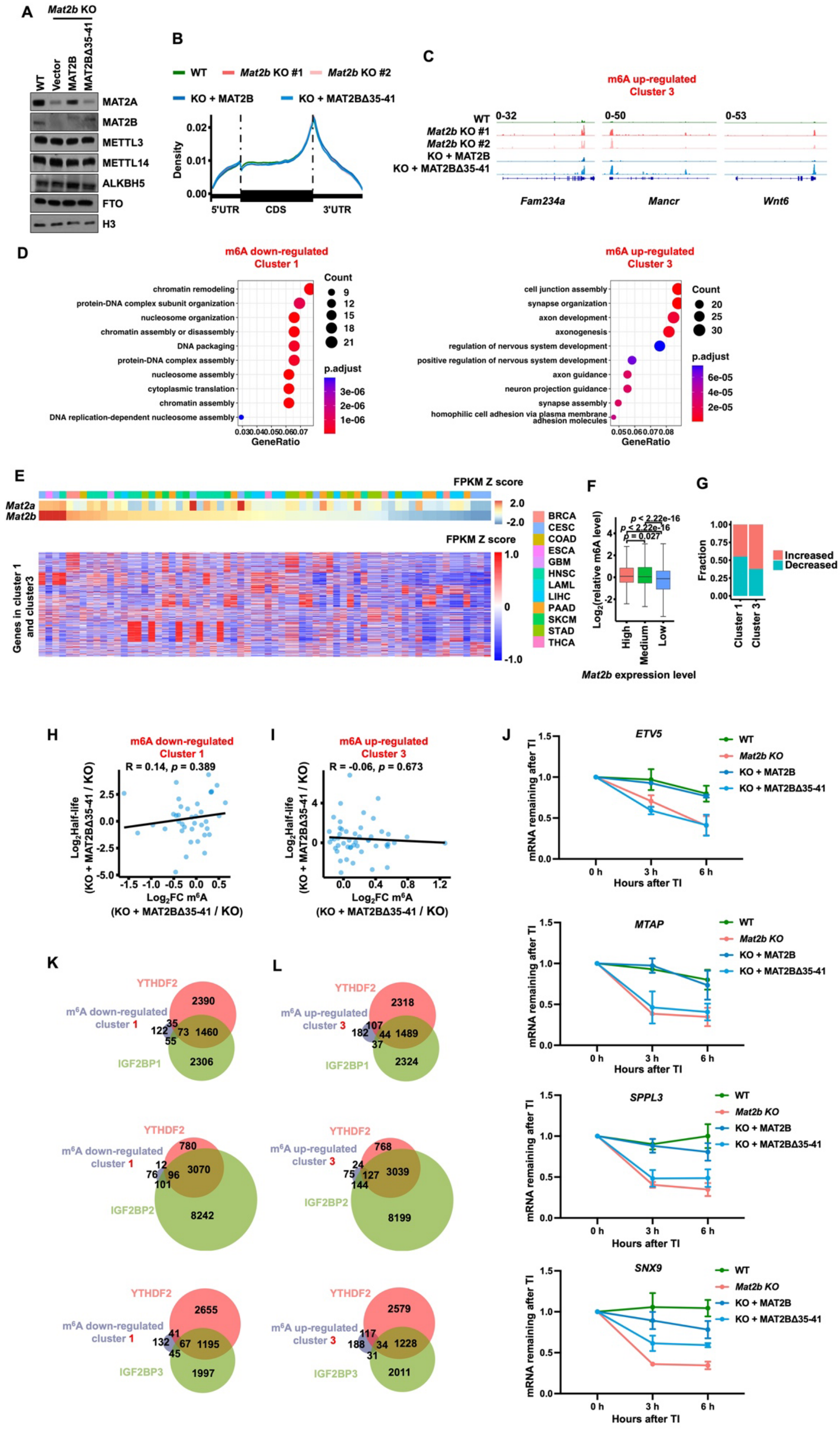

Supplementary Figure 5. NADP⁺ controls the m6A modification and stability of mRNA.

(A) Western blotting results representing the total levels of indicated proteins in *Mat2b* KO and MAT2B re-expressed U2OS cells. Cell extracts were analyzed by Western blotting using the indicated antibodies. The assay was repeated three times with similar results.

(B) The reads density distribution profiles of m6A signals spanning gene bodies in WT, *Mat2b* KO, WT MAT2B rescued, and MAT2BΔ35-41 rescued U2OS cells. The average read density at all genes identified by NCBI RefSeq was plotted. UTR, untranslated regions. CDS, coding sequence.

(C) IGV tracks presenting the enrichment of m6A methylation in m6A up-regulated Cluster 3 genes.

(D) GO terms showing the genes in m6A down-regulated Cluster 1 or m6A up-regulated Cluster 3.

(E) The heatmaps showing the expression levels of *Mat2a* and *Mat2b* (upper panel) and m6a levels of genes in m6A down-regulated Cluster 1 and m6A up-regulated Cluster 3 (lower panel) across cancers. The samples were sorted in descending order based on *Mat2b* expression levels. RNA-Seq and MeRIP-Seq data were sourced from the NCBI GeneExpression Omnibus (GEO) database.

(F) The boxplots showing the relative abundance of m6A in high, medium, and low groups. The groups were divided based on *Mat2b* expression levels from high to low as shown in (E). The relative abundance was calculated as the ratio of FPKM to the median value for each gene. FPKM, fragments per kilobase per million mapped reads. The p values were calculated by two-sided paired t-test.

(G) The bar plots showing the fractions of genes with increased and decreased half-life in m6A down-regulated Cluster 1 and m6A up-regulated Cluster 3.

(H and I) Correlations between the altered signals of half-life and m6A methylation in MAT2BΔ35-41 re-expressed U2OS cells compared with *Mat2b* KO U2OS cells. Genes in m6A down-regulated Cluster 1 genes (H) and m6A up-regulated Cluster 3 genes (I) were shown. R, correlation coefficients that were assessed by Pearson product-moment correlation. The p values were calculated by two-sided paired t-test.

(J) mRNA stability of selected top genes in m6A down-regulated Cluster 1 in WT, *Mat2b* KO, WT MAT2B rescued and MAT2BΔ35-41 rescued U2OS cells. TI denotes transcription inhibition. Transcription was inhibited by actinomycin D (10 μg/ml). Gene expressions at 0h were normalized as 1. The data were represented by the mean ± SD (N = 3 independent replicates).

(K and L) Venn diagram showing the overlap of genes in m6A down-regulated Cluster 1 (K) or m6A up-regulated Cluster 3 (L) with m6A readers YTHDF2 and IGF2BP1/2/3 binding genes.

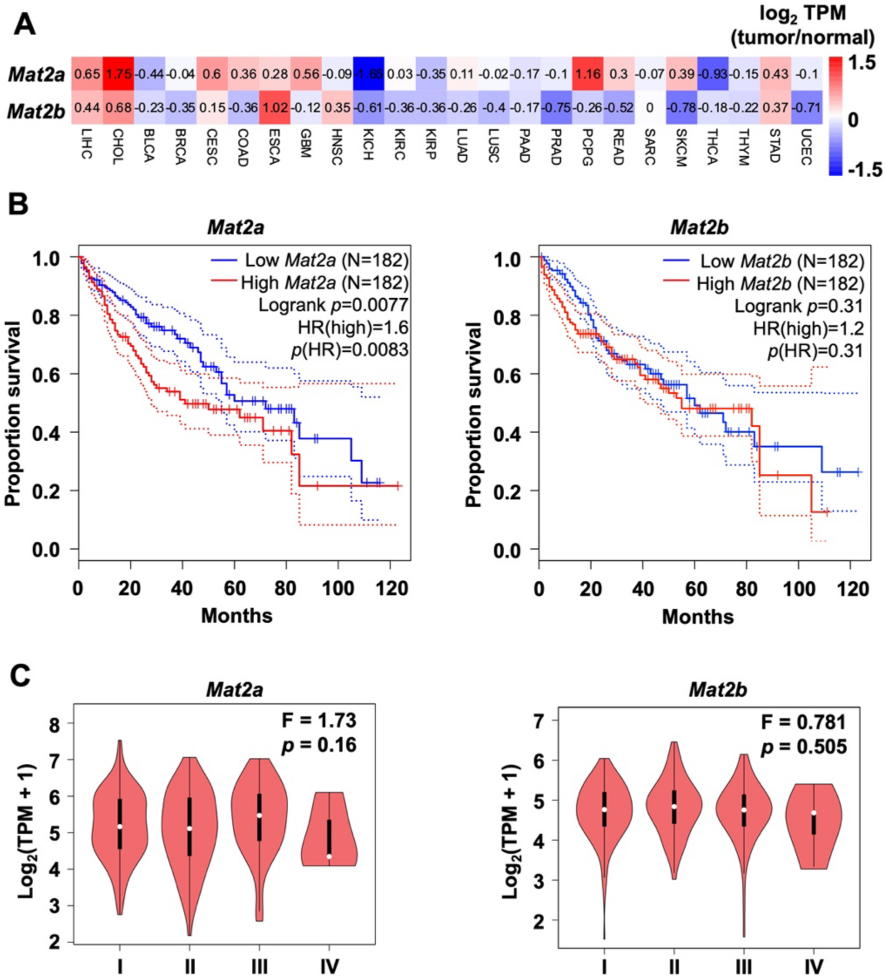

Supplementary Figure 6. MAT2A and MAT2B are associated with liver tumor formation.

(A) The heatmap showing the expression changes of *Mat2a* and *Mat2b* across TCGA cancers. RNA-Seq data were sourced from the TCGA database through UALCAN analysis (ref. 1). TPM, transcript per million.

(B) Kaplan–Meier curves of the overall survival in LIHC cases based on *Mat2a* and *Mat2b* levels. Patient data were sourced from the TCGA database through GEPIA analysis (ref. 2). HR, hazard ratio.

(C) Pathological stage plot showing *Mat2a* and *Mat2b* expression levels in LIHC cases. RNA-Seq data were sourced from the TCGA database through GEPIA analysis (ref. 2). The p values were calculated by one-way ANOVA. TPM, transcript per million.

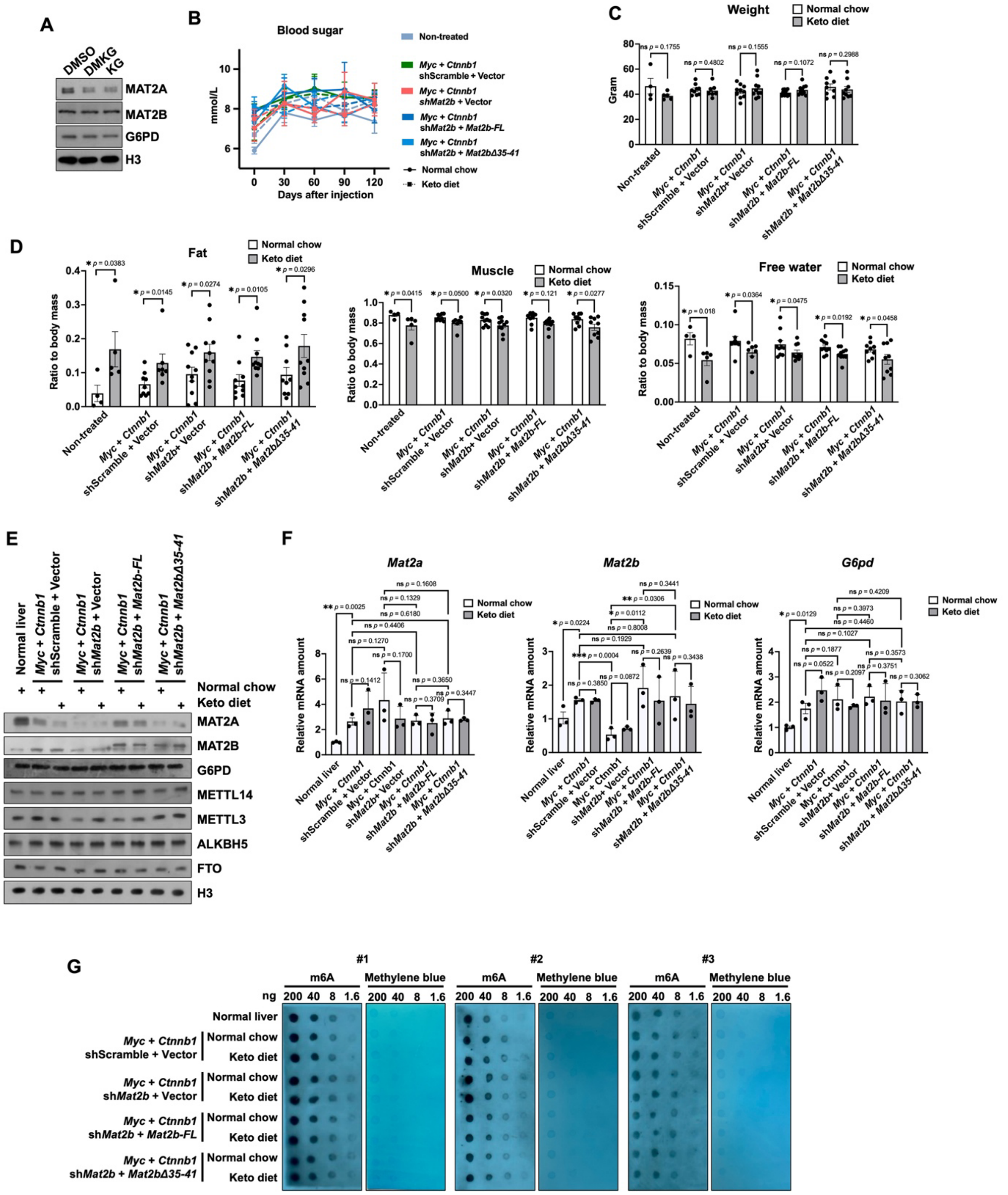

Supplementary Figure 7. NADP$^+$ participates in the tumorigenesis of liver tumors.

(A) Western blotting results showing MAT2A decreased when U2OS cells were treated with KG or DMKG.

(B) The concentrations of blood sugar in mice at intervals of 0, 30, 60, 90, and 120 days after injection. 3 mice were analyzed at each time point. Normal chow, the control normal diet. The mice were fed different diets upon injection.

(C) The weights of mice in different cohorts. 5-10 mice in each group were summarized. Data are mean ± SEM. The *p* values were determined by paired t-test, one-sided.
(D) The contents of fat, muscle, and free water of mice in different cohorts. 4-10 mice in each group were summarized. Data are mean ± SEM. The *p* values were determined by paired t-test, one-sided.
(E) Western blotting showing the indicated protein levels in different cohorts. Tissues were ground with steel balls and boiled with SDS loading buffer.
(F) The expressions of *Mat2a, Mat2b,* and *G6pd* in different cohorts. Gene expressions were normalized to *β-actin* and the expression levels in normal liver were further normalized as 1. The data were represented by the mean ± SEM (N = 3 independent replicates). The *p* values were determined by two-sided paired t-test.
(G) Dot blot data showing mRNA m6A levels in different cohorts. The left patterns were the signals of mRNA m6A levels stained with m6A antibody, and the right patterns were the relative amount of mRNA stained by methylene blue. Three independent repeats were present.

Supplementary Table S1. Correlations between two replicates of sequencing results. A 1 kb sliding window across the whole genome was used to calculate the Pearson product moment correlation for epigenomic sequencing.

Sample	Correlation
WT IP MeRIP-seq	0.94
WT Input MeRIP-seq	0.99
Mat2b KO #1 IP MeRIP-seq	0.99
Mat2b KO #1 Input MeRIP-seq	0.91
Mat2b KO #2 IP MeRIP-seq	0.93
Mat2b KO #2 Input MeRIP-seq	0.91
WT MAT2B rescue IP MeRIP-seq	0.94
WT MAT2B rescue Input MeRIP-seq	0.95
MAT2BΔ35-41 rescue IP MeRIP-seq	0.99
MAT2BΔ35-41 rescue Input MeRIP-seq	0.91
NT IP MeRIP-seq	0.97
NT Input MeRIP-seq	0.98
sh*G6pd* #1 IP MeRIP-seq	1.00
sh*G6pd* #1 Input MeRIP-seq	0.99
sh*G6pd* #2 IP MeRIP-seq	0.99
sh*G6pd* #2 Input MeRIP-seq	0.99
non-treated IP MeRIP-seq	0.98
non-treated Input MeRIP-seq	0.81
high glucose IP MeRIP-seq	0.99
high glucose Input MeRIP-seq	0.98

Supplementary Table S2. Oligonucleotides used in this paper

Name	5' - 3'	Source	Application
*Mat2b*_sgRNA_1	CTCTCTATACACTTTGTTCC	human	Knock-out
*Mat2b*_sgRNA_2	GGGAGCTGTCGGCTGGTGGA	human	Knock-out
*Mat2a*_shRNA_1	GTTCAGGTCTCTTATGCTATT	human	Knock-down
*Mat2a*_shRNA_2	AGTACTTGTAGTTCCACTTAT	human	Knock-down
*Mat2b*_shRNA_1	CGGGAGAAAGAGCTCTCTATA	human	Knock-down
*Mat2b*_shRNA_2	GAGCTCTCTATACACTTTGTT	human	Knock-down
*Nadk*_shRNA_1	CGATGAGACCTGGAGTTACAA	human	Knock-down
*Nadk*_shRNA_2	AGGAGAACATGATCGTGTATG	human	Knock-down
*Nadk2*_shRNA_1	GCTGTAGCAGTGGACAATTTA	human	Knock-down
*Nadk2*_shRNA_2	GAACGGTCTGAGGGTCATTTA	human	Knock-down
*G6pd*_shRNA_1	CAACAGATACAAGAACGTGAA	human	Knock-down
*G6pd*_shRNA_2	GTCGTCCTCTATGTGGAGAAT	human	Knock-down
*6pgd*_shRNA_1	TCTTACGCTCAAGGCTTTATG	human	Knock-down

**Name**	**5' - 3'**	**Source**	**Application**
*6pgd*_shRNA_2	CTGTCTTTGCTCGGTGCTTAT	human	Knock-down
*Mthfd1*shRNA_1	GCCATTGATGCTCGGATATTT	human	Knock-down
*Mthfd1*_shRNA_2	GCAGATGACATTGAATTACTT	human	Knock-down
*Me1*_shRNA_1	GCTGAGGTTATAGCTCAGCAA	human	Knock-down
*Me1*_shRNA_2	CCTGTGGGTAAATTGGCTCTA	human	Knock-down
*Me2*_shRNA_1	GCACGGCTGAAGAAGCATATA	human	Knock-down
*Me2*_shRNA_2	AGTTCTTACAGAGCTACTAAA	human	Knock-down
*Acmsd1*_shRNA_1	GGGATCCAGAAGTTCGTATTA	human	Knock-down
*Acmsd1*_shRNA_2	ATGATCATGGGTGGAGTATTT	human	Knock-down
*Acmsd1*_shRNA_3	GATGTCATAGGAAAGGATAAA	human	Knock-down
*Acmsd1*_shRNA_4	CAGAGACCACCATAGCCATTT	human	Knock-down
*Acmsd1*_shRNA_5	ACTACAAAGGCAAACTTTCAA	human	Knock-down
Mat2b_shRNA_1	GCAGCCACTTAAGACCTATTA	mouse	Knock-down
Mat2b_shRNA_2	GCAGATGACCAAGTATGAAAT	mouse	Knock-down
β-actin_F	CATGTACGTTGCTATCCAGGC	human	qPCR
β-actin_R	CTCCTTAATGTCACGCACGAT	human	qPCR
Mat2a_F	ATGAACGGACAGCTCAACGG	human	qPCR
Mat2a_R	CCAGCAAGAAGGATCATTCCAG	human	qPCR
Mat2b_F	TTCACTGGTCTGGCAATGAAC	human	qPCR
Mat2b_R	AGGGCTGTCAGTAATAGGTCTT	human	qPCR
Nadk_F	ACCTGAAGCAAGGAACACAGC	human	qPCR
Nadk_R	AGCGGGTAGCATGAGGTAGT	human	qPCR
6pgd_F	TCATGCGGCTGAATTCCCTT	human	qPCR
6pgd_R	TGGCCGAGGTGAGACTAAGA	human	qPCR
Acmsd_F	GGTGCGAGAGAATTGCTGG	human	qPCR
Acmsd_R	TGCTGGCAAGGTCGTTGTTT	human	qPCR
Etv5_F	TCAGCAAGTCCCTTTTATGGTC	human	qPCR
Etv5_R	GCTCTTCAGAATCGTGAGCCA	human	qPCR
Sppl3_F	CAGACCTACTCGTGGGCCTAT	human	qPCR
Sppl3_R	ACAGAGCCTGGGTAGAGTCAA	human	qPCR
Mtap_F	ACCACCGCCGTGAAGATTG	human	qPCR
Mtap_R	GCATCAGATGGCTTGCCAA	human	qPCR
Snx9_F	ACCAAGGCTCGGGTTATGTAT	human	qPCR
Snx9_R	CCCTCGTTCTCCTTTGATGTTT	human	qPCR
Hsd17b3_F	CTGGCGAAGTGCGTGAGATT	human	qPCR
Hsd17b3_R	GAGTACGCTTTCCCAATTCCAT	human	qPCR
Commd4_F	TGATCTGGACTGTCCCGACTG	human	qPCR
Commd4_R	AGCCGCAACTTCACAGAGG	human	qPCR

Name	5' - 3'	Source	Application
Rab21_F	CGAGCCTACTCGTTCAAGGTG	human	qPCR
Rab21_R	TGTCGTTAAACTTGTTCTCGCA	human	qPCR
Dffa_F	GGACCTCCAGATGCTTGTTGA	human	qPCR
Dffa_R	GGAGCTGCTTGGACTGACG	human	qPCR
β-actin_F	GTGACGTTGACATCCGTAAAGA	mouse	qPCR
β-actin_R	GCCGGACTCATCGTACTCC	mouse	qPCR
Mat2a_F	GCTTCCACGAGGCGTTCAT	mouse	qPCR
Mat2a_R	CATCAGGGTCTTGTTGAAGGTG	mouse	qPCR
Mat2b_F	GGCGGGTTCTCATTACTGGTG	mouse	qPCR
Mat2b_R	GCTTCAGAATCCAACAGGTTCA	mouse	qPCR
G6pdx_F	CACAGTGGACGACATCCGAAA	mouse	qPCR
G6pdx_R	GCAGGGCATTCATGTGGCT	mouse	qPCR
G6pd2_F	AGGTGACCCTAAGCCGGAC	mouse	qPCR
G6pd2_R	TCTTTGGGTAGAAGACCATCCC	mouse	qPCR

Reference

1. Chandrashekar DS, Bashel B, Balasubramanya SAH, Creighton CJ, Ponce-Rodriguez I, Chakravarthi B*, et al.* UALCAN: A Portal for Facilitating Tumor Subgroup Gene Expression and Survival Analyses. *Neoplasia* 2017, **19**(8)**:** 649-658.
2. Tang Z, Li C, Kang B, Gao G, Li C, Zhang Z. GEPIA: a web server for cancer and normal gene expression profiling and interactive analyses. *Nucleic Acids Res* 2017, **45**(W1)**:** W98-W102.
