# Supplementary material for: MAT2B regulates the protein level of MAT2A to preserve RNA N6-methyladenosine
Source: Cell Death Dis. 2024 Oct 1;15(10):714. doi: 10.1038/s41419-024-07093-8 (PMC11445541; doi:10.1038/s41419-024-07093-8)
Supplement: Supplementary file 2 — Raw WB Data [file 41419_2024_7093_MOESM2_ESM.pdf]

Figure 1

A

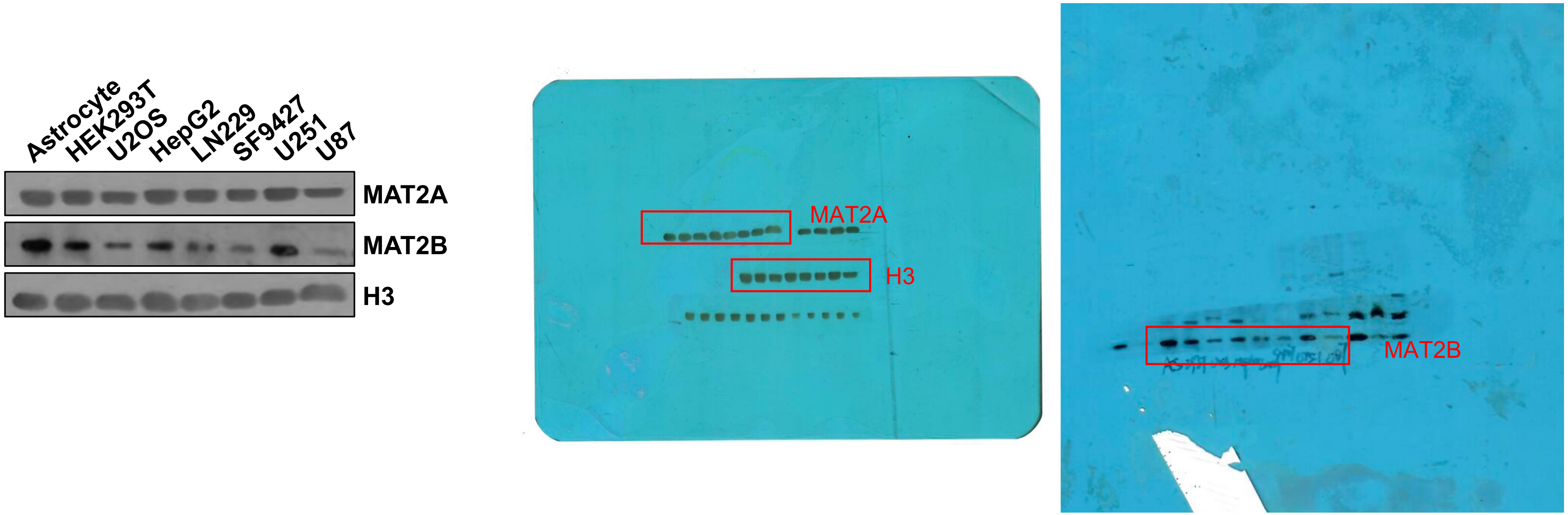
Astrocyte
HEK293T
U2OS
HepG2
LN229
SF9427
U251
U87
MAT2A
MAT2B
H3
MAT2A
H3
MAT2B

Figure 1

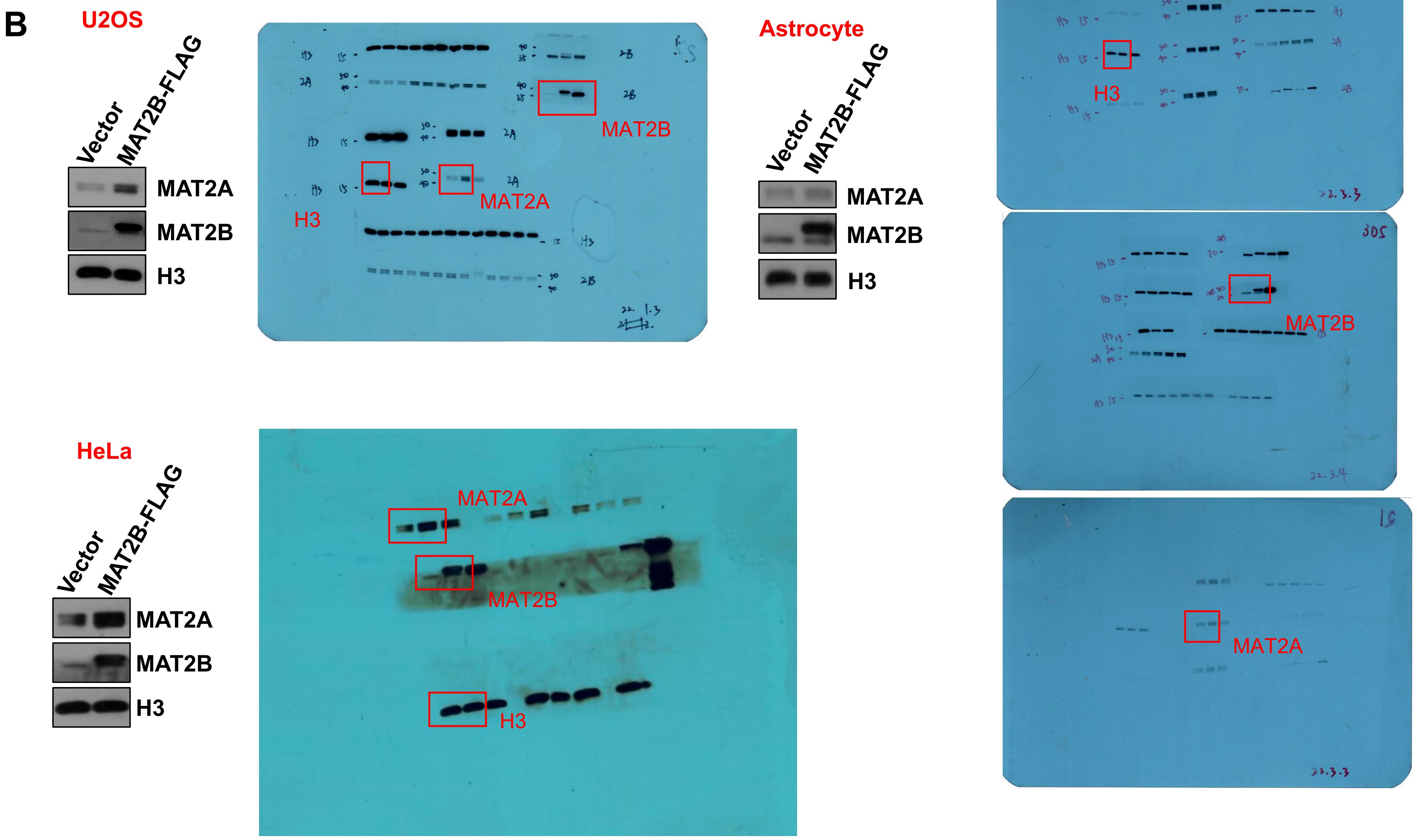
B
U2OS
Vector
MAT2B-FLAG
MAT2A
MAT2B
H3
MAT2B
MAT2A
H3
Astrocyte
Vector
MAT2B-FLAG
MAT2A
MAT2B
H3
H3
MAT2B
MAT2A
HeLa
Vector
MAT2B-FLAG
MAT2A
MAT2B
H3
MAT2A
MAT2B
H3

Figure 1

C

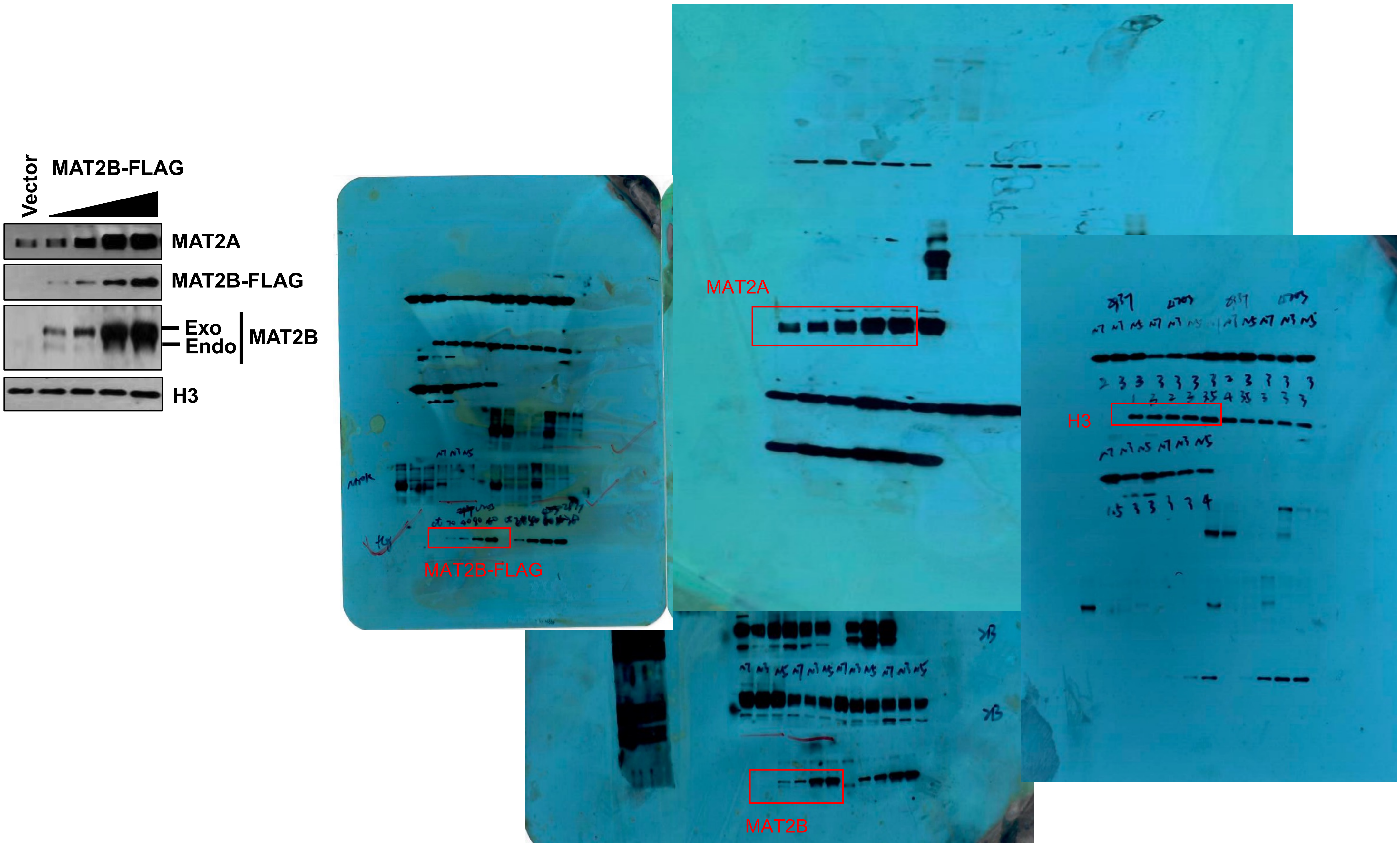

Vector
MAT2B-FLAG
MAT2A
MAT2B-FLAG
Exo
Endo
MAT2B
H3
MAT2B-FLAG
MAT2A
MAT2B
H3

Figure 1

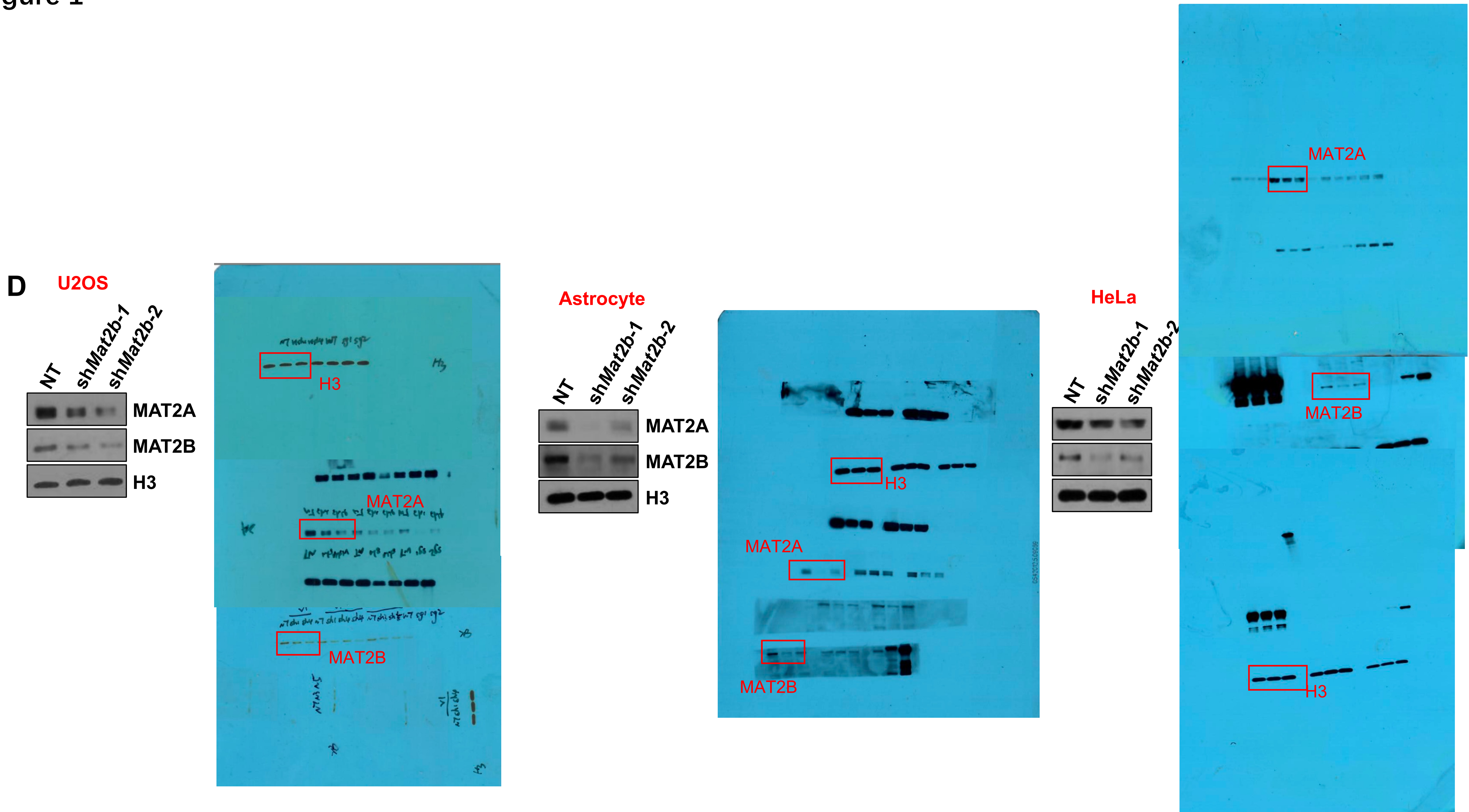
D
U2OS
NT
shMat2b-1
shMat2b-2
MAT2A
MAT2B
H3
H3
MAT2A
MAT2B
Astrocyte
NT
shMat2b-1
shMat2b-2
MAT2A
MAT2B
H3
H3
MAT2A
MAT2B
HeLa
NT
shMat2b-1
shMat2b-2
MAT2A
MAT2B
H3

Figure 1

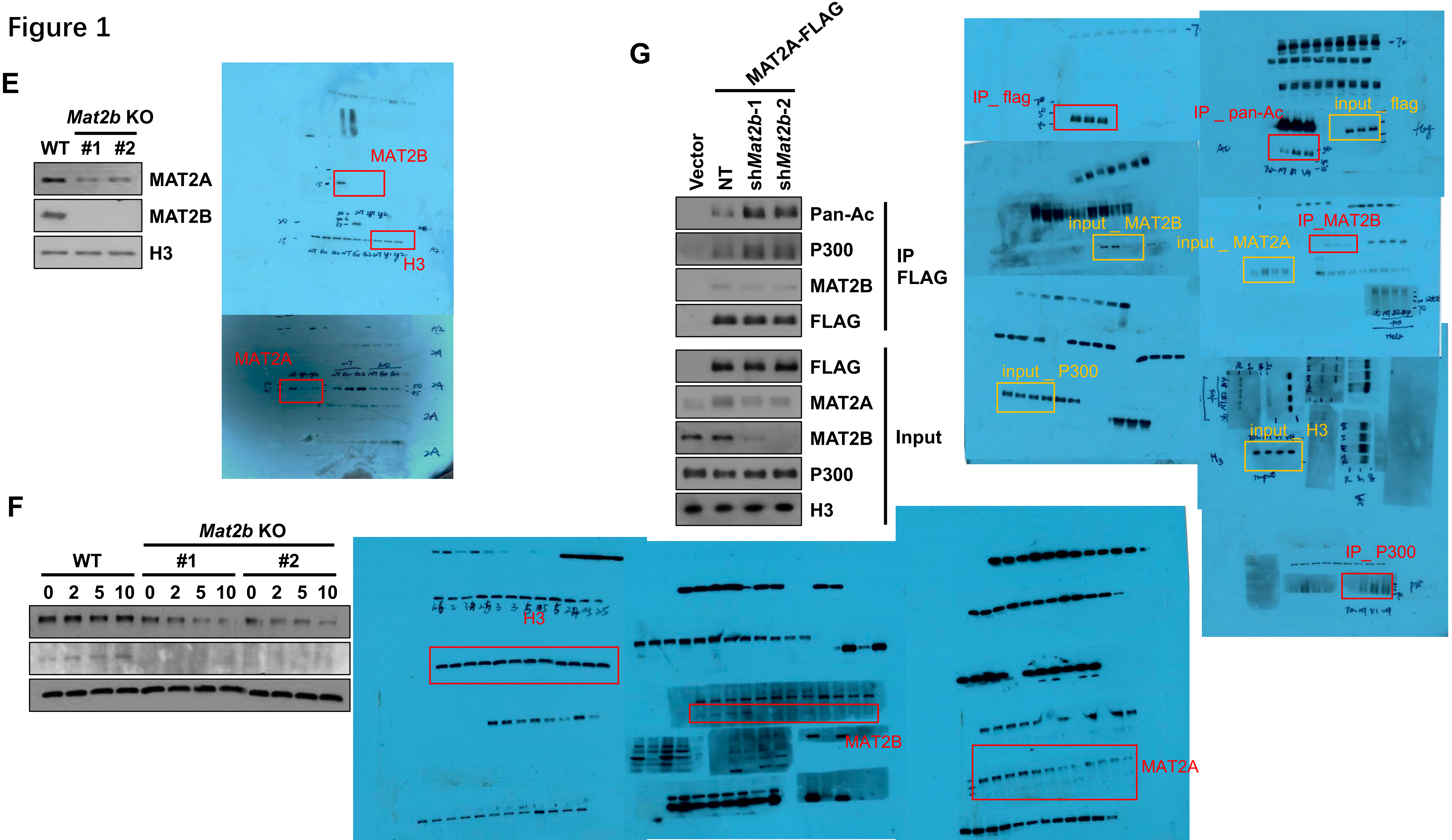

E
Mat2b KO
WT
#1
#2
MAT2A
MAT2B
H3
MAT2B
H3
MAT2A
F
Mat2b KO
WT
#1
#2
0
2
5
10
H3
MAT2B
MAT2A
G
MAT2A-FLAG
Vector
NT
shMat2b-1
shMat2b-2
Pan-Ac
P300
MAT2B
FLAG
IP FLAG
FLAG
MAT2A
MAT2B
P300
H3
Input
IP_ flag
IP _ pan-Ac
input _ flag
input _ MAT2B
input _ MAT2A
IP_MAT2B
input _ P300
input _ H3
IP_ P300

Figure 1

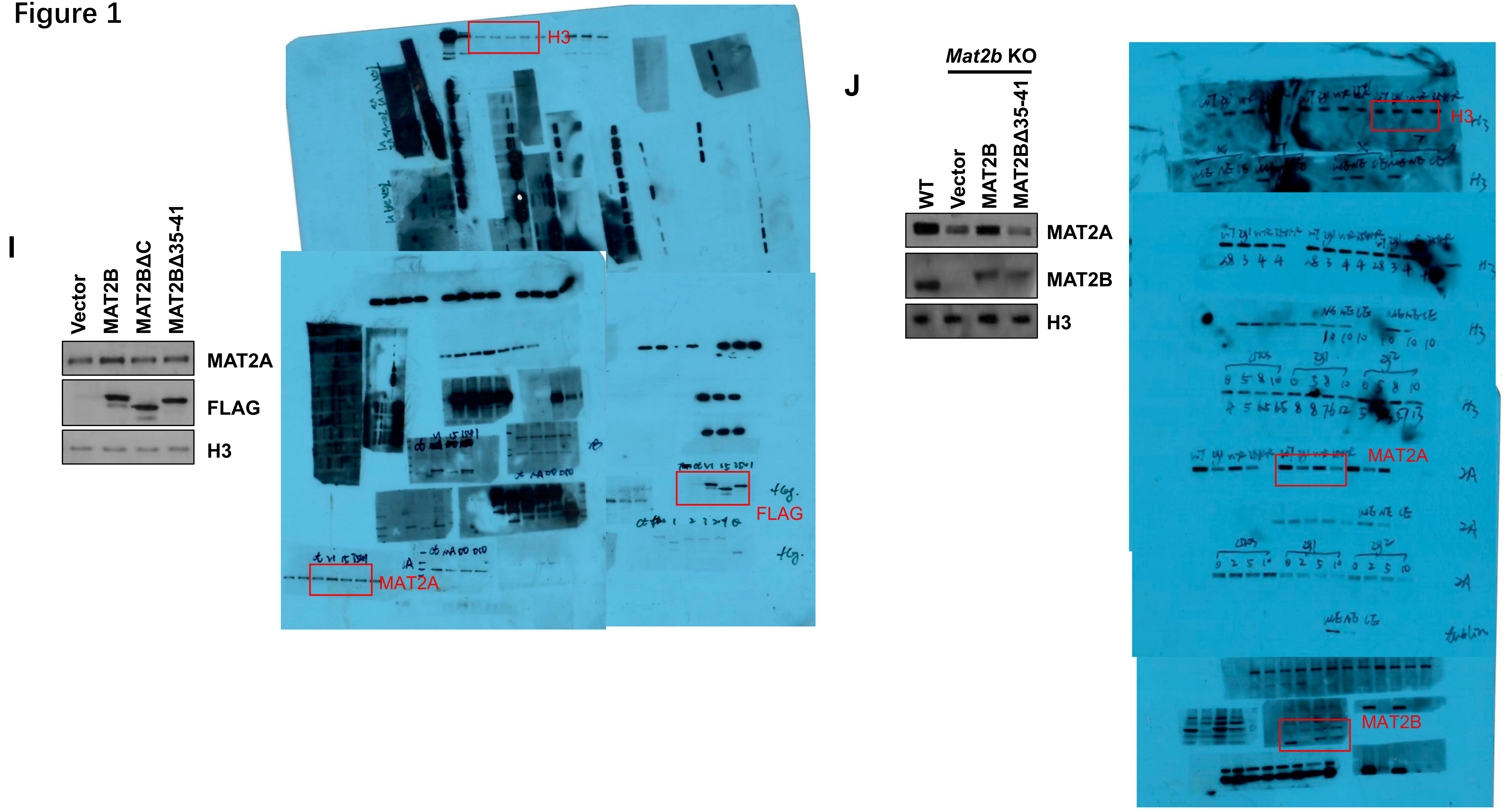
I
Vector
MAT2B
MAT2BΔC
MAT2BΔ35-41
MAT2A
FLAG
H3
J
Mat2b KO
WT
Vector
MAT2B
MAT2BΔ35-41
MAT2A
MAT2B
H3

Figure 1

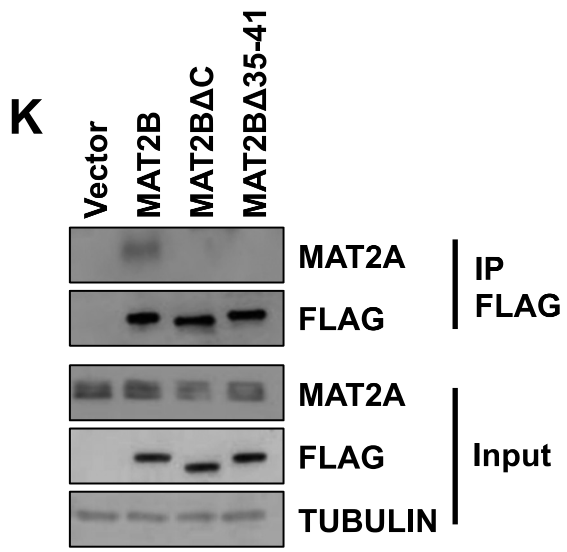
K
Vector
MAT2B
MAT2BΔC
MAT2BΔ35-41
MAT2A
FLAG
IP FLAG
MAT2A
FLAG
TUBULIN
Input

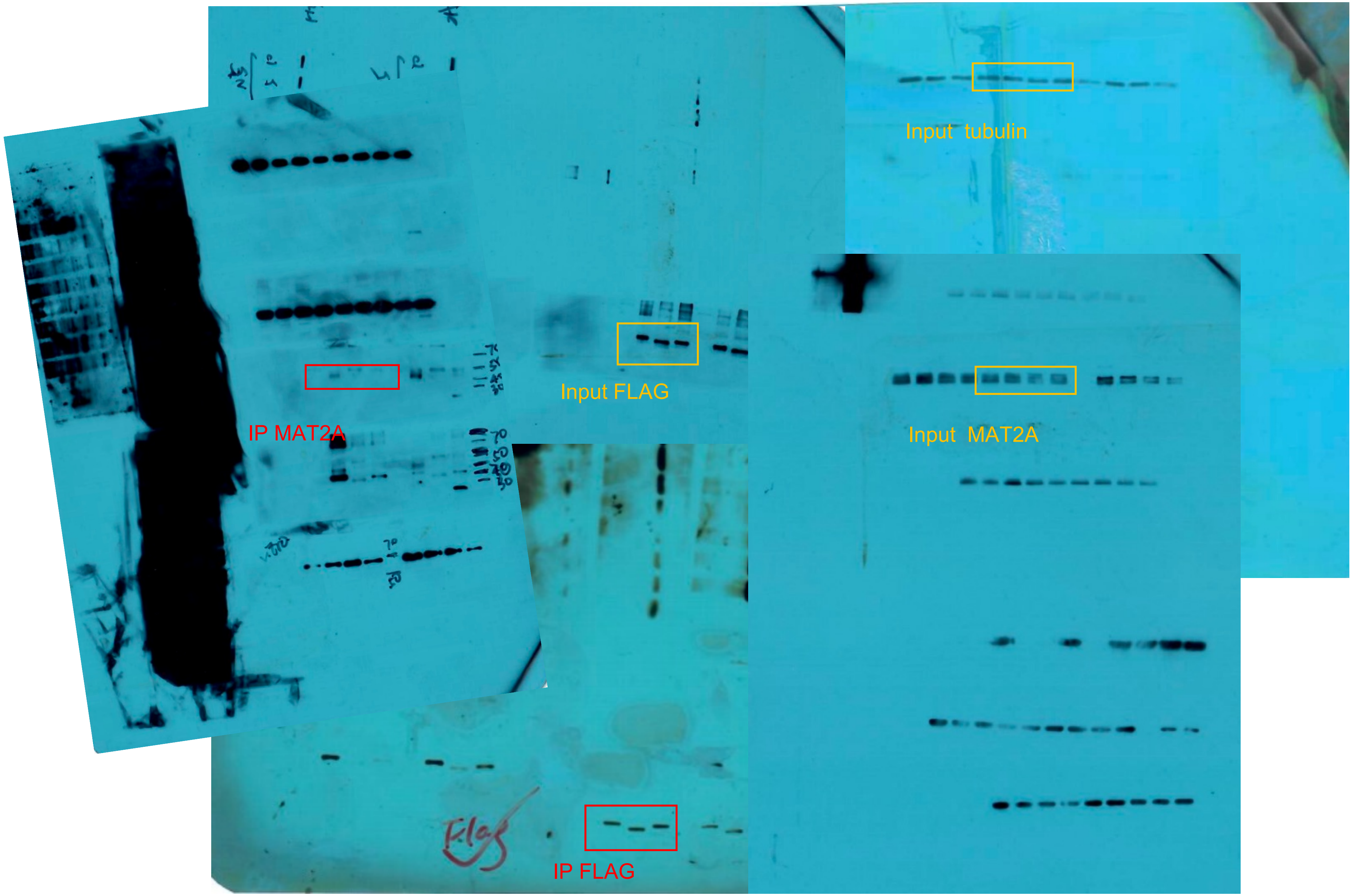
Input tubulin
Input FLAG
IP MAT2A
Input MAT2A
IP FLAG
Flag

Figure 1

L

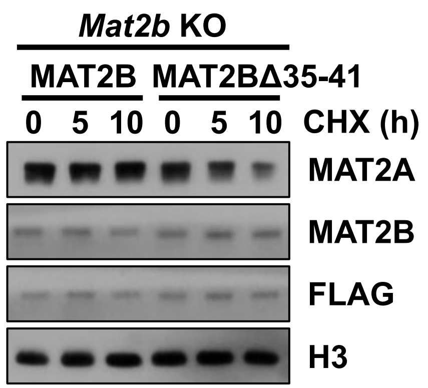
Mat2b KO
MAT2B
MAT2BΔ35-41
0 5 10 0 5 10 CHX (h)
MAT2A
MAT2B
FLAG
H3

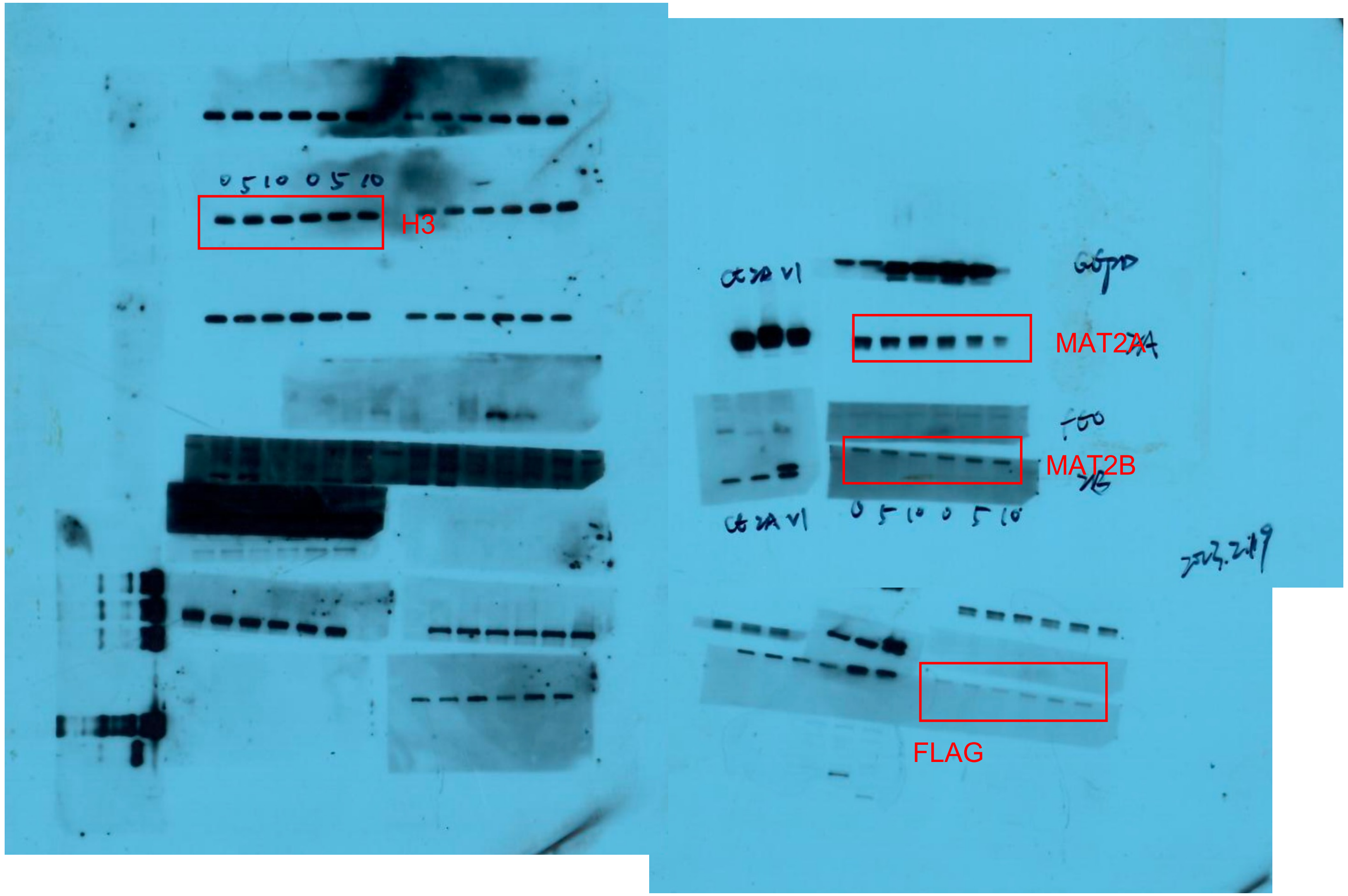
H3
MAT2A
MAT2B
FLAG

Figure S1

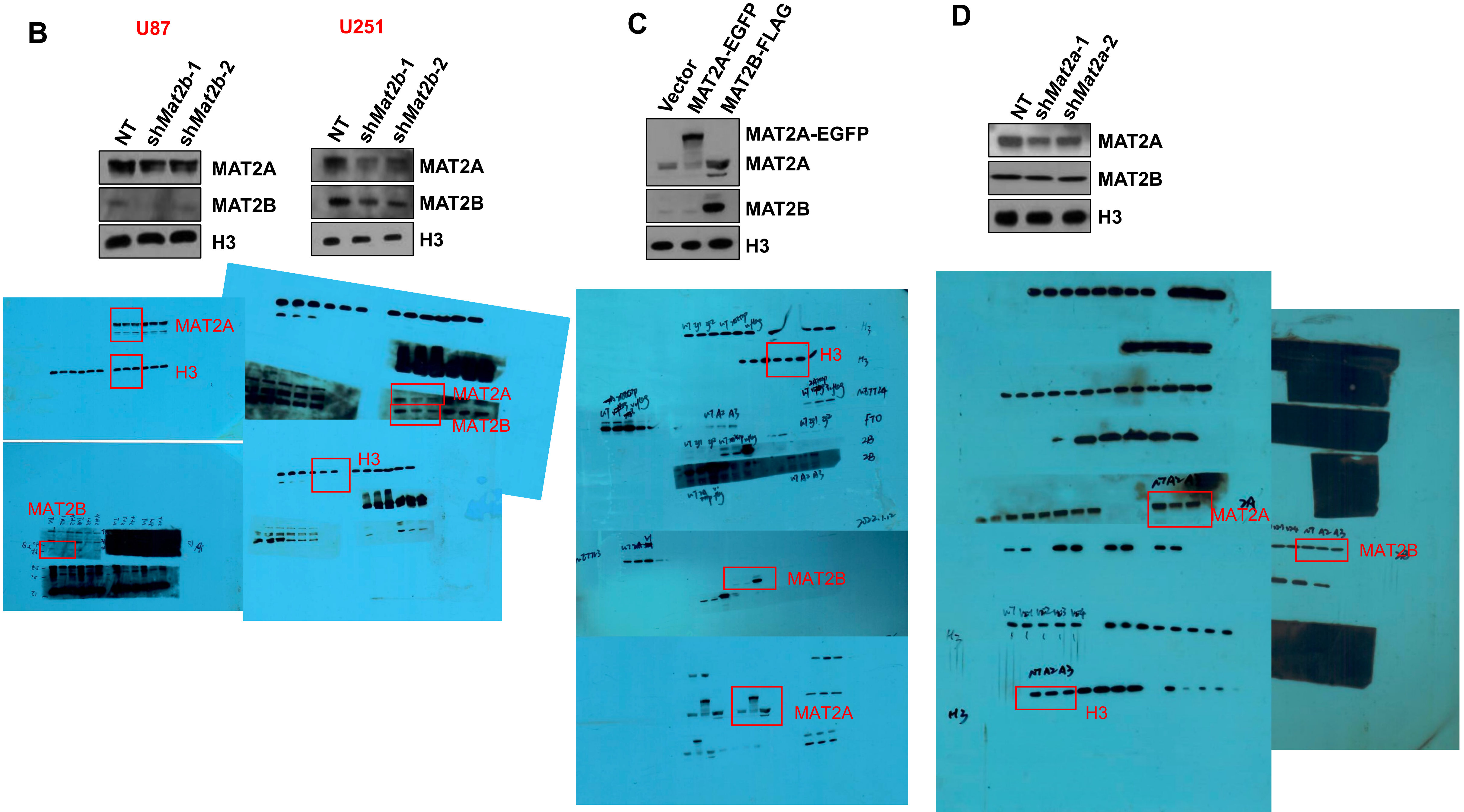
B
U87
U251
NT
shMat2b-1
shMat2b-2
MAT2A
MAT2B
H3
C
Vector
MAT2A-EGFP
MAT2B-FLAG
MAT2A-EGFP
MAT2A
MAT2B
H3
D
NT
shMat2a-1
shMat2a-2
MAT2A
MAT2B
H3

Figure S1

I

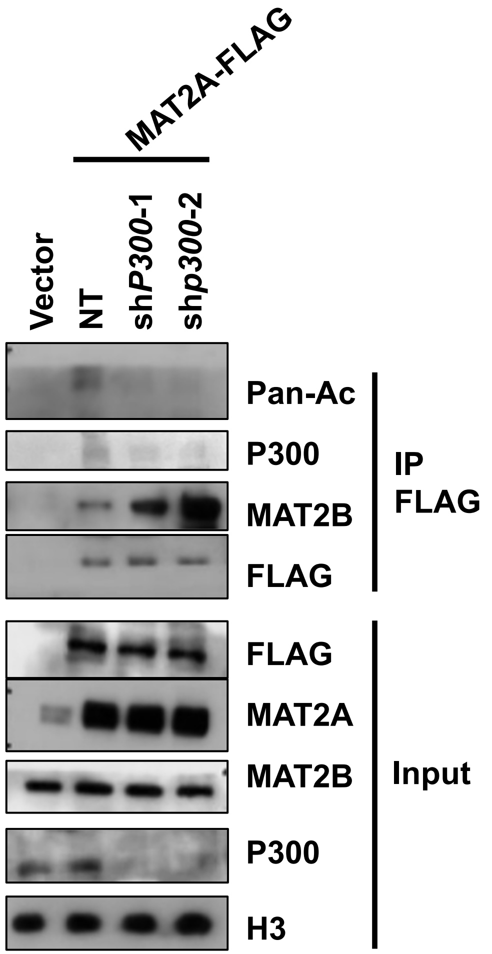

MAT2A-FLAG
Vector
NT
shP300-1
shp300-2
Pan-Ac
P300
MAT2B
FLAG
IP FLAG
FLAG
MAT2A
MAT2B
P300
H3
Input

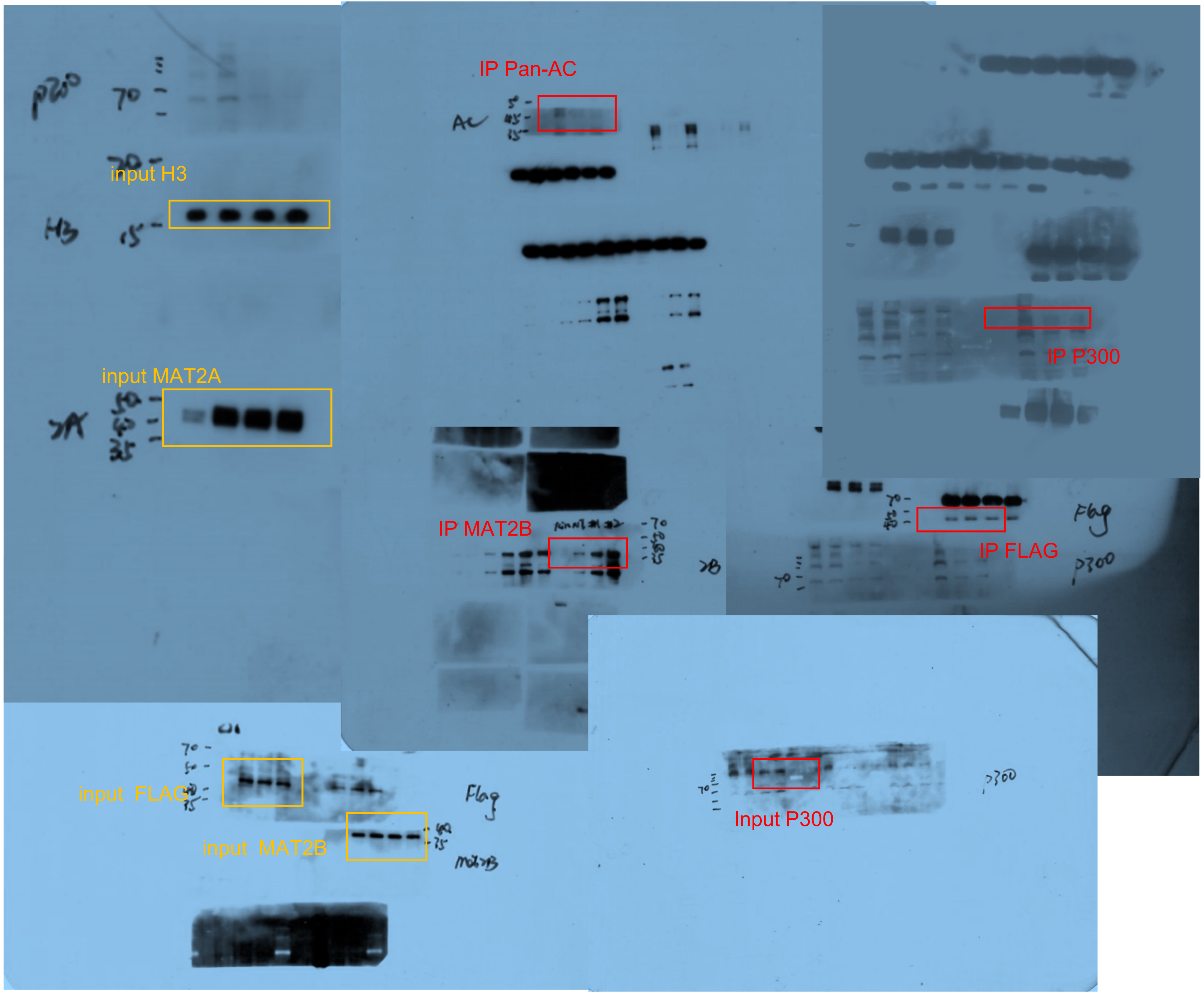

IP Pan-AC
input H3
input MAT2A
IP MAT2B
IP FLAG
IP P300
input FLAG
input MAT2B
Input P300

Figure S1

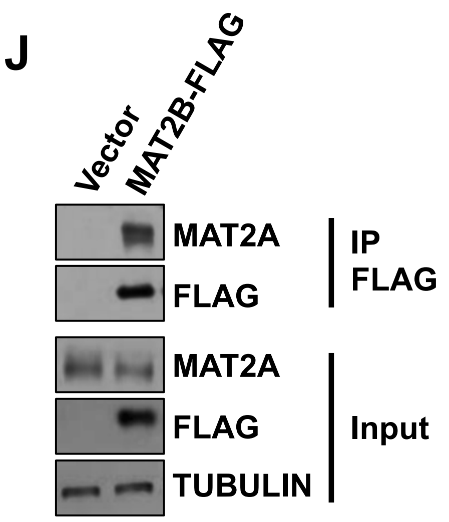
J
Vector
MAT2B-FLAG
MAT2A
FLAG
IP FLAG
MAT2A
FLAG
TUBULIN
Input

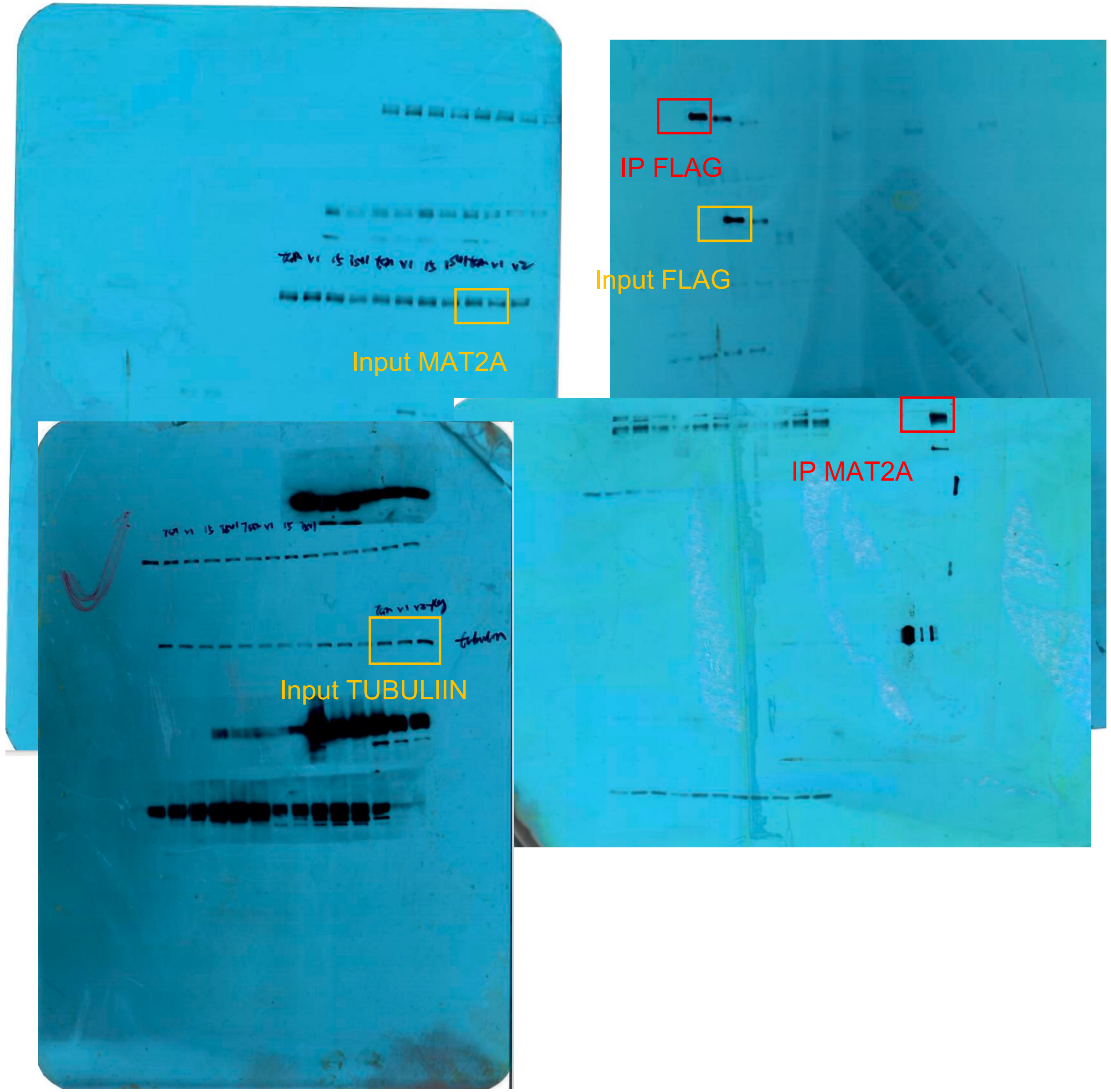
IP FLAG
Input FLAG
Input MAT2A
IP MAT2A
Input TUBULIIN

Figure 2

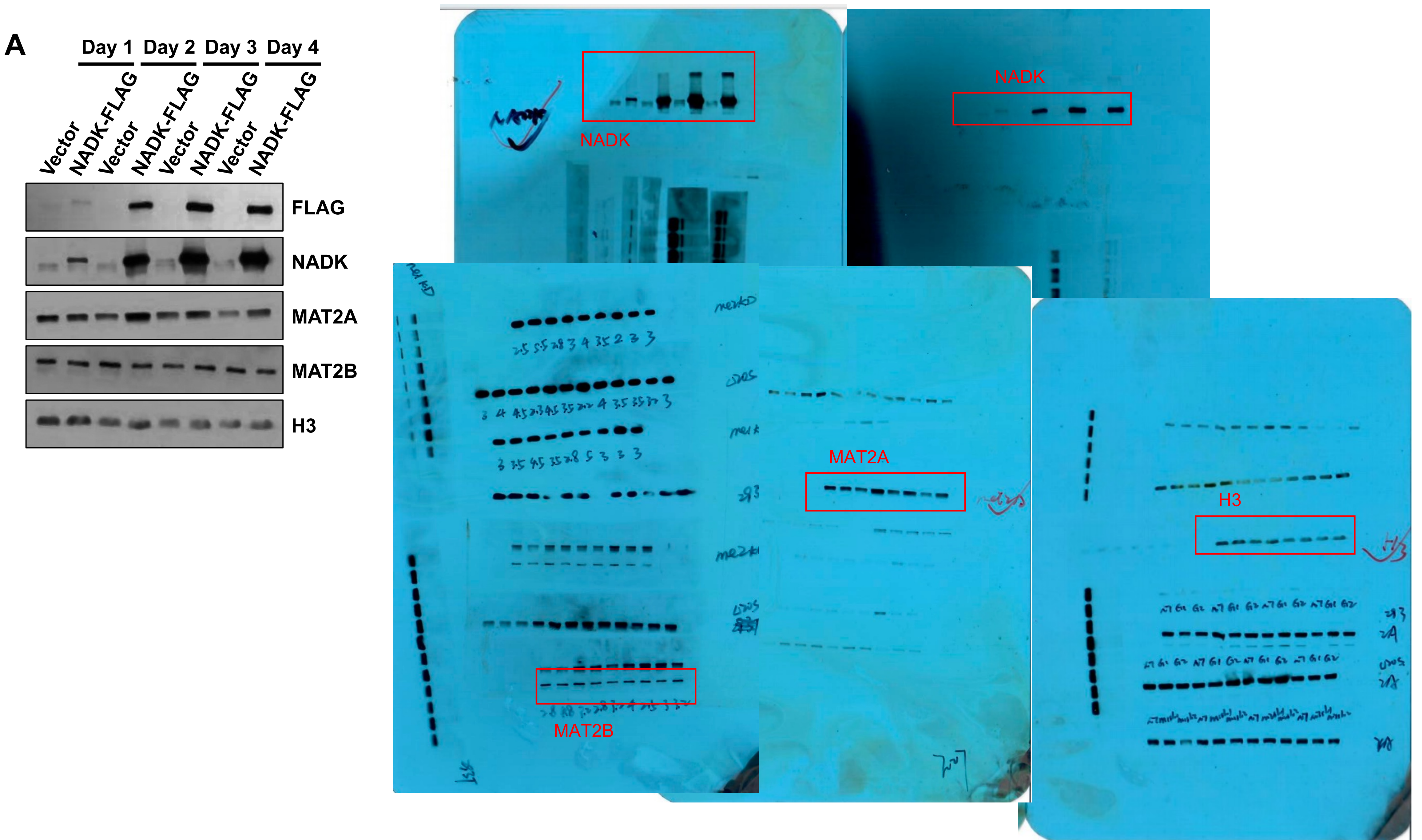
A
Day 1
Day 2
Day 3
Day 4
Vector
NADK-FLAG
Vector
NADK-FLAG
Vector
NADK-FLAG
Vector
NADK-FLAG
FLAG
NADK
MAT2A
MAT2B
H3
NADK
NADK
MAT2A
H3
MAT2B

Figure 2

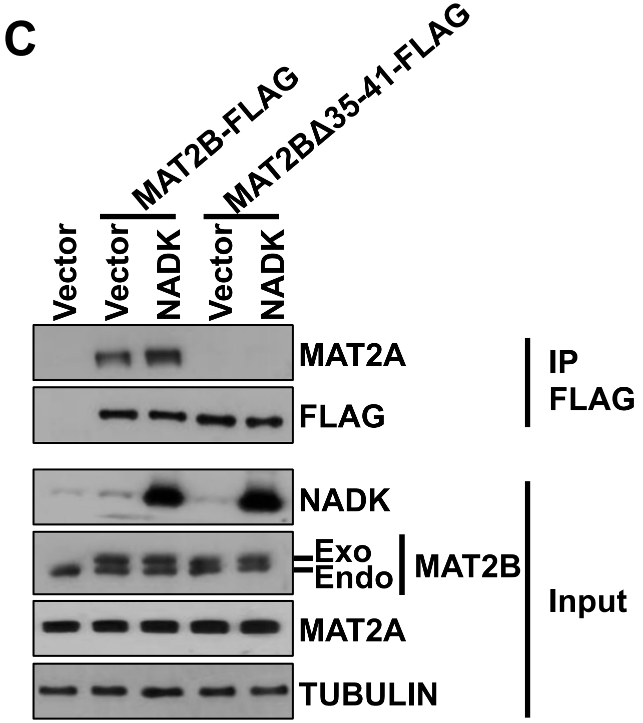
C
MAT2B-FLAG
MAT2BΔ35-41-FLAG
Vector
Vector
NADK
Vector
NADK
MAT2A
FLAG
IP
FLAG
NADK
Exo
Endo
MAT2B
MAT2A
TUBULIN
Input

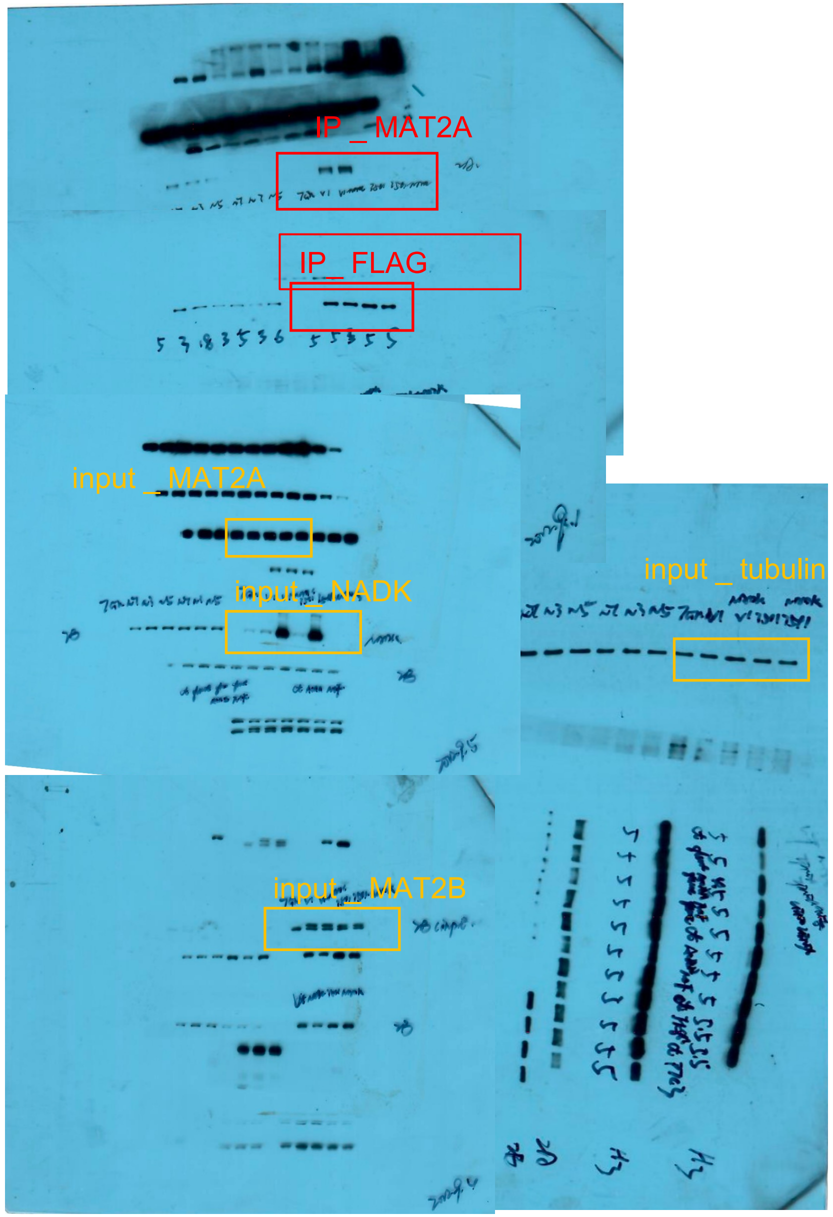
IP _ MAT2A
IP_ FLAG
input _ MAT2A
input _ NADK
input _ tubulin
input _ MAT2B

Figure 2

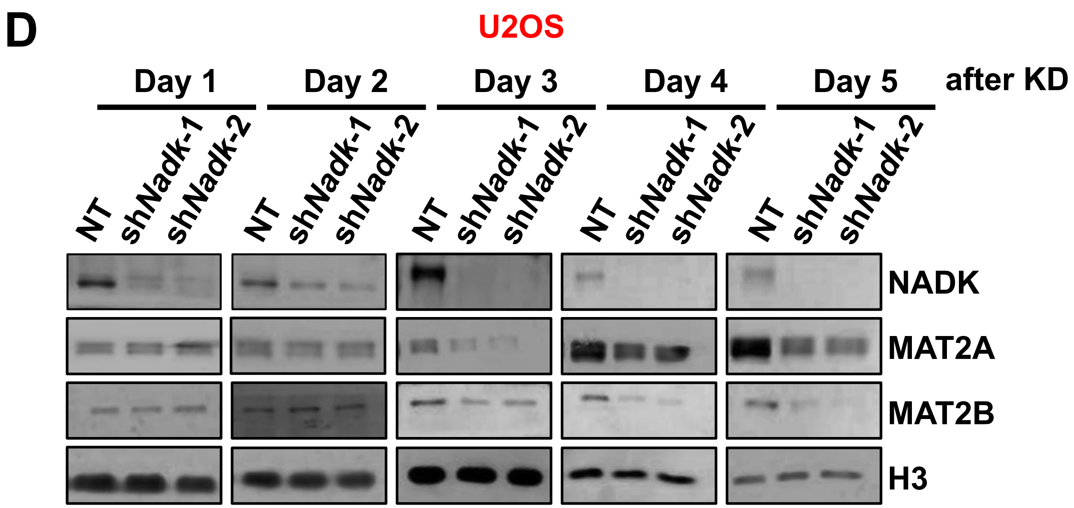
D
U2OS
Day 1
Day 2
Day 3
Day 4
Day 5
after KD
NT
shNadk-1
shNadk-2
NADK
MAT2A
MAT2B
H3

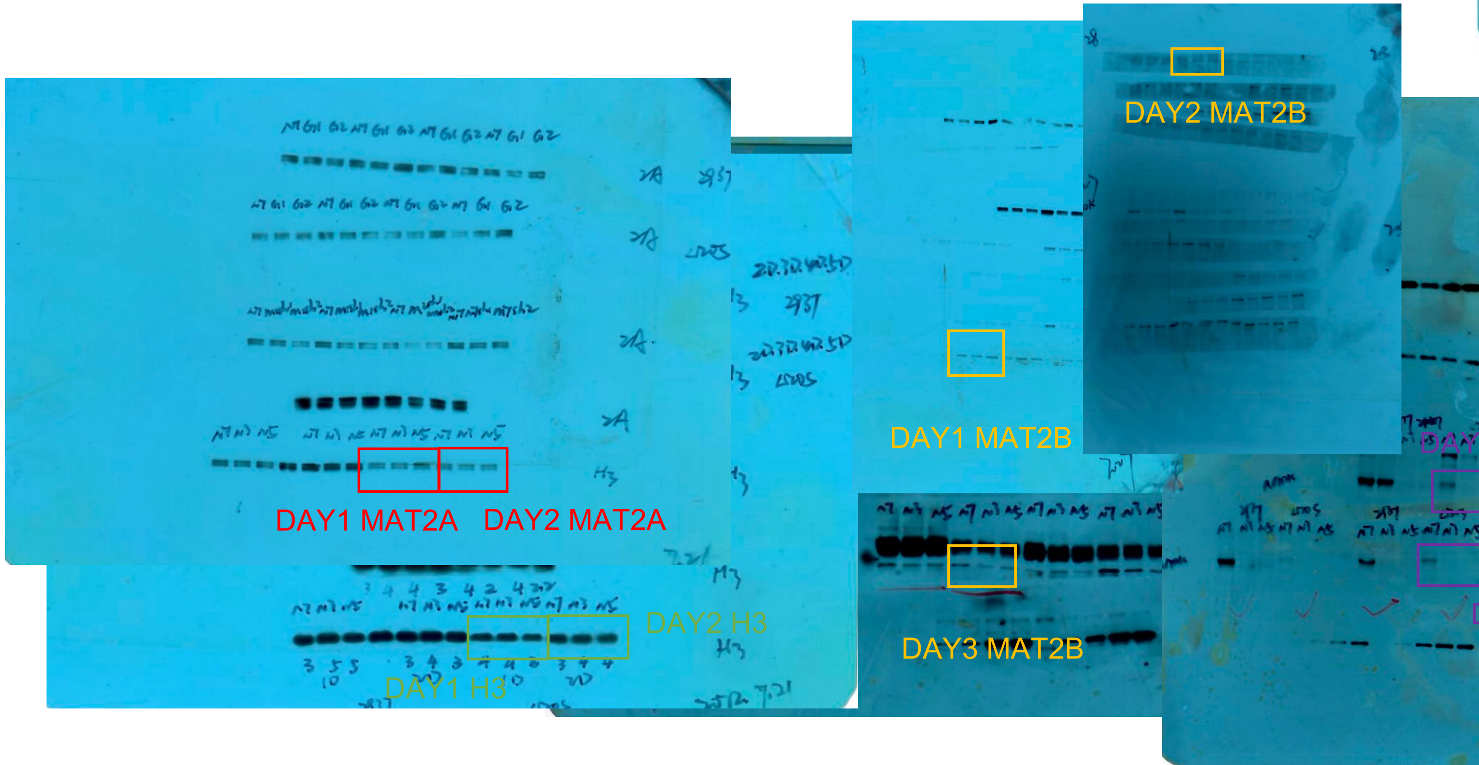

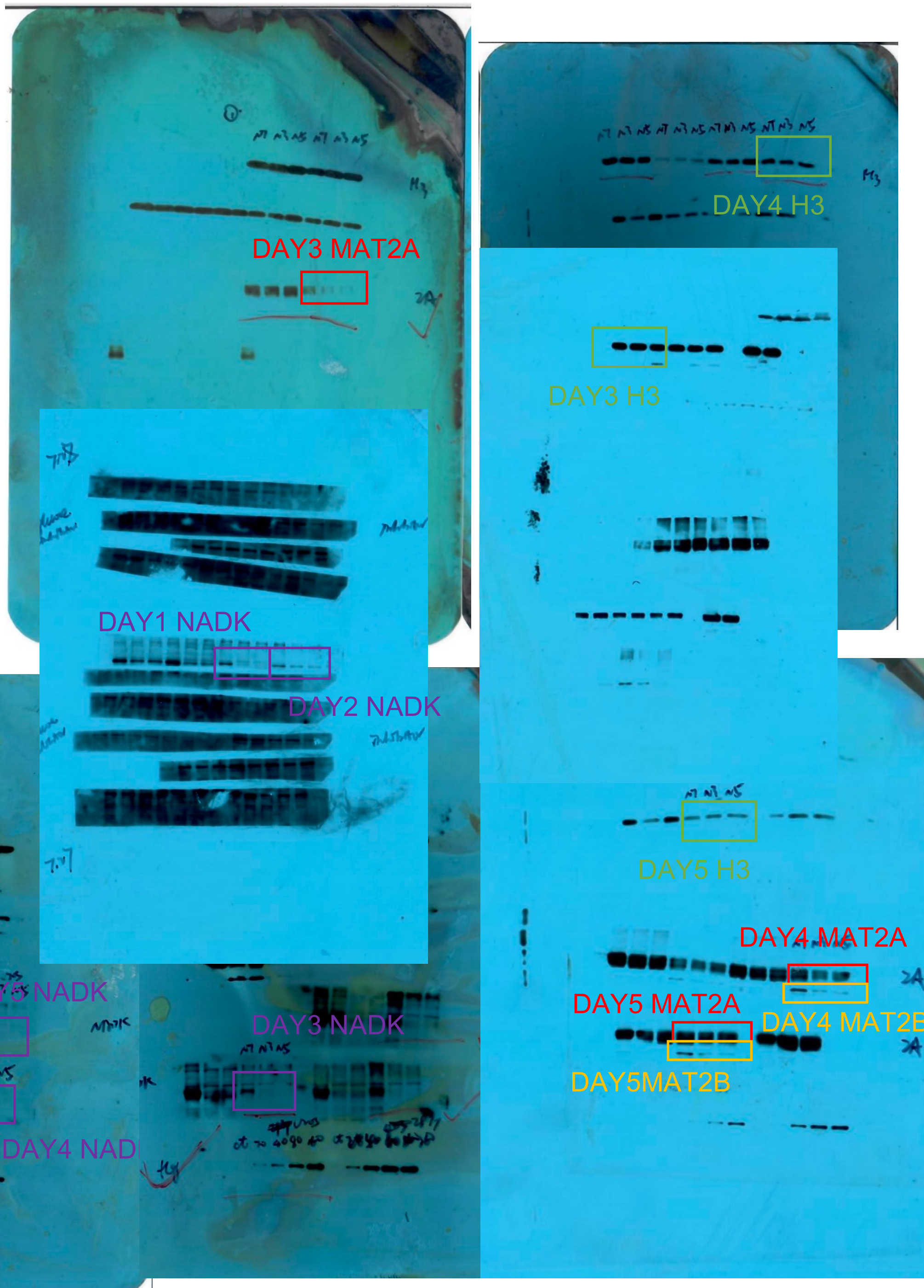
DAY3 MAT2A
DAY4 H3
DAY3 H3
DAY1 NADK
DAY2 NADK
DAY2 MAT2B
DAY1 MAT2B
DAY1 MAT2A
DAY2 MAT2A
DAY2 H3
DAY1 H3
DAY3 MAT2B
DAY5 NADK
DAY4 NAD
DAY3 NADK
DAY5 H3
DAY4 MAT2A
DAY5 MAT2A
DAY4 MAT2B
DAY5MAT2B

Figure 2

F

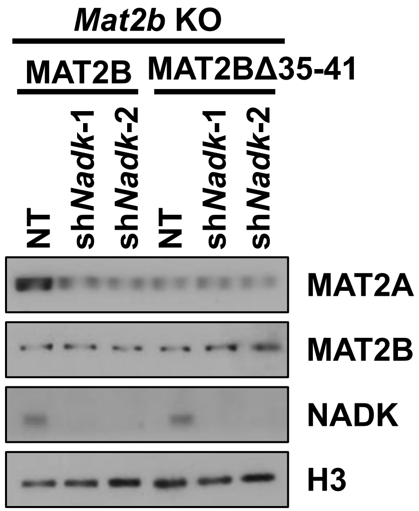

Mat2b KO
MAT2B
MAT2BΔ35-41
NT
sh*Nadk*-1
sh*Nadk*-2
NT
sh*Nadk*-1
sh*Nadk*-2
MAT2A
MAT2B
NADK
H3

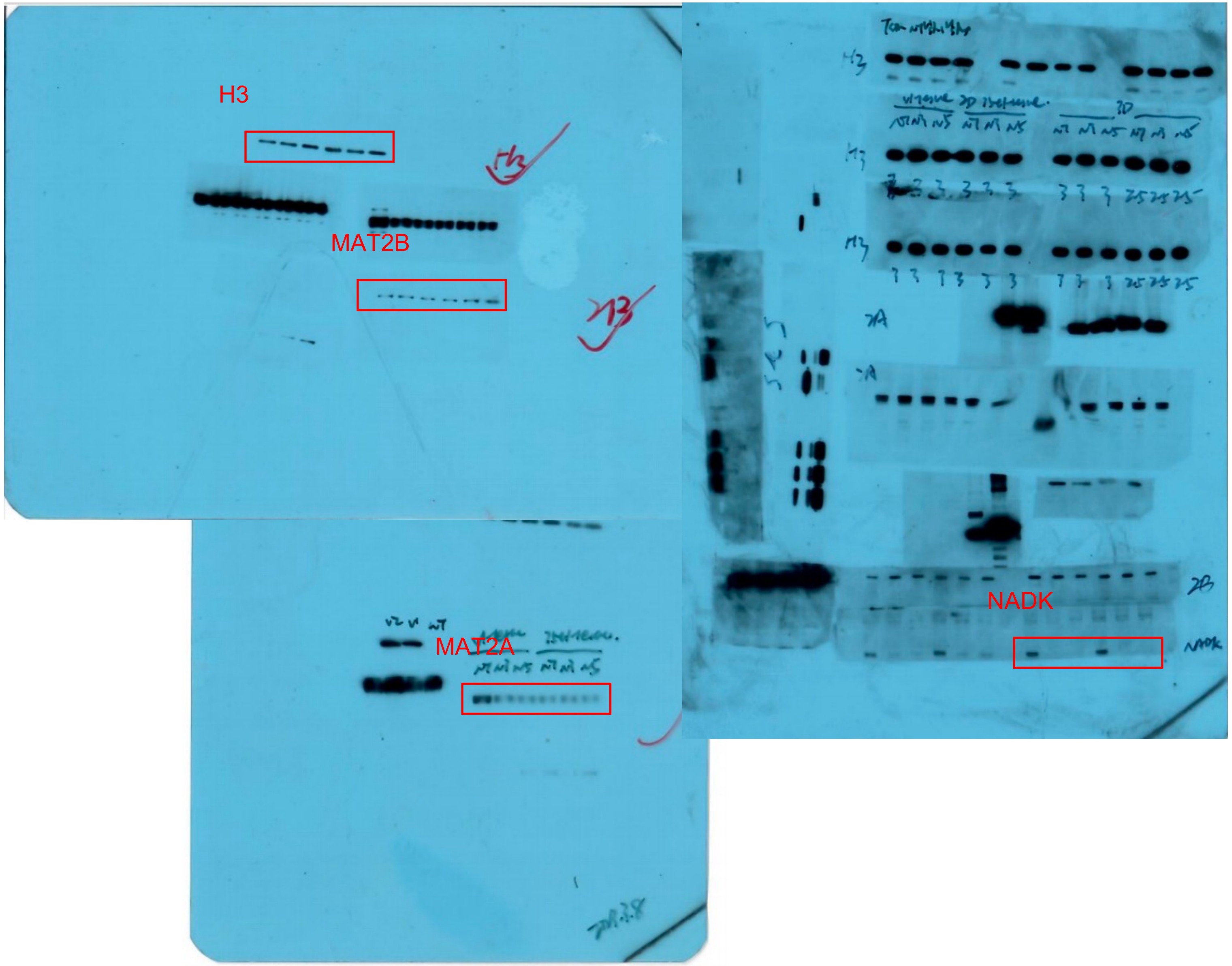

H3
MAT2B
MAT2A
NADK

Figure 2

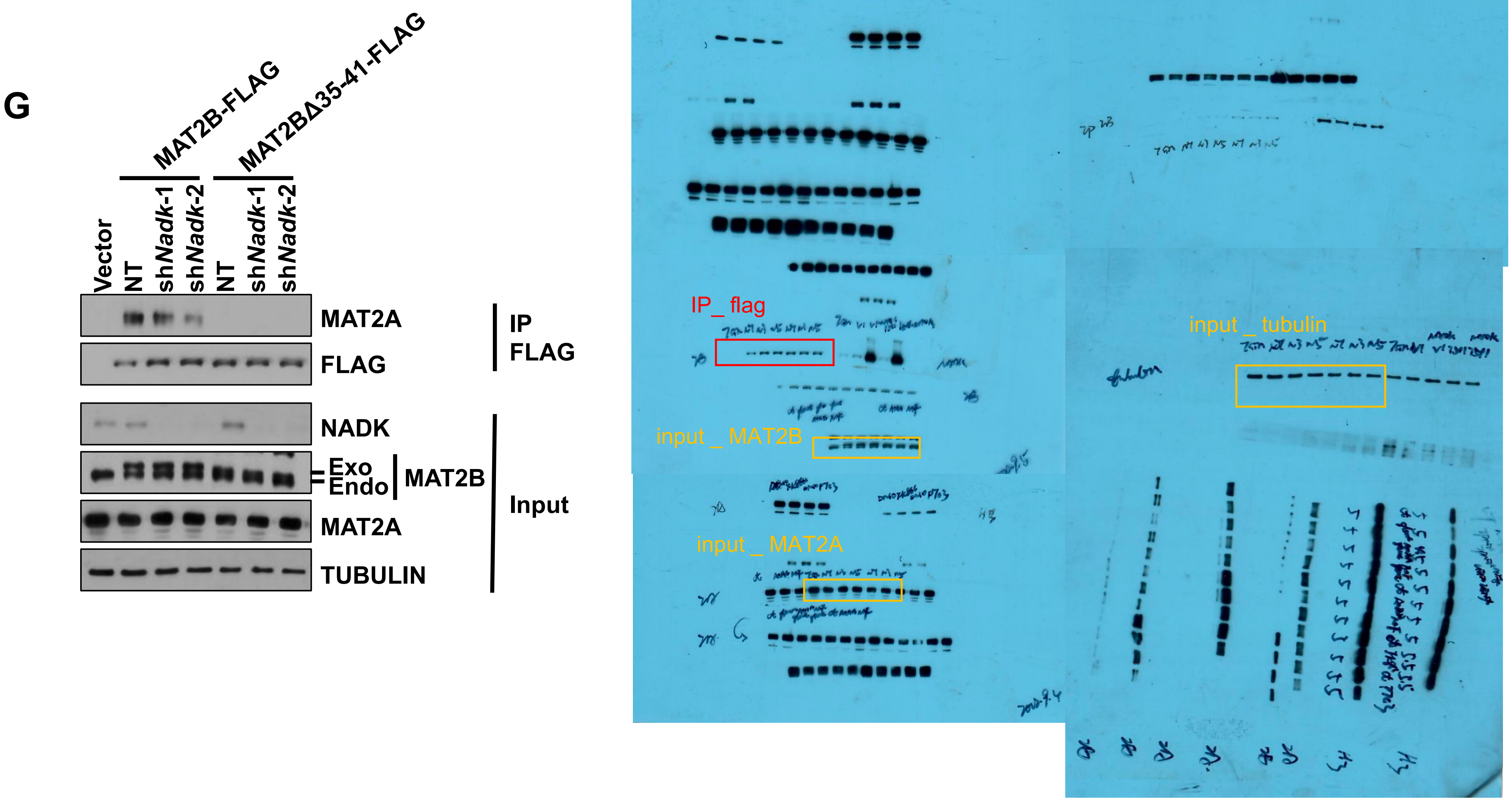
G
MAT2B-FLAG
MAT2BΔ35-41-FLAG
Vector
NT
shNadk-1
shNadk-2
NT
shNadk-1
shNadk-2
MAT2A
FLAG
IP
FLAG
NADK
Exo
Endo
MAT2B
MAT2A
TUBULIN
Input
IP_ MAT2A
input _ NADK
IP_ flag
input _ MAT2B
input _ tubulin
input _ MAT2A

Figure 2

H

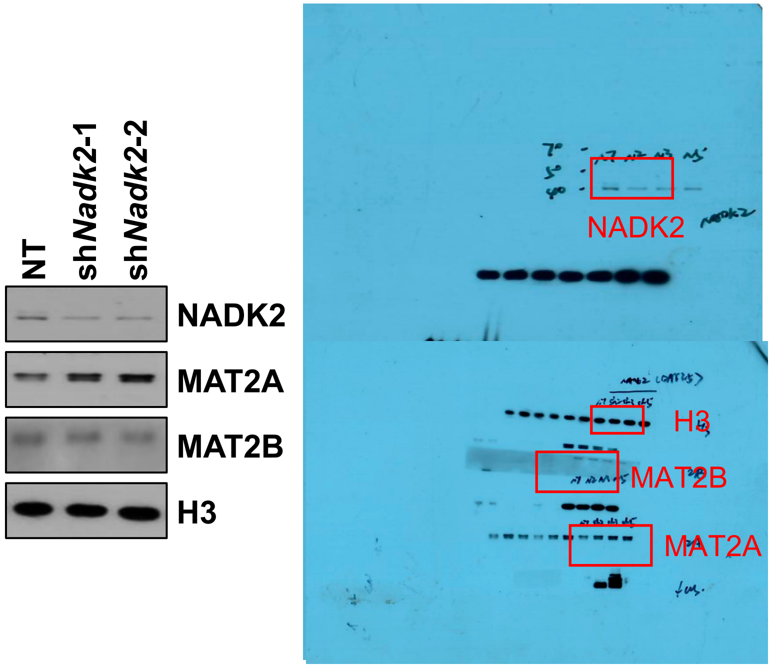
NT
shNadk2-1
shNadk2-2
NADK2
MAT2A
MAT2B
H3
NADK2
H3
MAT2B
MAT2A

J

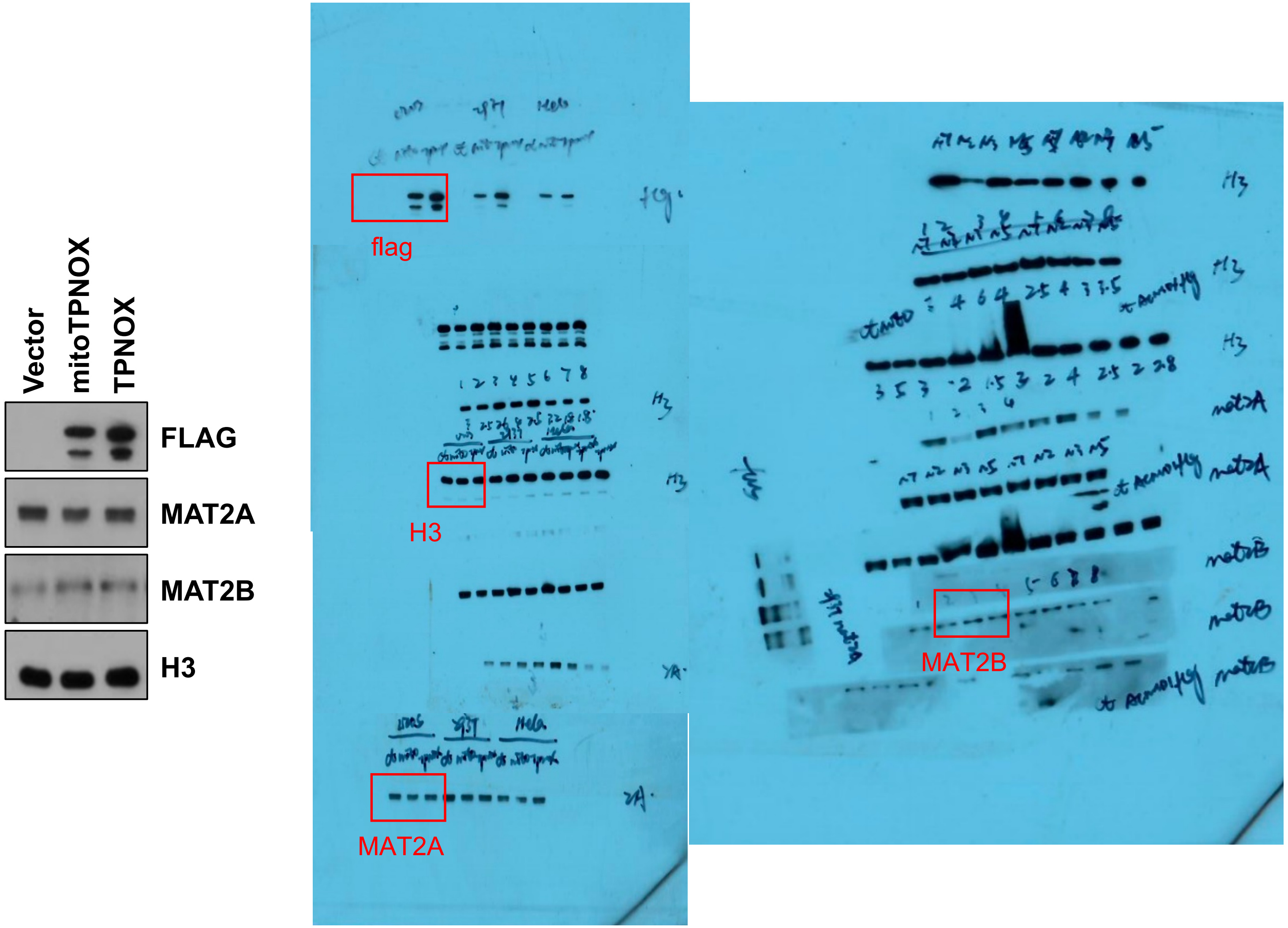
Vector
mitoTPNOX
TPNOX
FLAG
MAT2A
MAT2B
H3
flag
H3
MAT2A
MAT2B

Figure 2

L

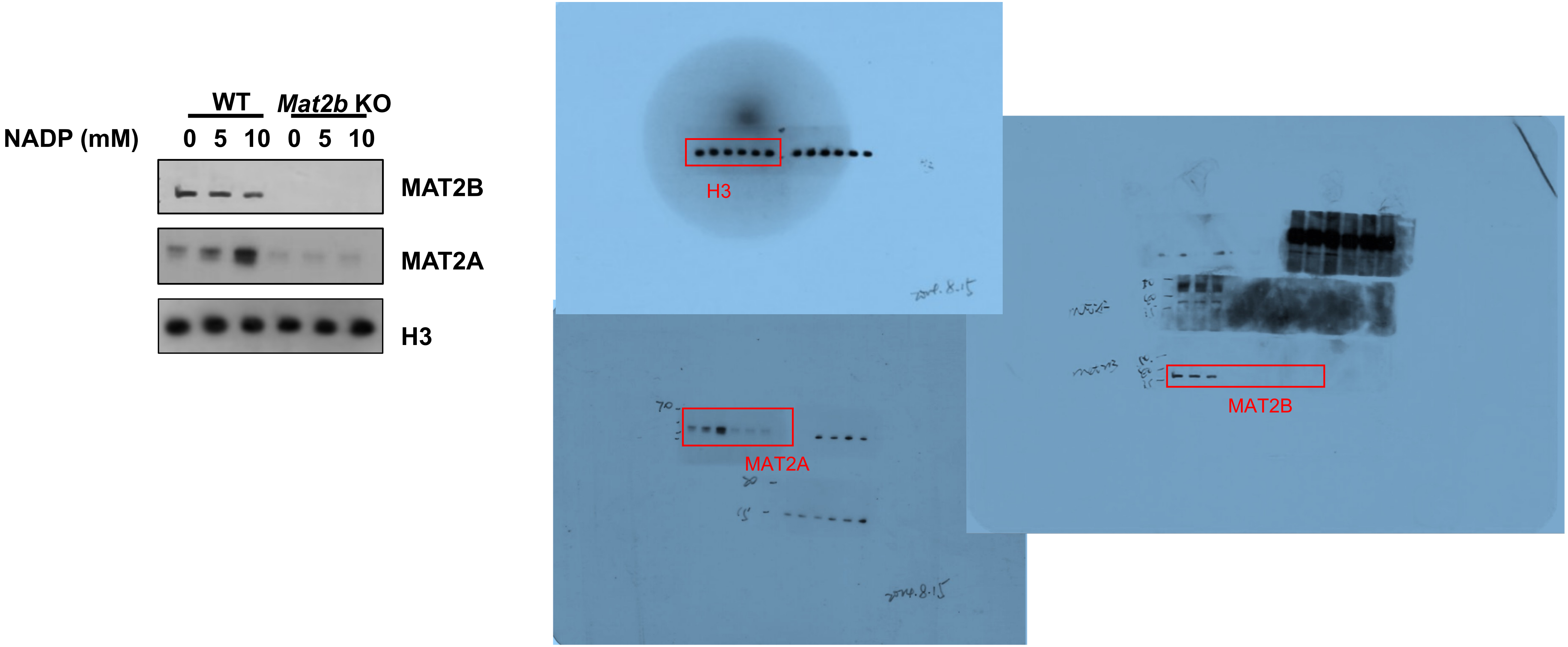

Figure S2

A

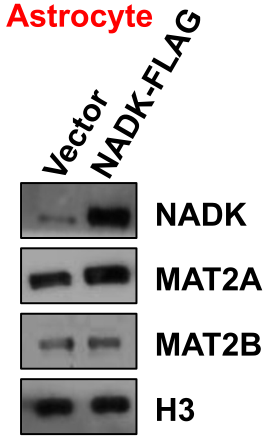
Astrocyte
Vector
NADK-FLAG
NADK
MAT2A
MAT2B
H3

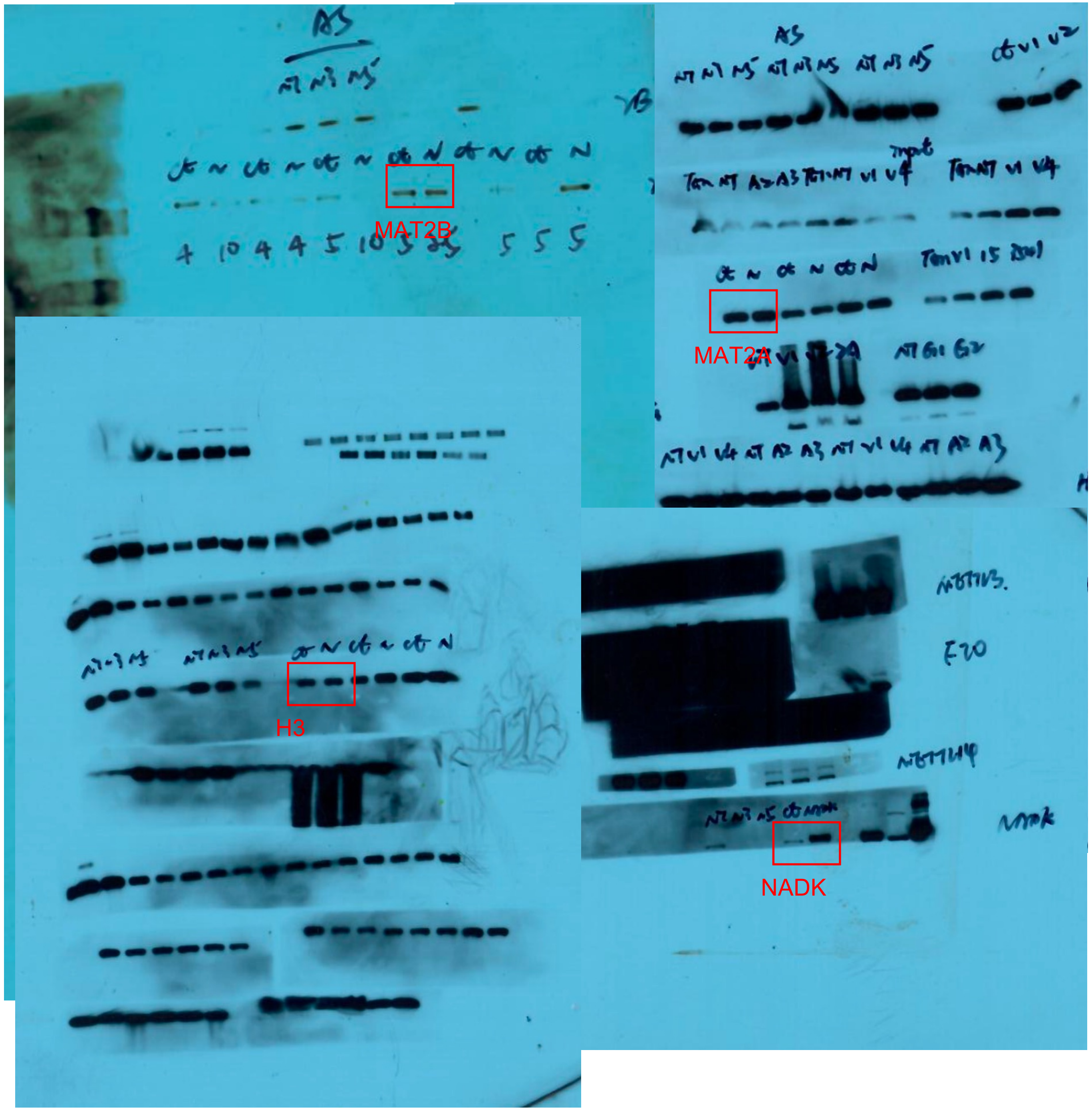
MAT2B
MAT2A
H3
NADK

C

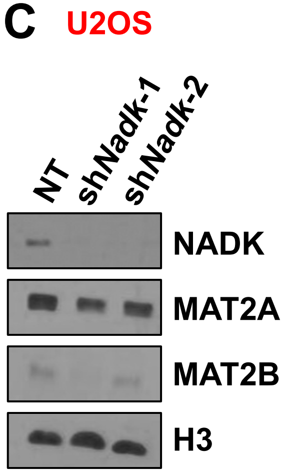
U2OS
NT
shNadk-1
shNadk-2
NADK
MAT2A
MAT2B
H3

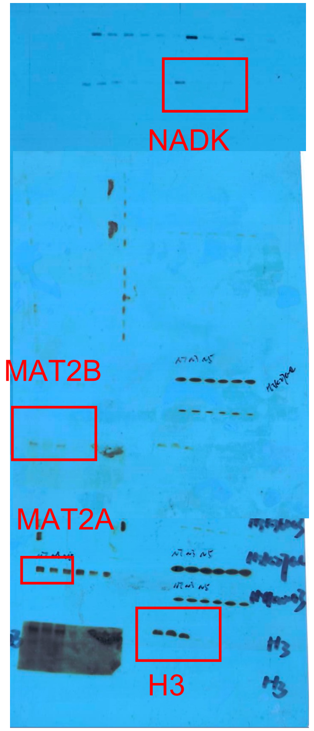
NADK
MAT2B
MAT2A
H3

Figure S2

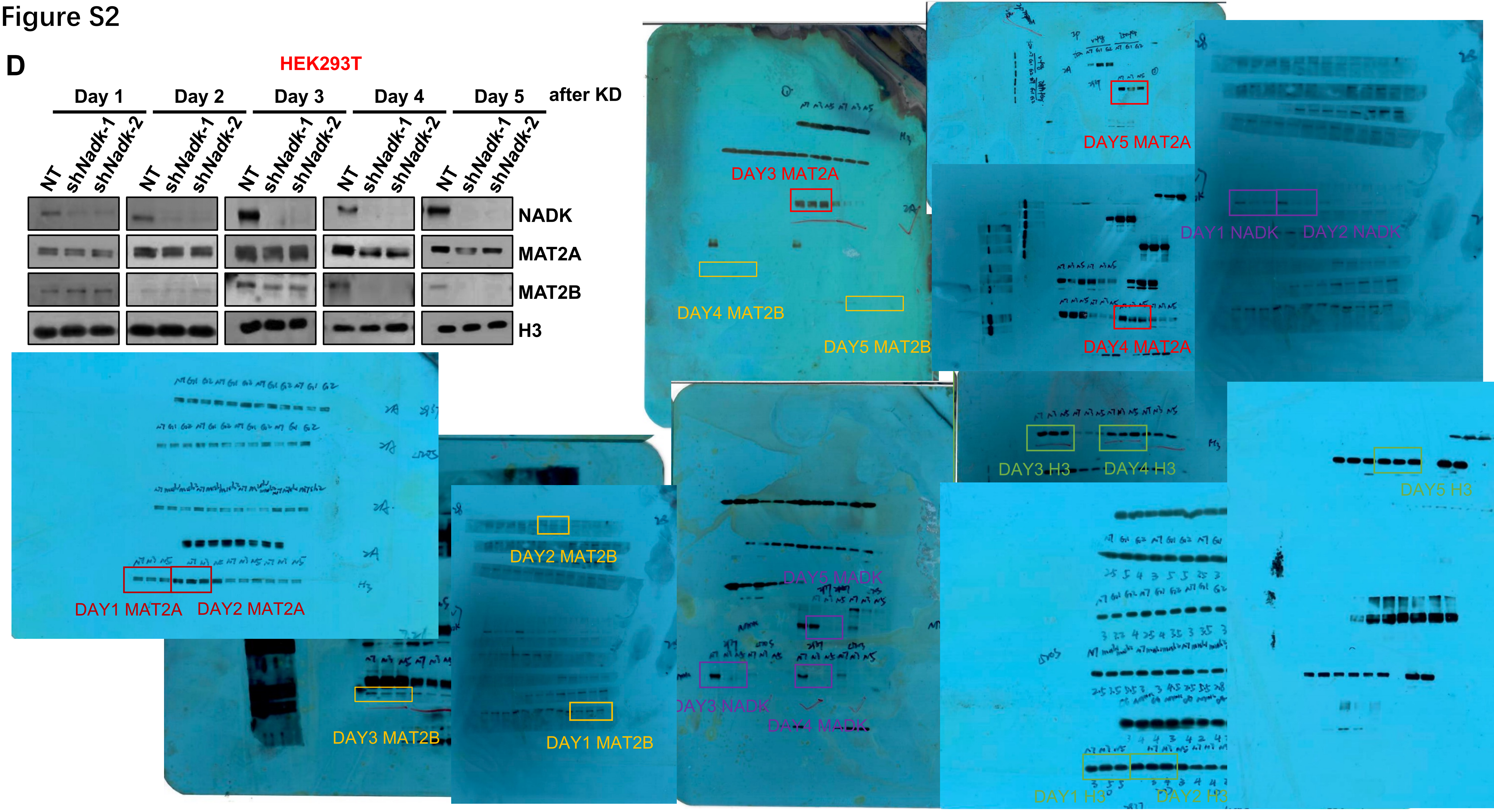
D
HEK293T
Day 1
Day 2
Day 3
Day 4
Day 5
after KD
NT
shNadk-1
shNadk-2
NADK
MAT2A
MAT2B
H3
DAY3 MAT2A
DAY5 MAT2A
DAY4 MAT2B
DAY5 MAT2B
DAY1 NADK
DAY2 NADK
DAY4 MAT2A
DAY1 MAT2A
DAY2 MAT2A
DAY2 MAT2B
DAY3 MAT2B
DAY1 MAT2B
DAY3 NADK
DAY4 NADK
DAY5 NADK
DAY3 H3
DAY4 H3
DAY5 H3
DAY1 H3
DAY2 H3

Figure S2

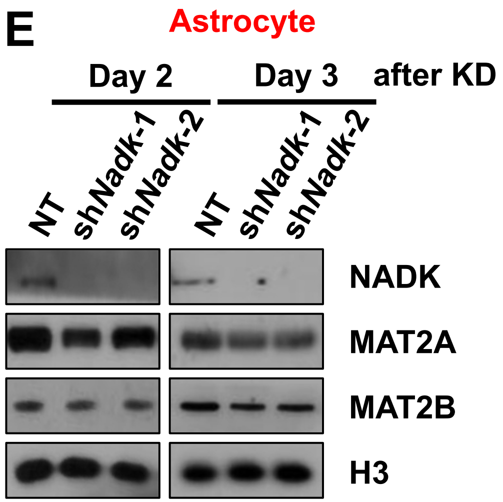
E
Astrocyte
Day 2
Day 3
after KD
NT
shNadk-1
shNadk-2
NT
shNadk-1
shNadk-2
NADK
MAT2A
MAT2B
H3

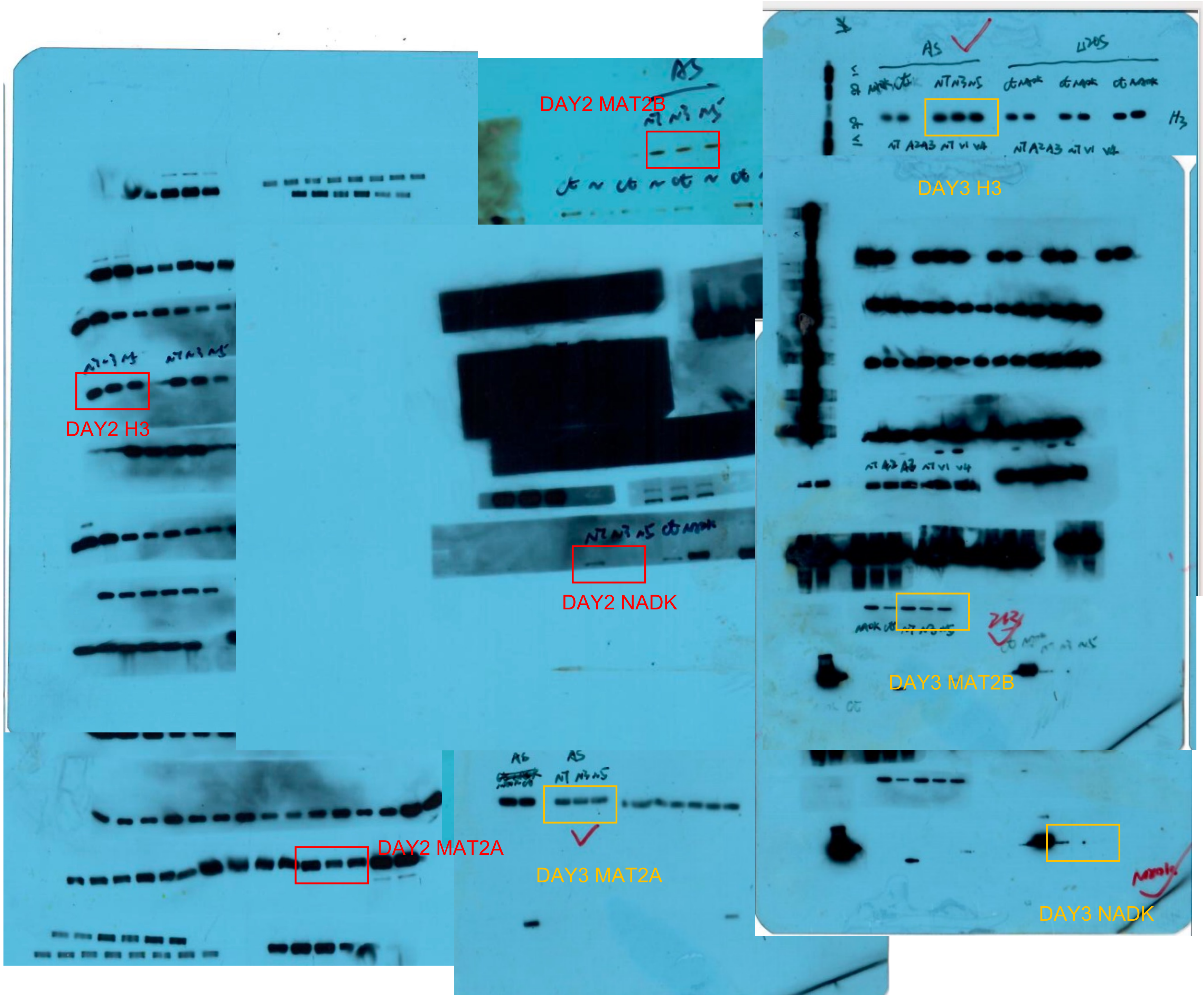
DAY2 MAT2B
DAY3 H3
DAY2 H3
DAY2 NADK
DAY3 MAT2B
DAY2 MAT2A
DAY3 MAT2A
DAY3 NADK

Figure S2

H

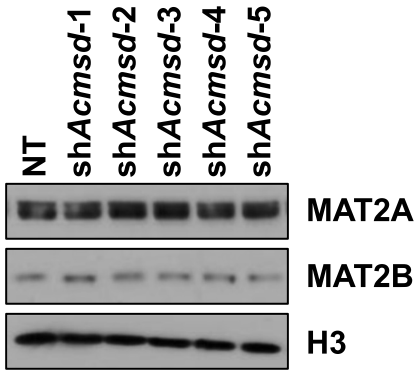
NT
shAcmsd-1
shAcmsd-2
shAcmsd-3
shAcmsd-4
shAcmsd-5
MAT2A
MAT2B
H3

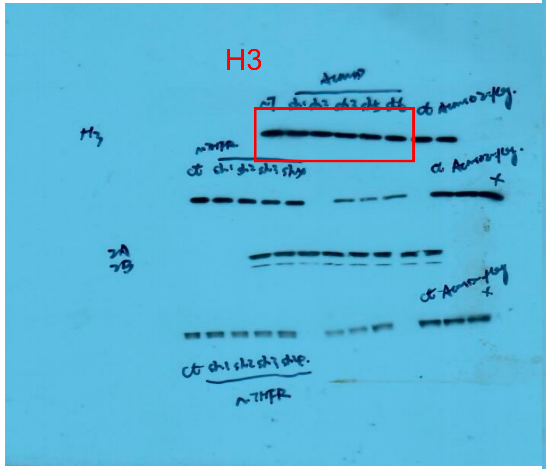
H3

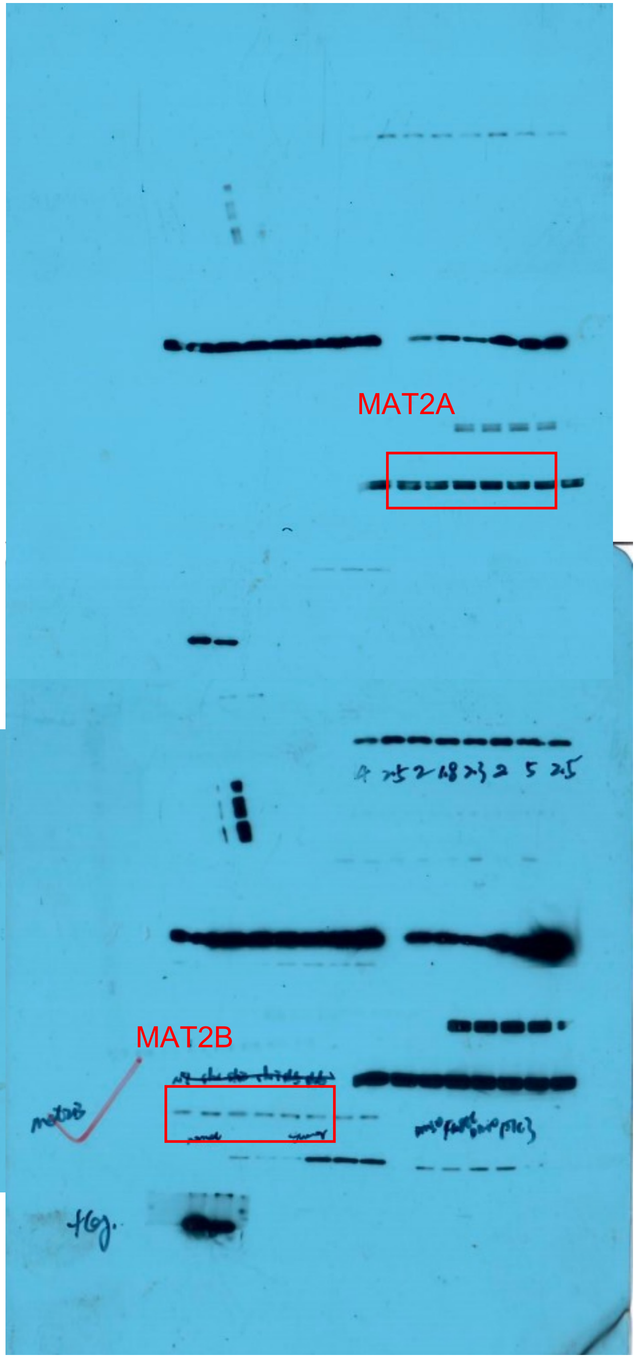
MAT2A
MAT2B

I

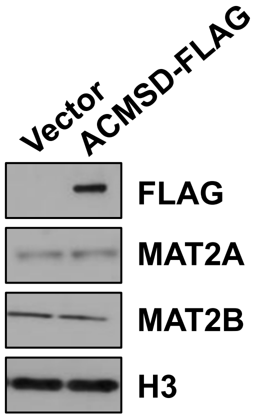
Vector
ACMSD-FLAG
FLAG
MAT2A
MAT2B
H3

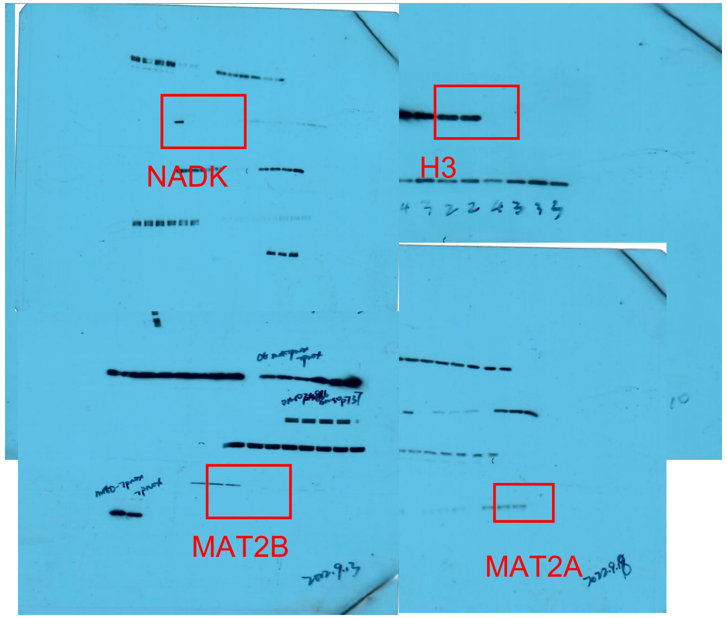
NADK
H3
MAT2B
MAT2A

Figure S2

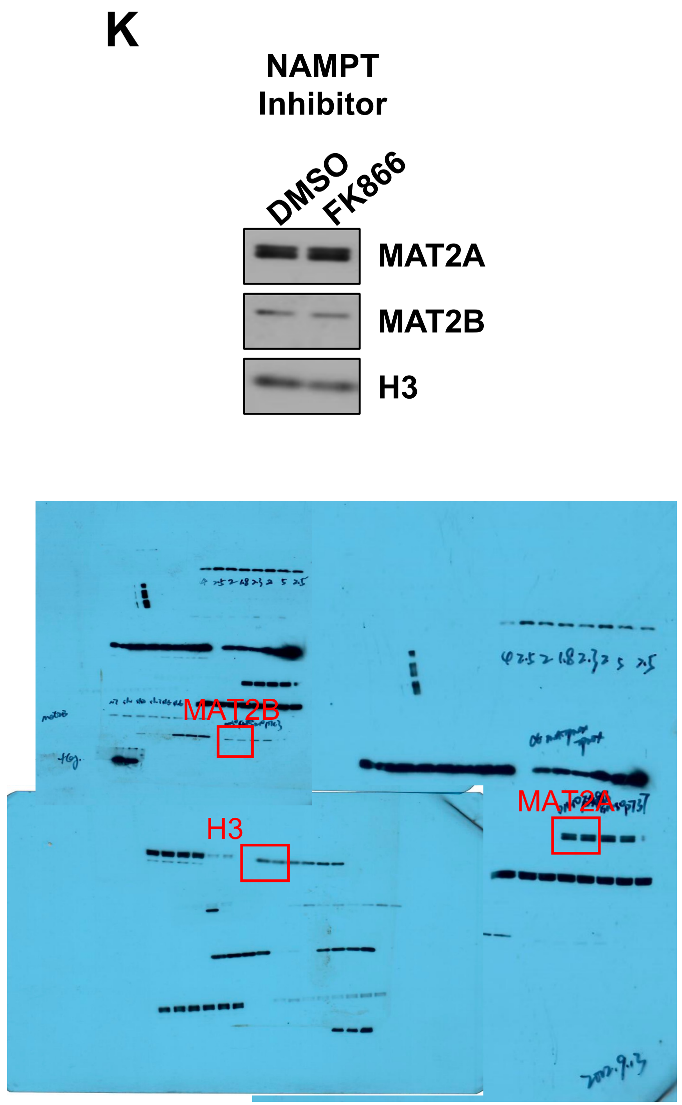
K
NAMPT
Inhibitor
DMSO
FK866
MAT2A
MAT2B
H3
MAT2B
H3
MAT2A

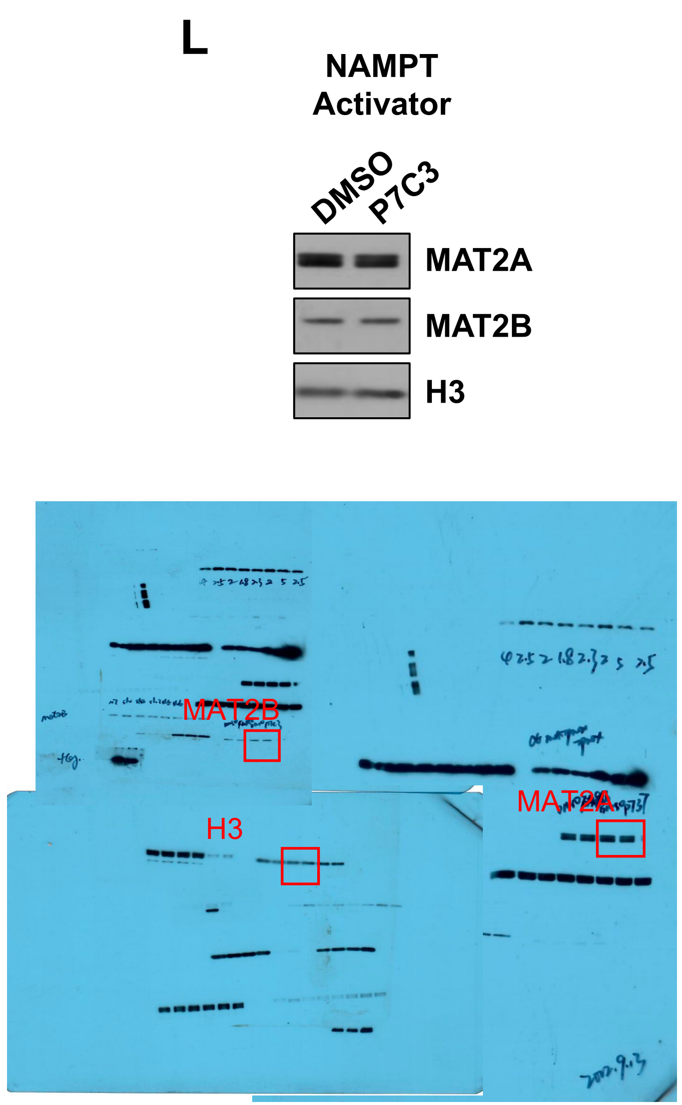
L
NAMPT
Activator
DMSO
P7C3
MAT2A
MAT2B
H3
MAT2B
H3
MAT2A

Figure 3

A

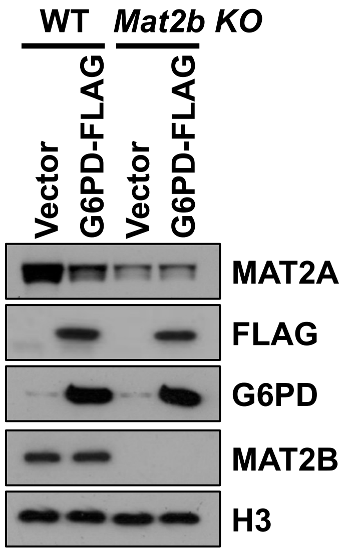
WT
Mat2b KO
Vector
G6PD-FLAG
Vector
G6PD-FLAG
MAT2A
FLAG
G6PD
MAT2B
H3

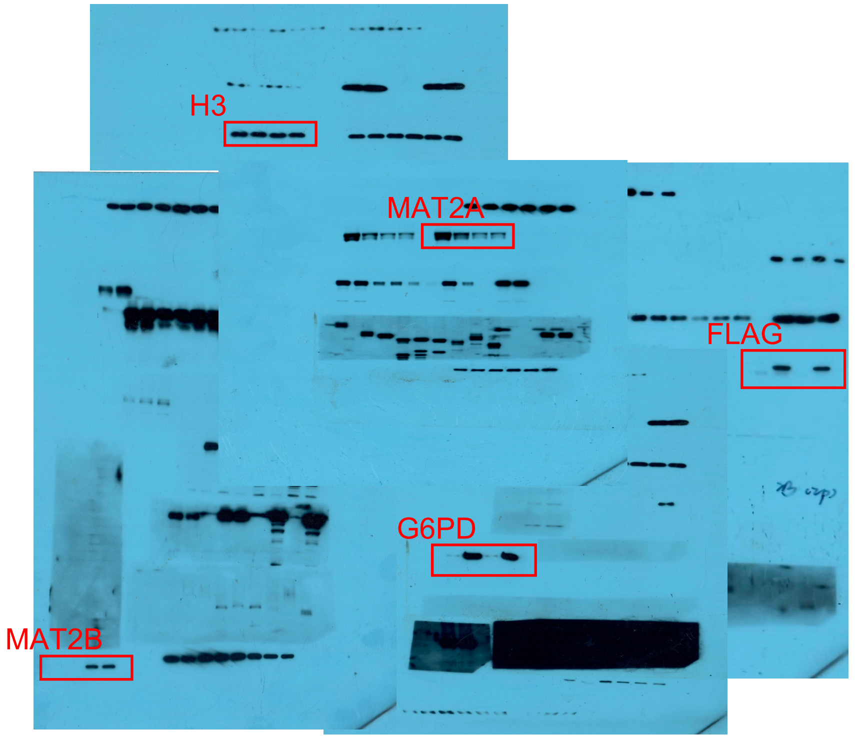
H3
MAT2A
FLAG
G6PD
MAT2B

C

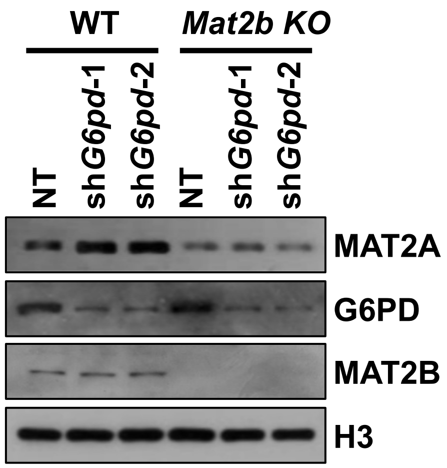
WT
Mat2b KO
NT
sh*G6pd-1*
sh*G6pd-2*
NT
sh*G6pd-1*
sh*G6pd-2*
MAT2A
G6PD
MAT2B
H3

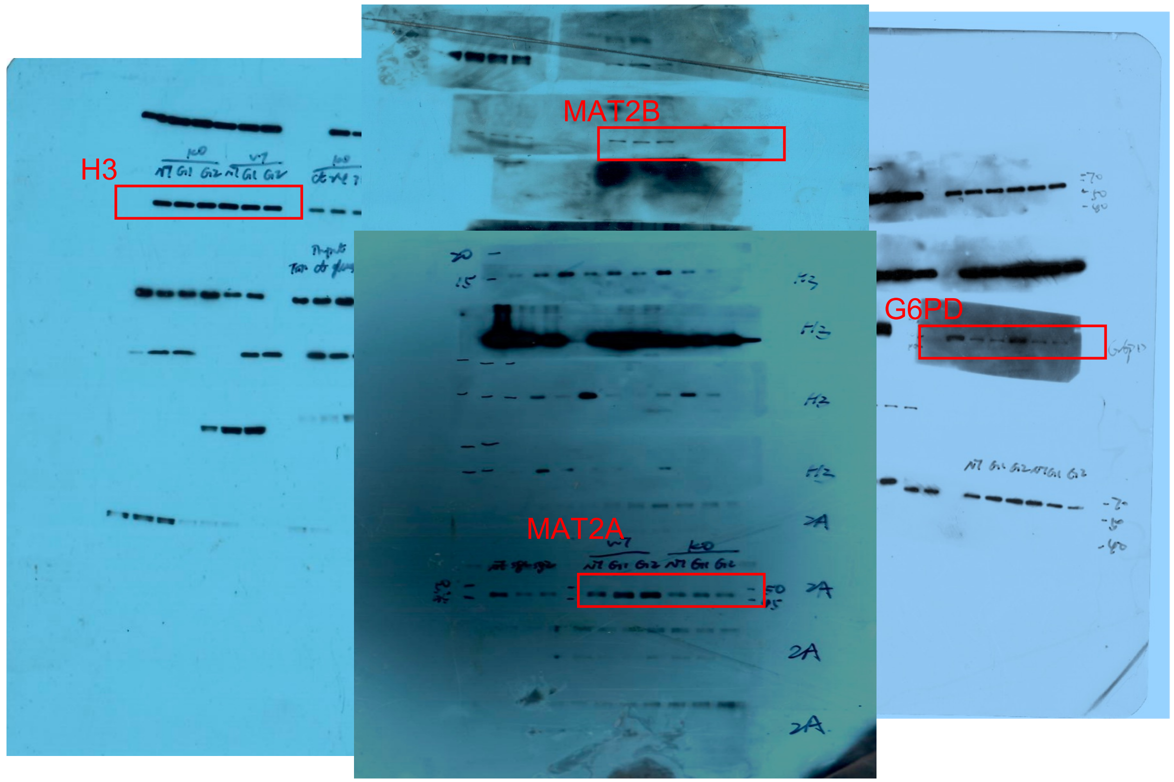
MAT2B
H3
G6PD
MAT2A

Figure 3

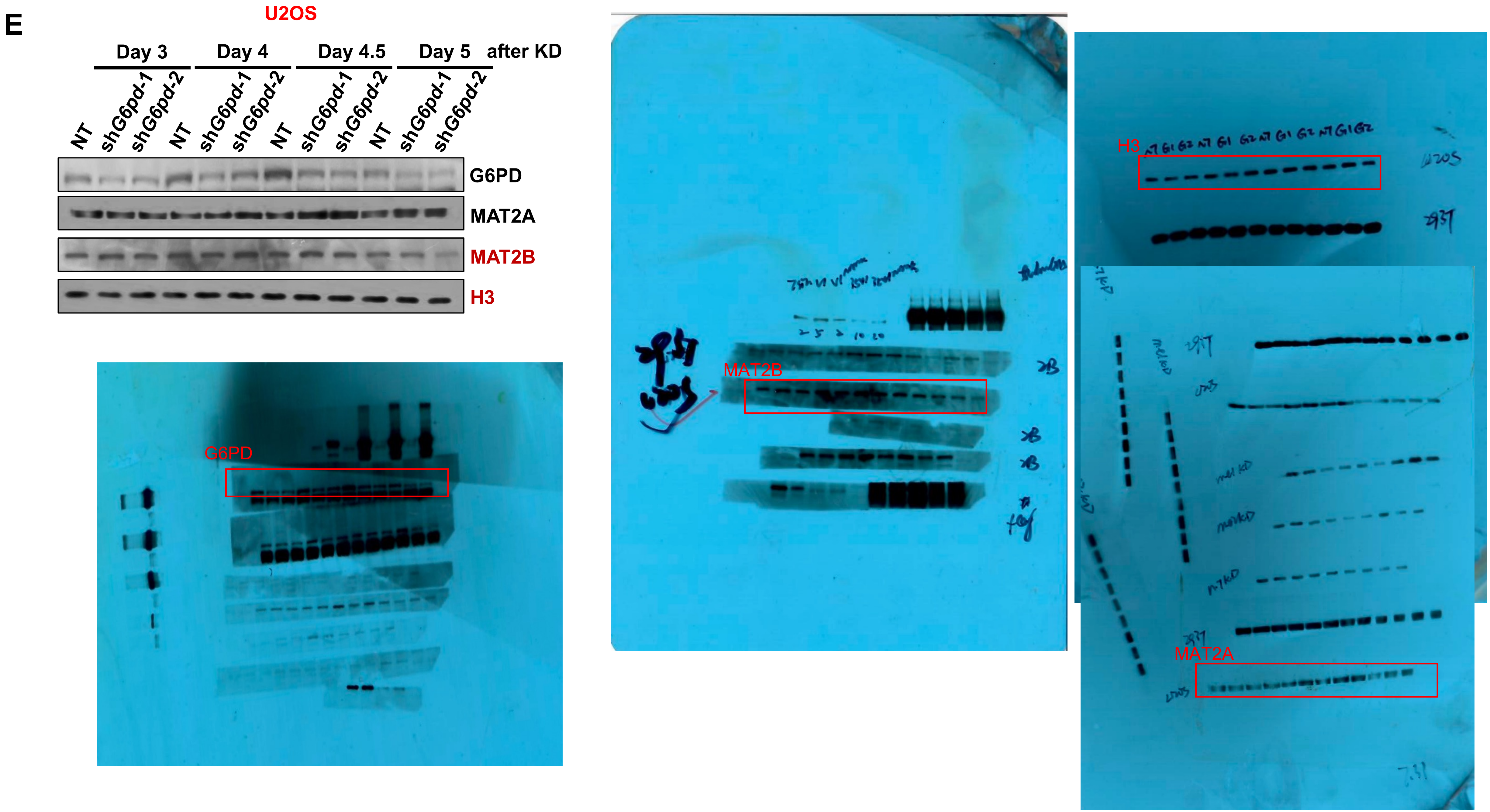
E
U2OS
Day 3
Day 4
Day 4.5
Day 5
after KD
NT
shG6pd-1
shG6pd-2
NT
shG6pd-1
shG6pd-2
NT
shG6pd-1
shG6pd-2
NT
shG6pd-1
shG6pd-2
G6PD
MAT2A
MAT2B
H3
G6PD
MAT2B
H3
MAT2A

Figure 3

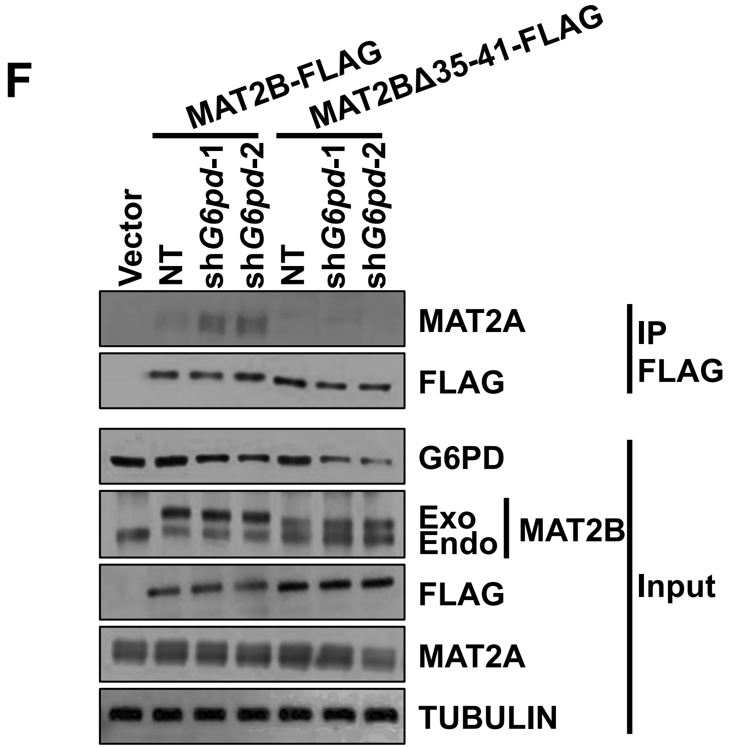
F
MAT2B-FLAG
MAT2BΔ35-41-FLAG
Vector
NT
shG6pd-1
shG6pd-2
NT
shG6pd-1
shG6pd-2
MAT2A
FLAG
IP FLAG
G6PD
Exo
Endo
MAT2B
FLAG
MAT2A
TUBULIN
Input

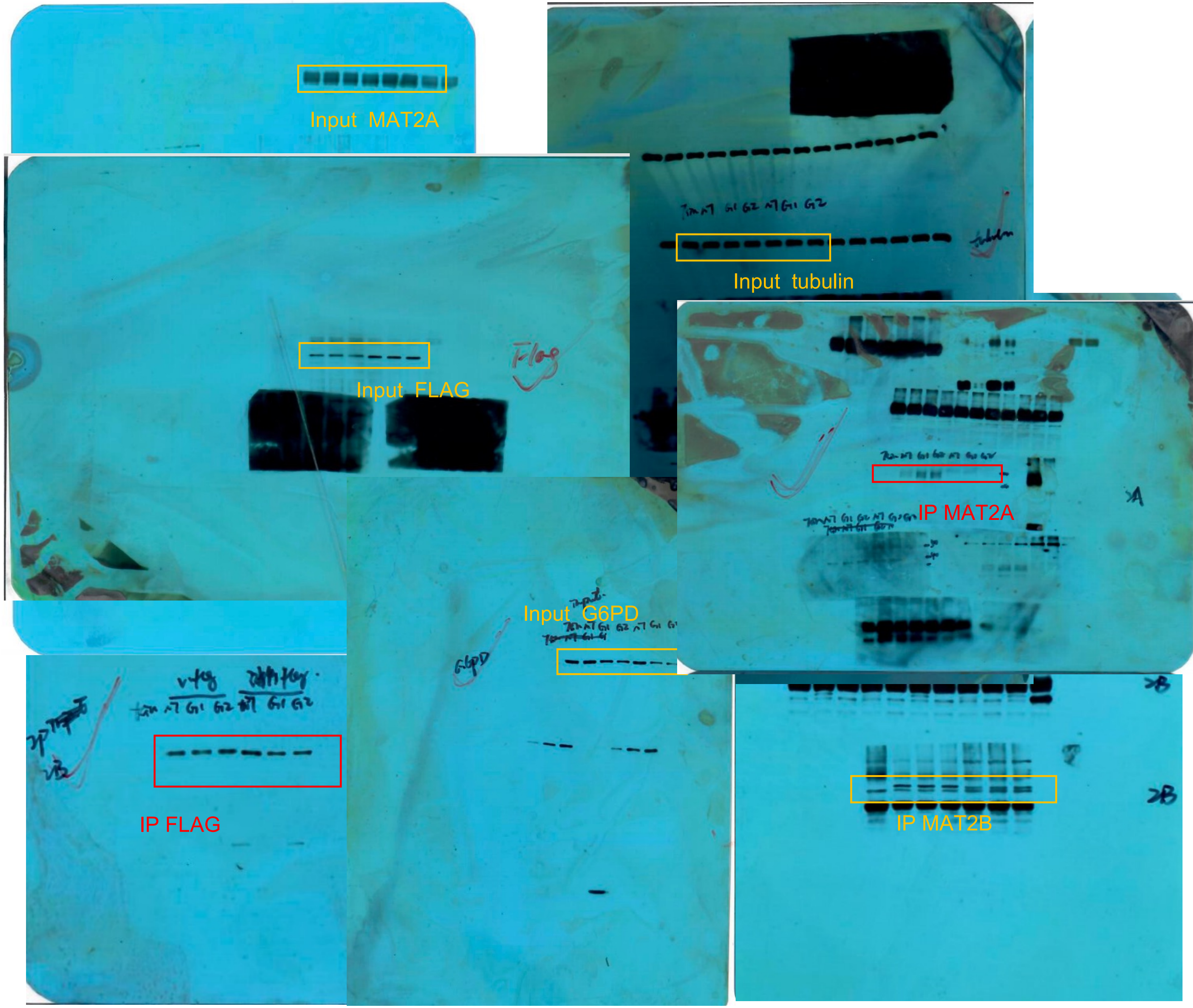
Input MAT2A
Input tubulin
Input FLAG
IP MAT2A
Input G6PD
IP FLAG
IP MAT2B

Figure 3

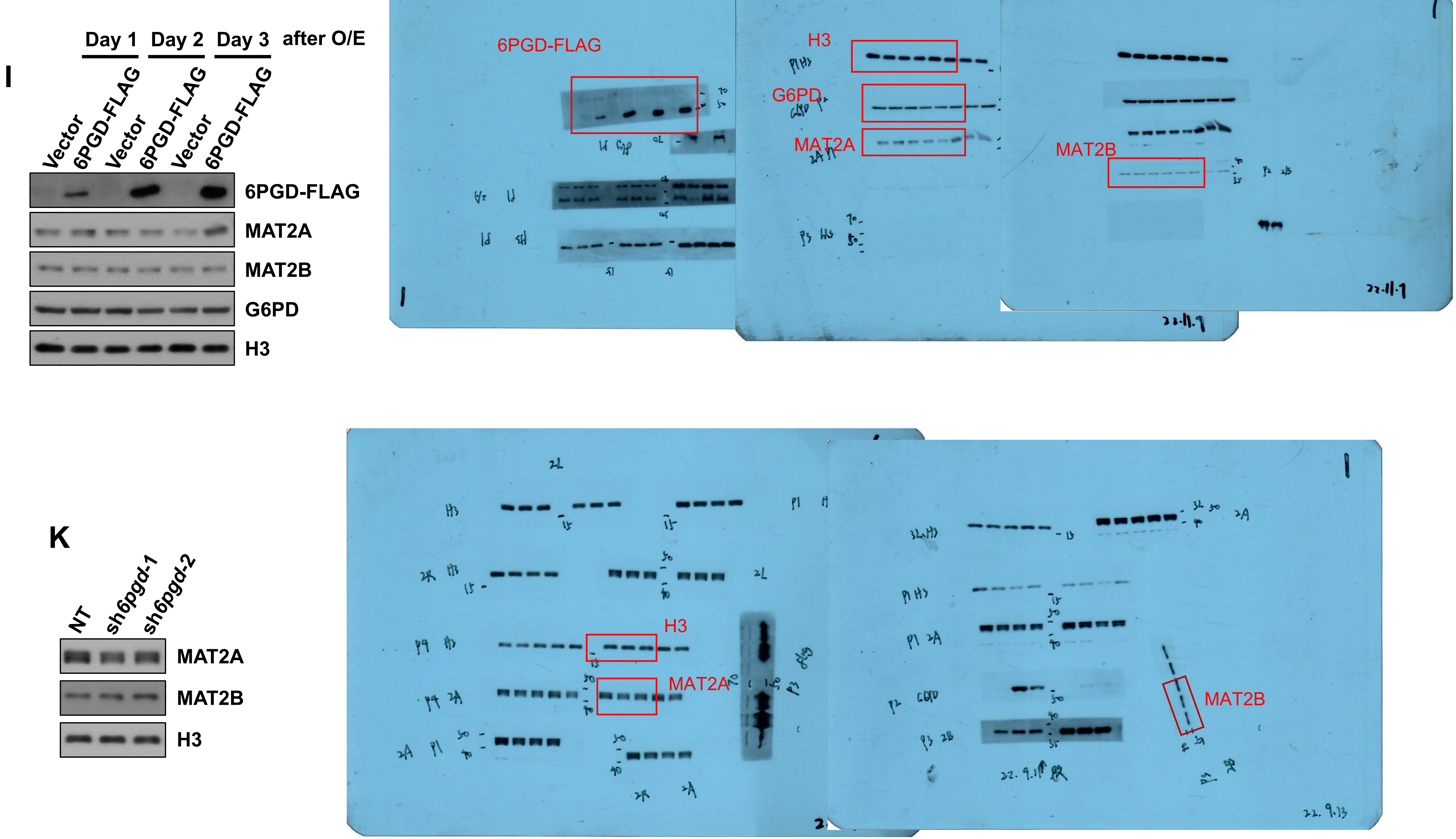
I
Day 1
Day 2
Day 3
after O/E
Vector
6PGD-FLAG
Vector
6PGD-FLAG
Vector
6PGD-FLAG
6PGD-FLAG
MAT2A
MAT2B
G6PD
H3
6PGD-FLAG
H3
G6PD
MAT2A
MAT2B
K
NT
sh6pgd-1
sh6pgd-2
MAT2A
MAT2B
H3
H3
MAT2A
MAT2B

Figure 3

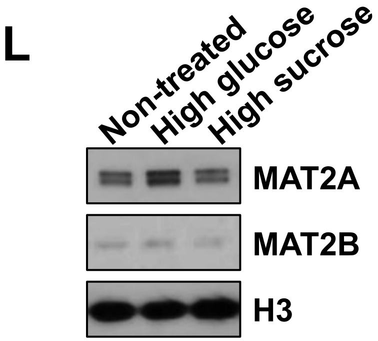
L
Non-treated
High glucose
High sucrose
MAT2A
MAT2B
H3

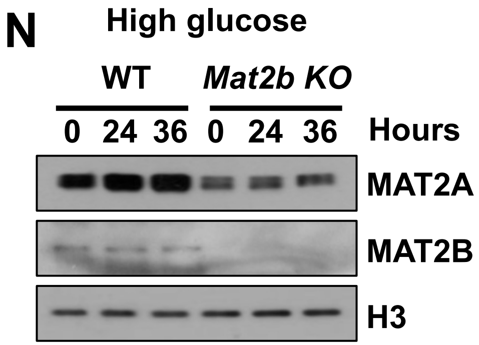
N
High glucose
WT
Mat2b KO
0
24
36
0
24
36
Hours
MAT2A
MAT2B
H3

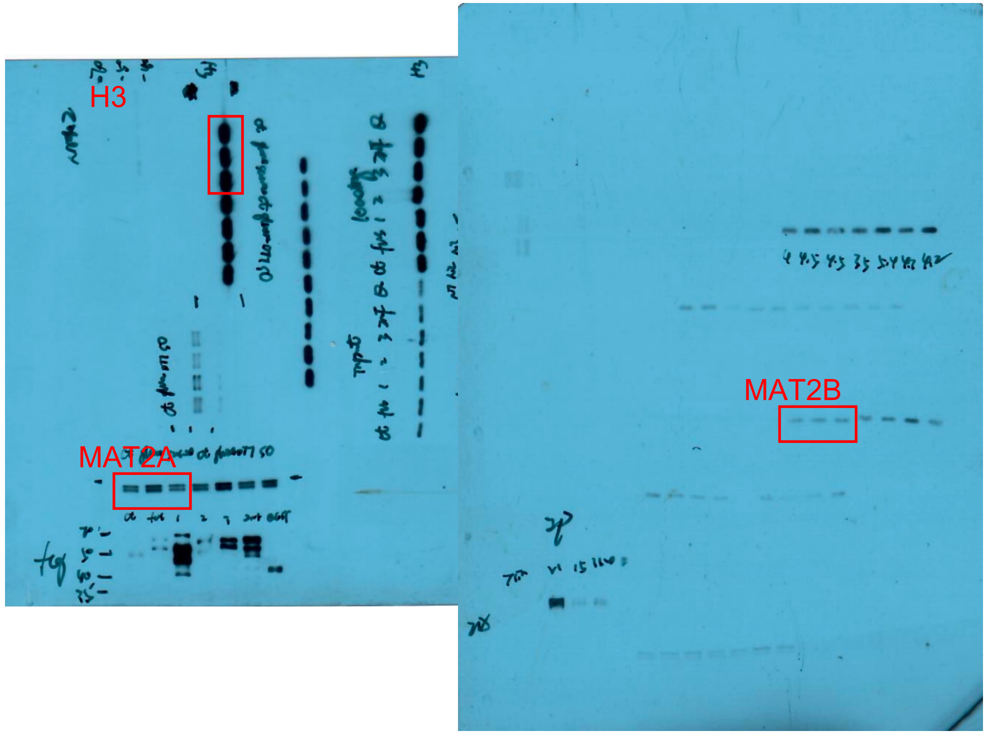
H3
MAT2A
MAT2B

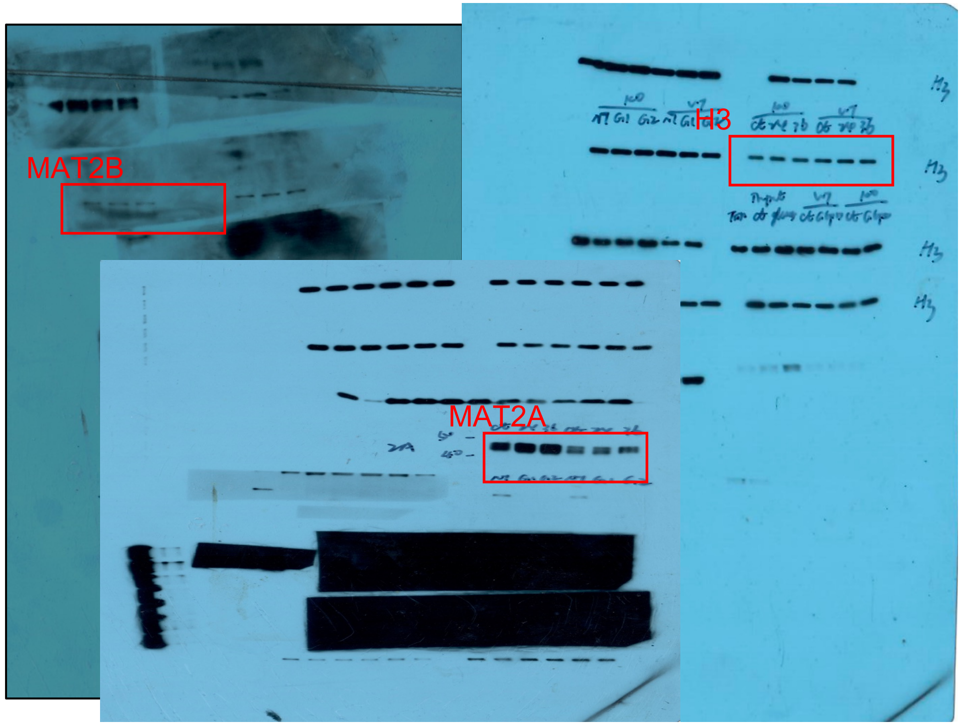
MAT2B
H3
MAT2A

Figure 3

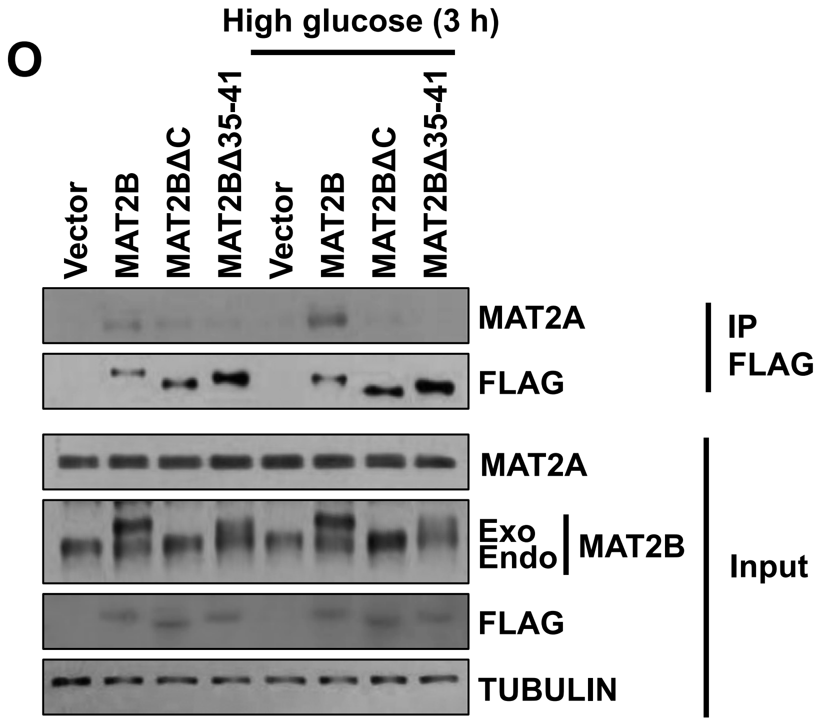
O
High glucose (3 h)
Vector
MAT2B
MAT2BΔC
MAT2BΔ35-41
Vector
MAT2B
MAT2BΔC
MAT2BΔ35-41
MAT2A
FLAG
IP FLAG
MAT2A
Exo
Endo
MAT2B
FLAG
TUBULIN
Input

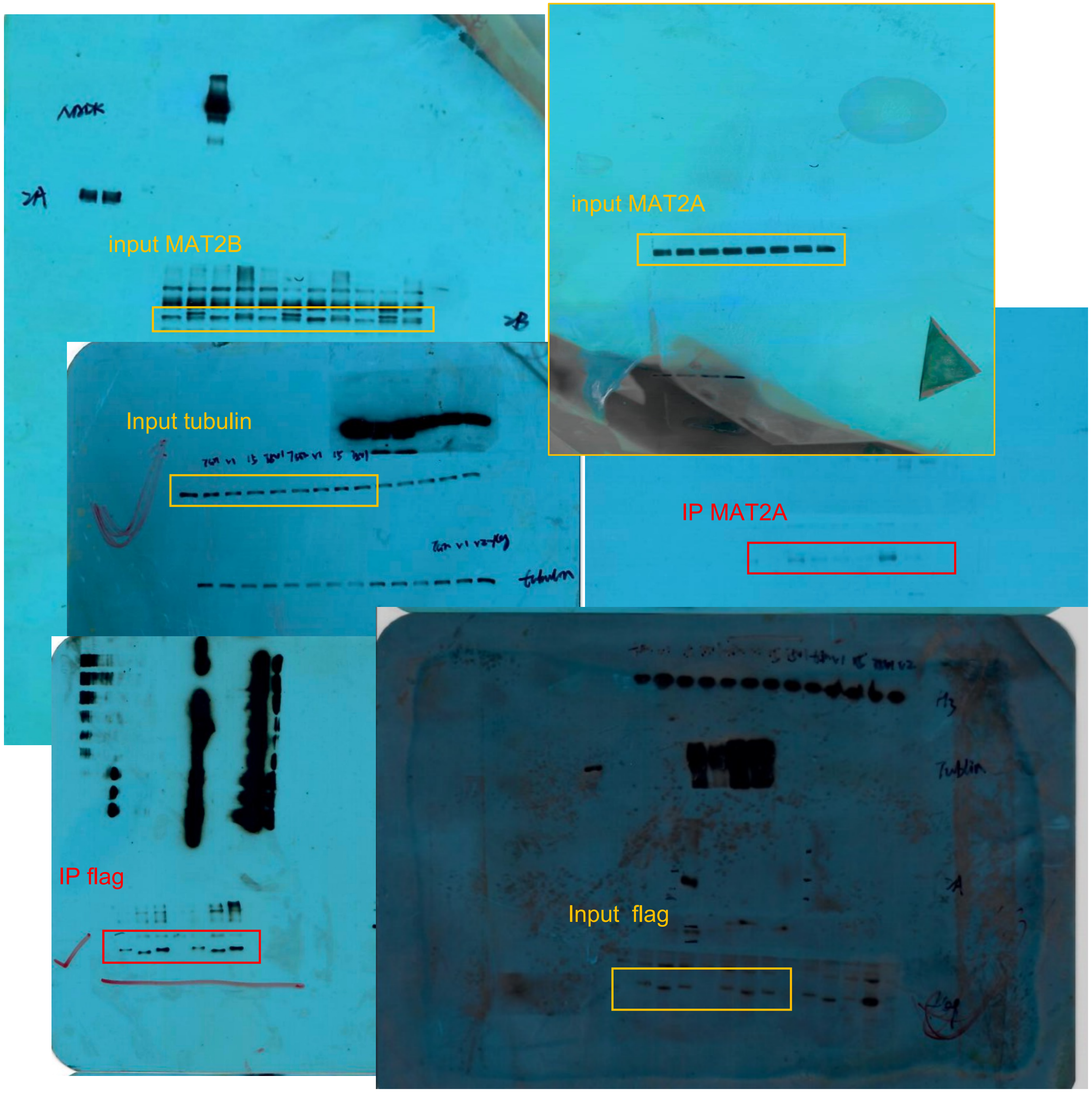
input MAT2B
input MAT2A
Input tubulin
IP MAT2A
IP flag
Input flag

Figure 3

Q

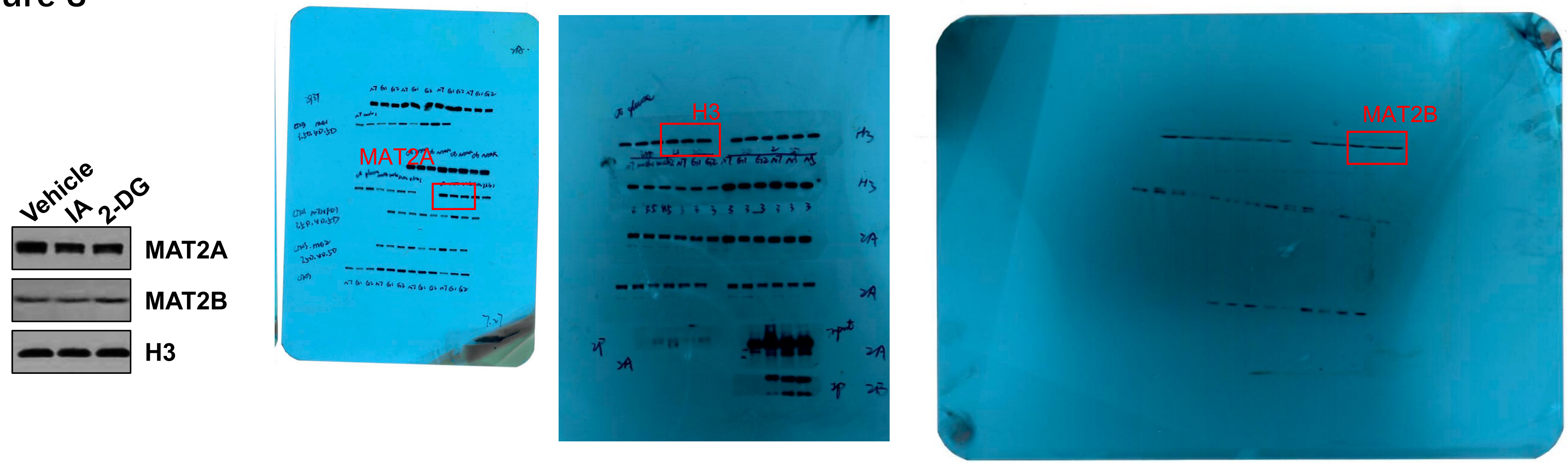
Vehicle
IA
2-DG
MAT2A
MAT2B
H3
MAT2A
H3
MAT2B

R

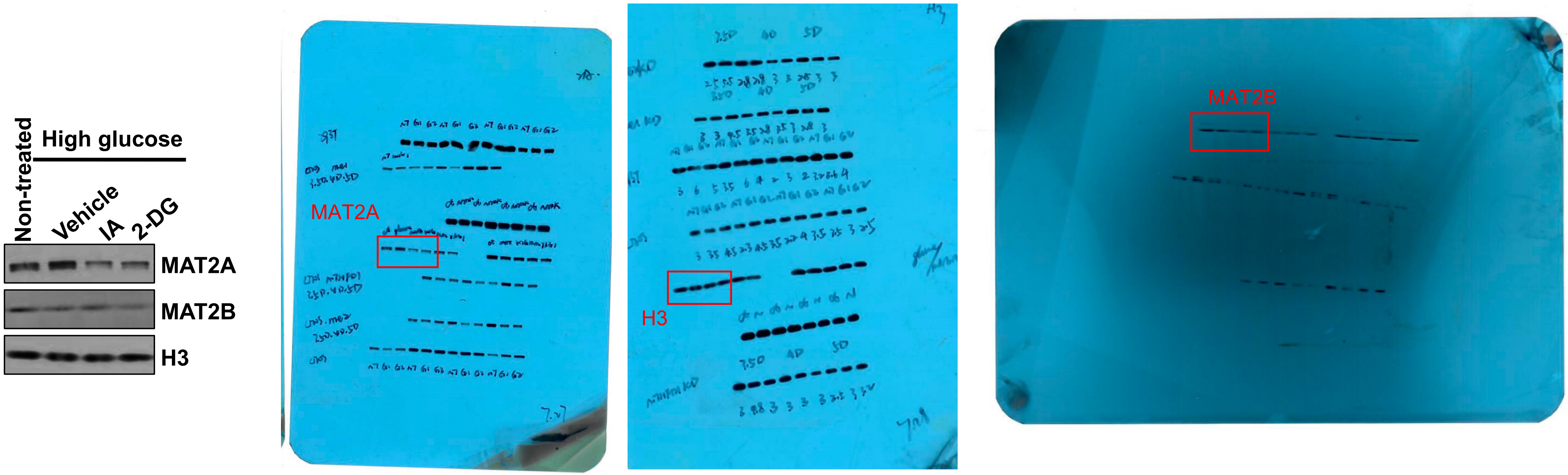
Non-treated
High glucose
Vehicle
IA
2-DG
MAT2A
MAT2B
H3
MAT2A
H3
MAT2B

Figure 3

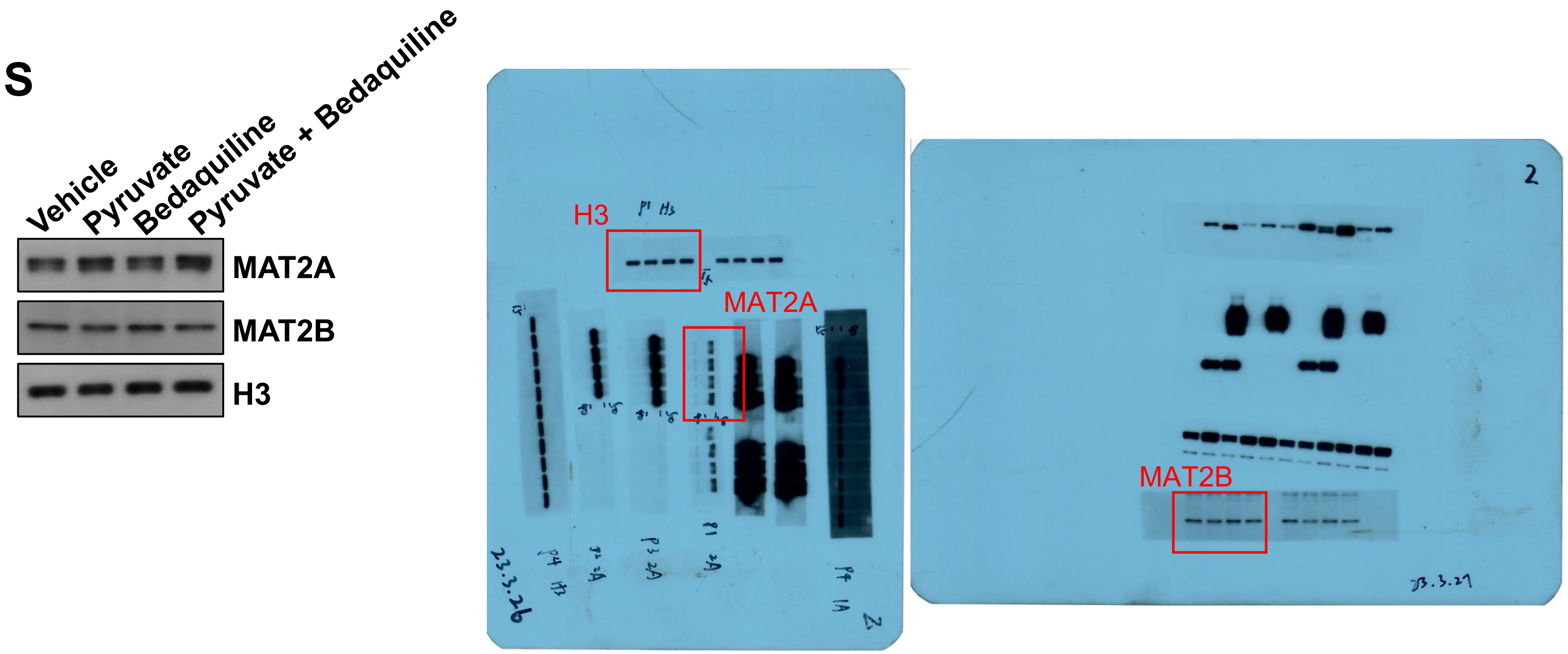
S
Vehicle
Pyruvate
Bedaquiline
Pyruvate + Bedaquiline
MAT2A
MAT2B
H3
H3
MAT2A
MAT2B

Figure S3

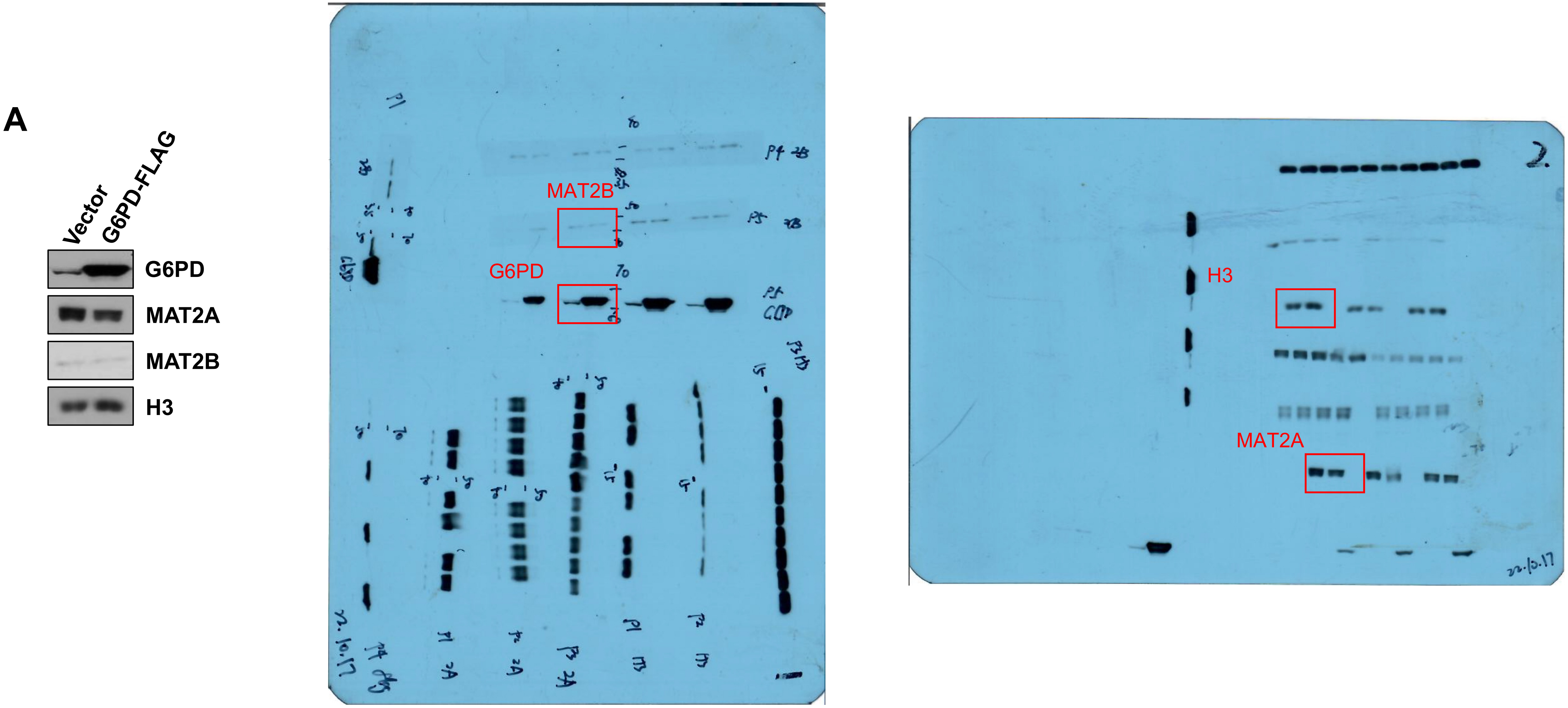

Figure S3

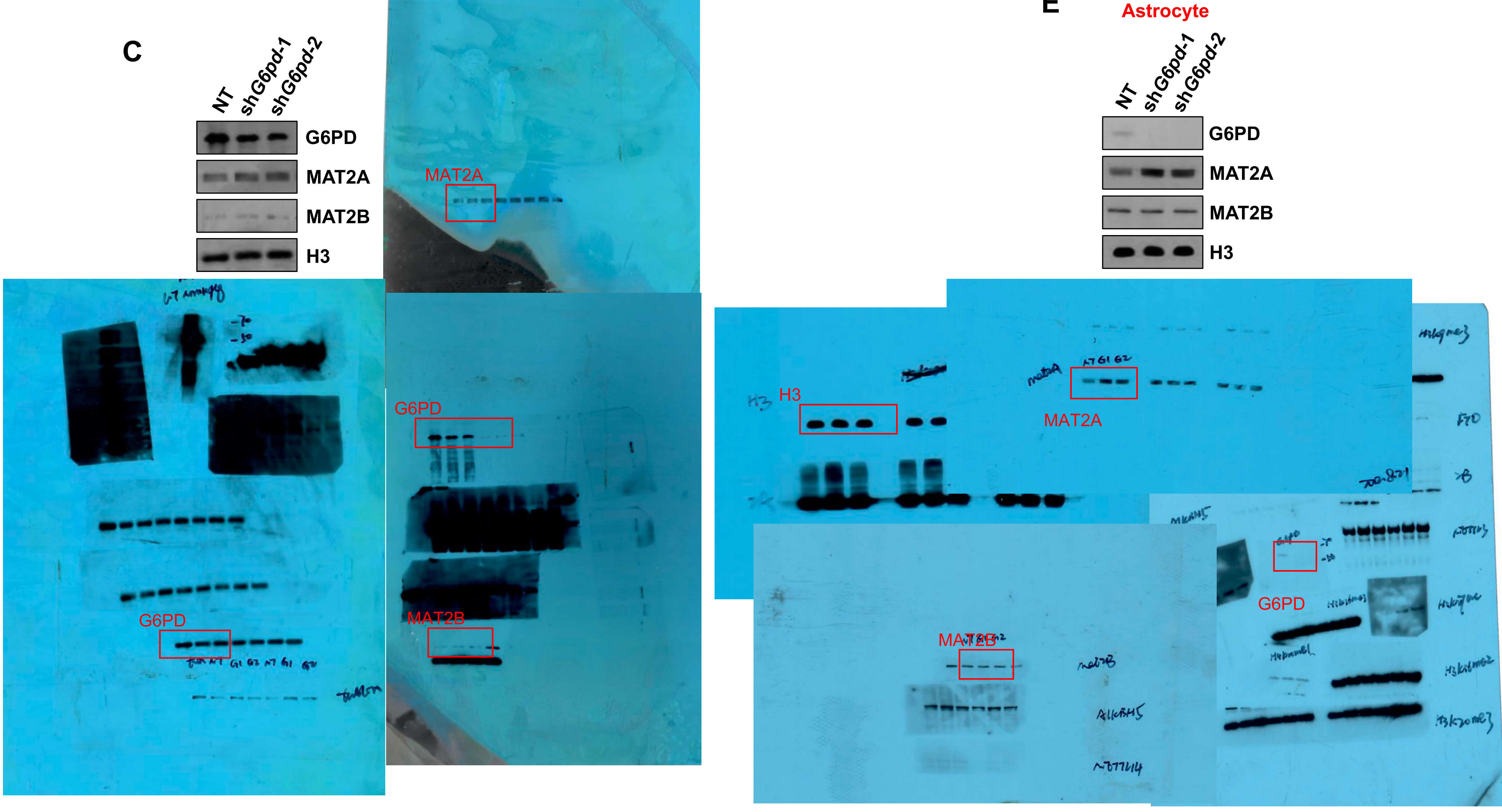
C
NT
shG6pd-1
shG6pd-2
G6PD
MAT2A
MAT2B
H3
MAT2A
G6PD
G6PD
MAT2B
E
Astrocyte
NT
shG6pd-1
shG6pd-2
G6PD
MAT2A
MAT2B
H3
H3
MAT2A
MAT2B
G6PD

Figure S3

G

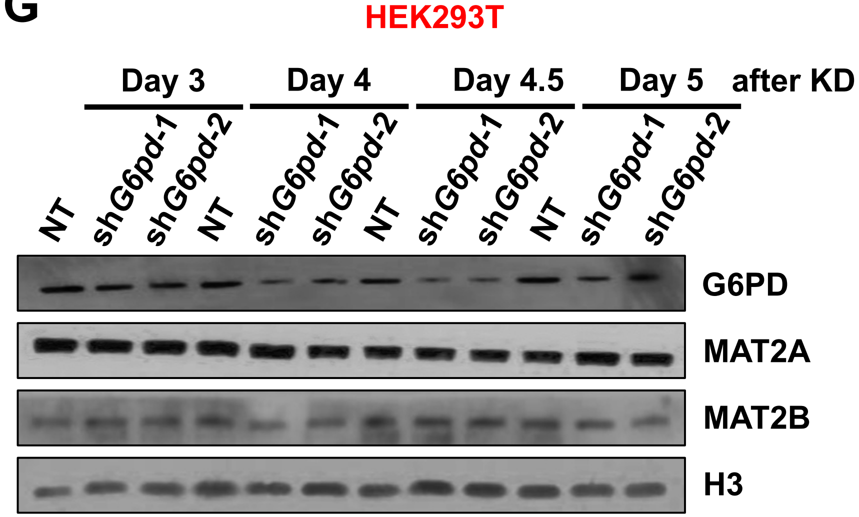
HEK293T
Day 3
Day 4
Day 4.5
Day 5
after KD
NT
shG6pd-1
shG6pd-2
G6PD
MAT2A
MAT2B
H3

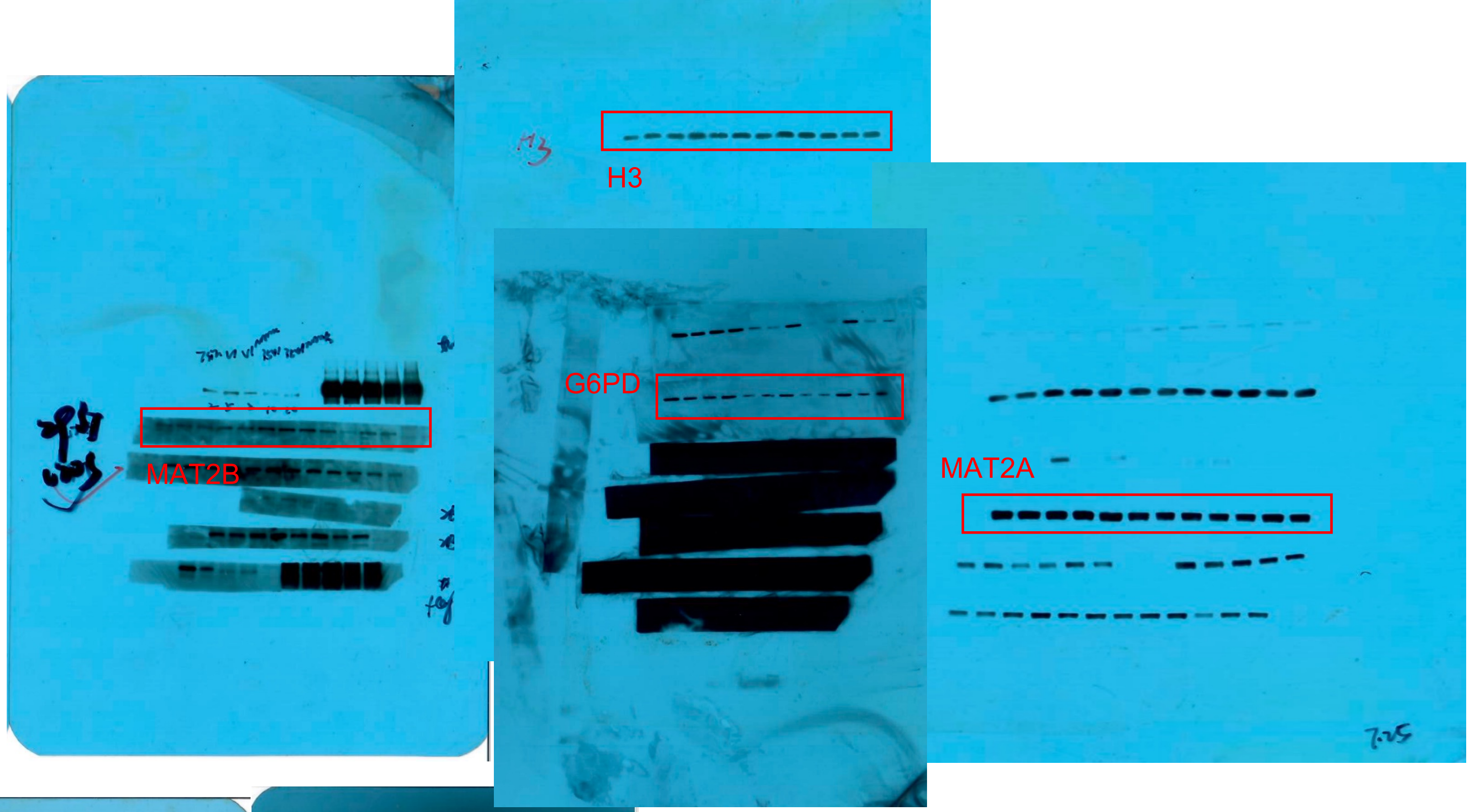
H3
G6PD
MAT2B
MAT2A

H

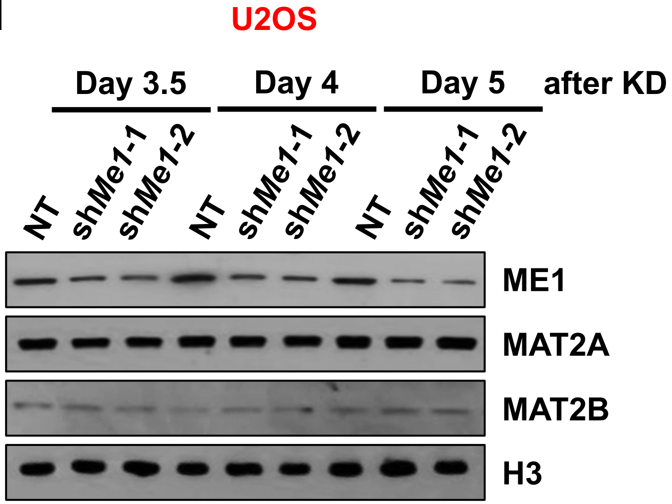
U2OS
Day 3.5
Day 4
Day 5
after KD
NT
shMe1-1
shMe1-2
ME1
MAT2A
MAT2B
H3

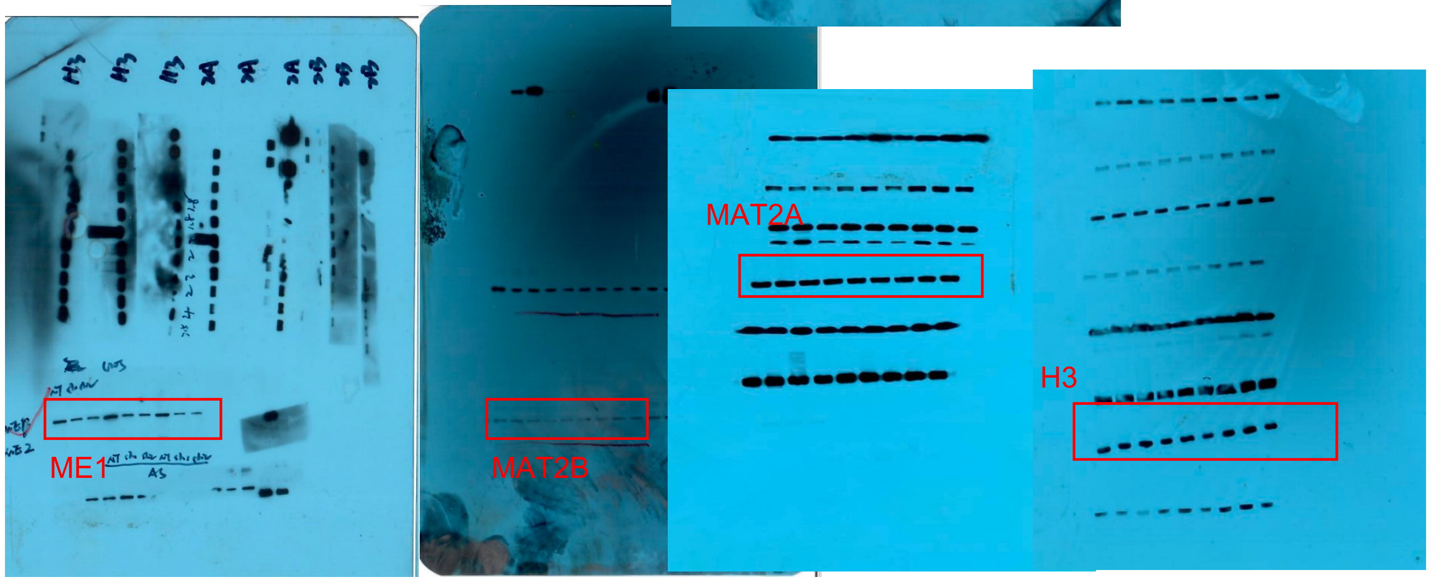
ME1
MAT2B
MAT2A
H3

Figure S3

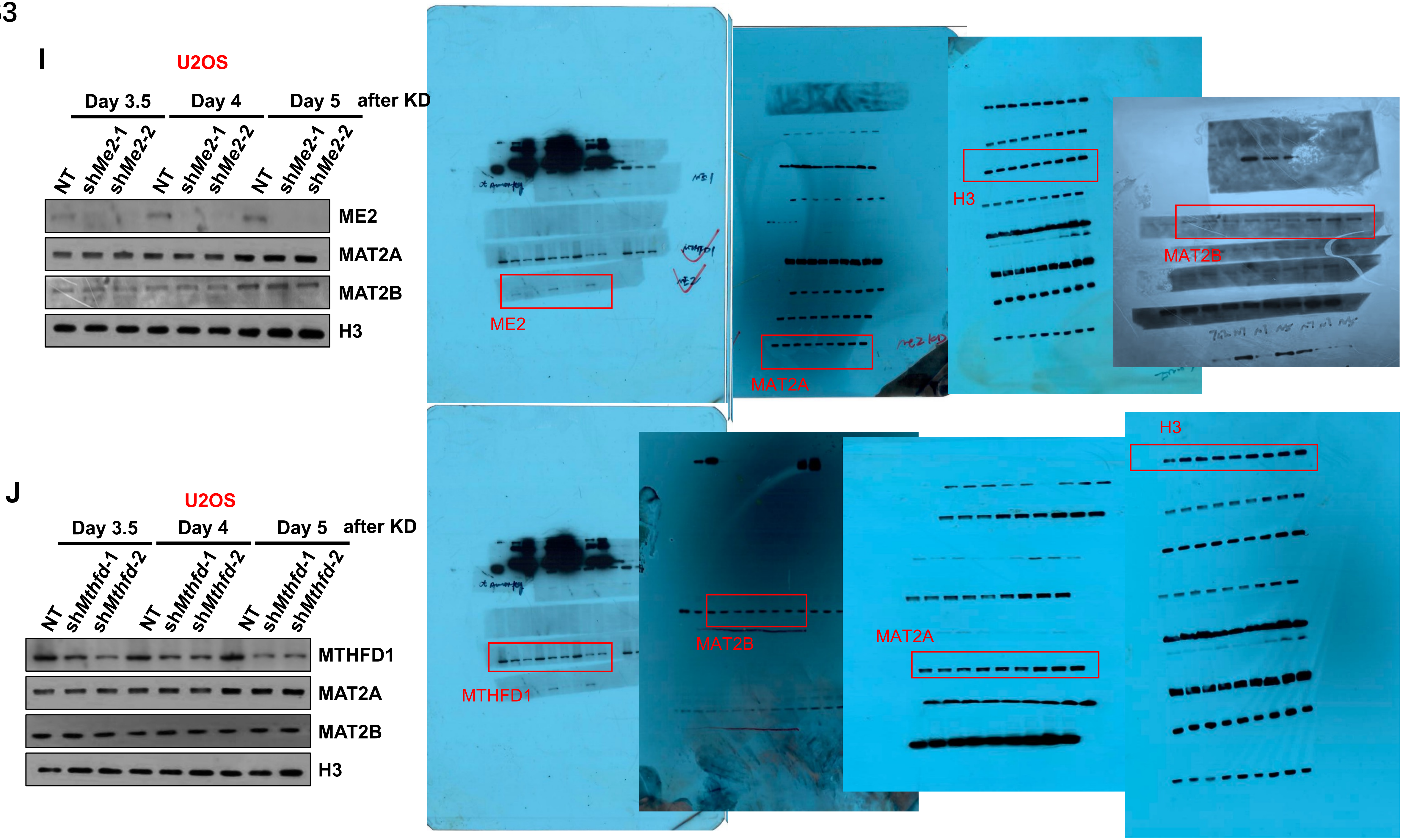
I
U2OS
Day 3.5
Day 4
Day 5
after KD
NT
shMe2-1
shMe2-2
ME2
MAT2A
MAT2B
H3
ME2
MAT2A
H3
MAT2B
J
U2OS
Day 3.5
Day 4
Day 5
after KD
NT
shMthfd-1
shMthfd-2
MTHFD1
MAT2A
MAT2B
H3
MTHFD1
MAT2B
MAT2A
H3

Figure S3

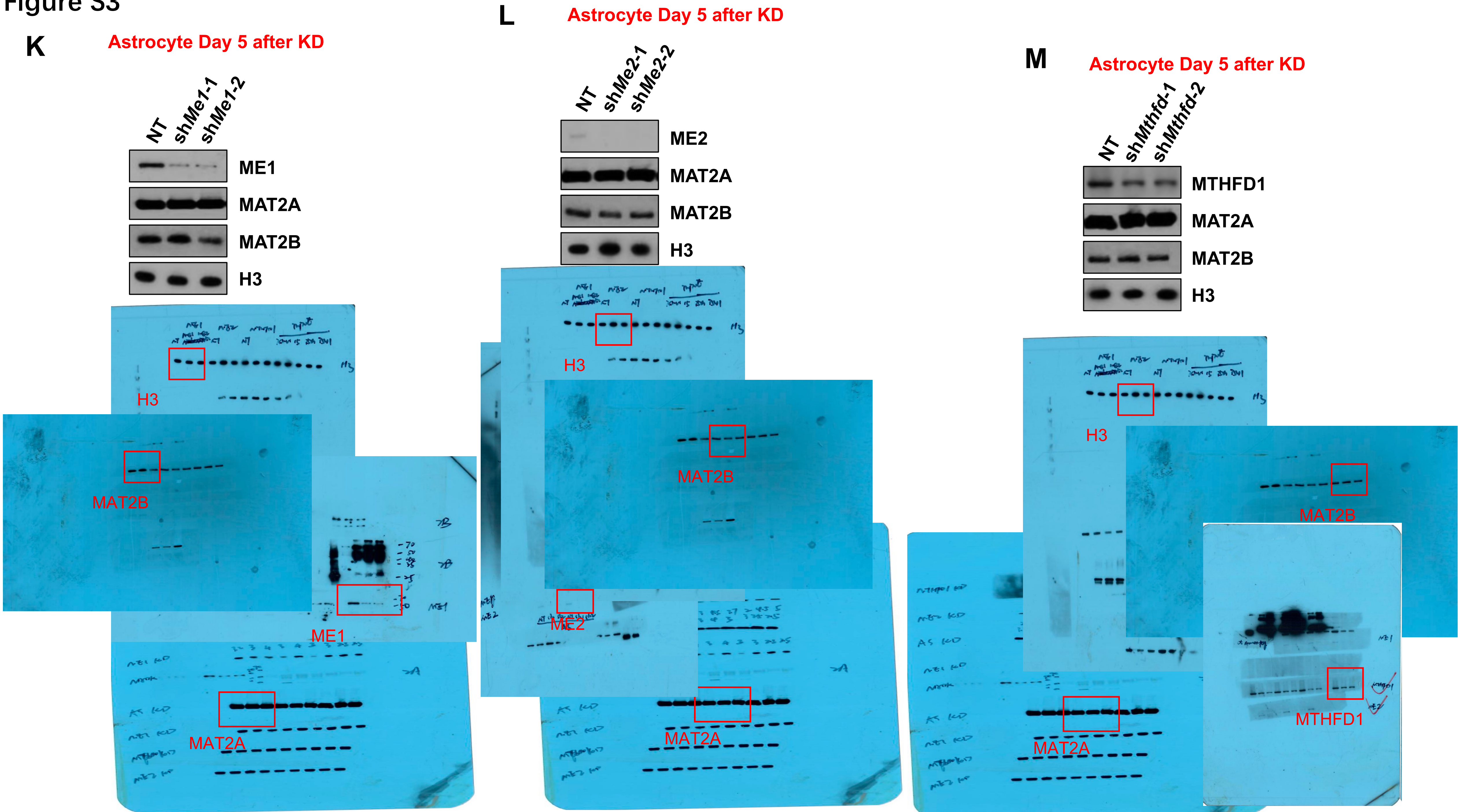
K
Astrocyte Day 5 after KD
NT
shMe1-1
shMe1-2
ME1
MAT2A
MAT2B
H3
H3
MAT2B
ME1
MAT2A
L
Astrocyte Day 5 after KD
NT
shMe2-1
shMe2-2
ME2
MAT2A
MAT2B
H3
H3
MAT2B
ME2
MAT2A
M
Astrocyte Day 5 after KD
NT
shMthfd-1
shMthfd-2
MTHFD1
MAT2A
MAT2B
H3
H3
MAT2B
MTHFD1
MAT2A

Figure S3

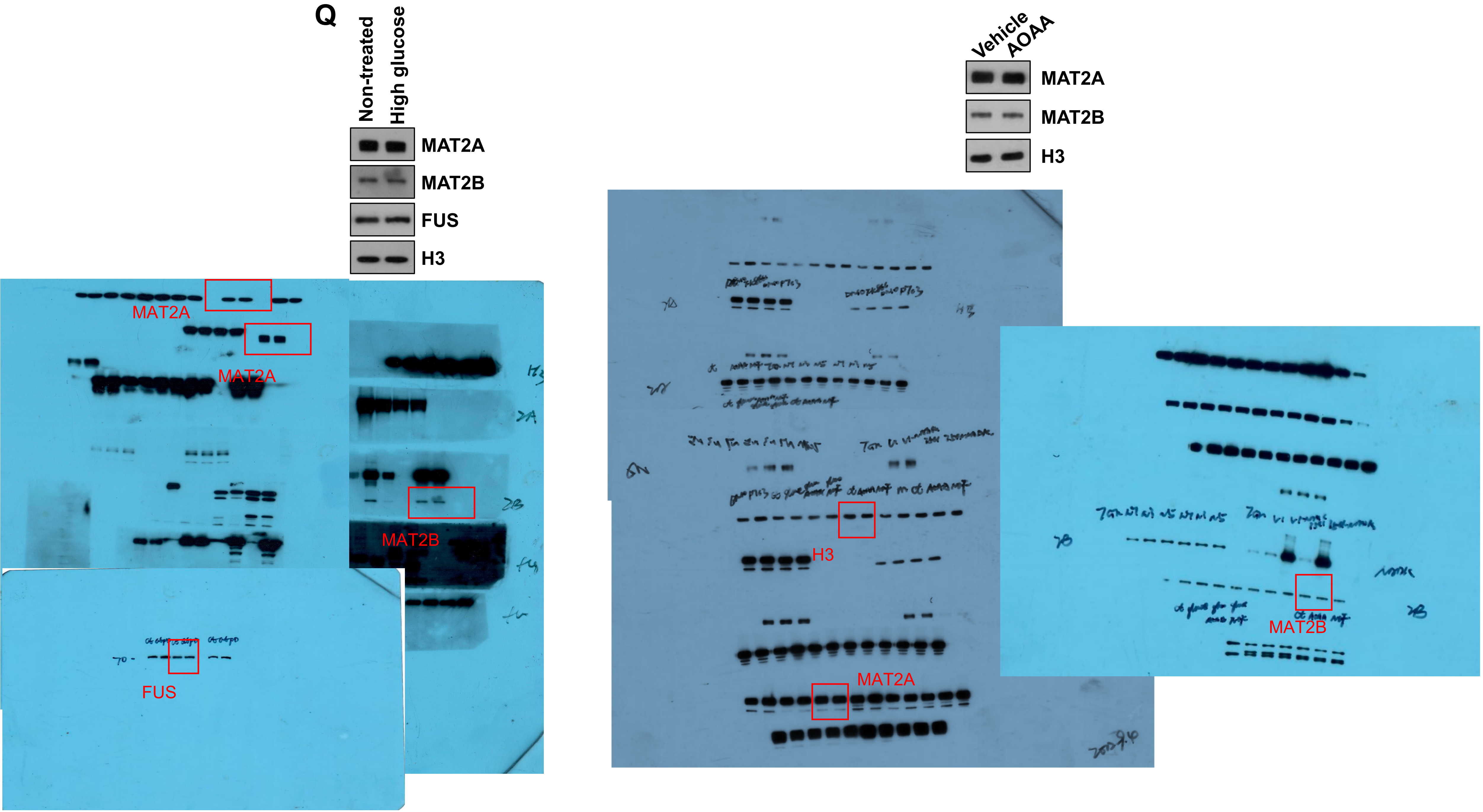
Q
Non-treated
High glucose (3 h)
MAT2A
MAT2B
FUS
H3
MAT2A
MAT2A
MAT2B
FUS
R
Vehicle
AOAA
MAT2A
MAT2B
H3
H3
MAT2A
MAT2B

Figure S3

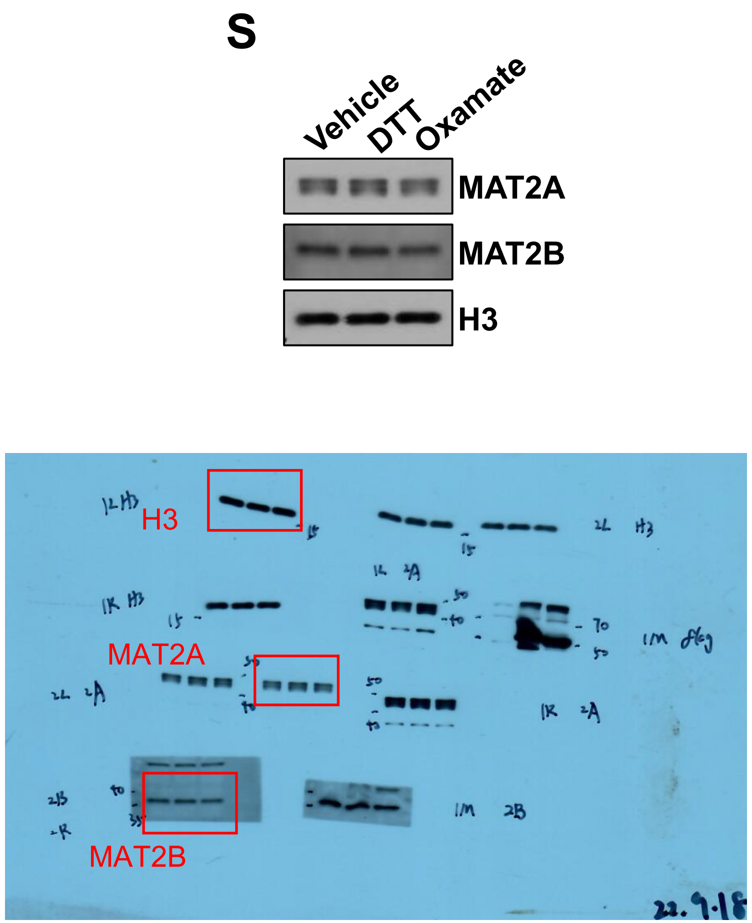
S
Vehicle
DTT
Oxamate
MAT2A
MAT2B
H3
H3
MAT2A
MAT2B

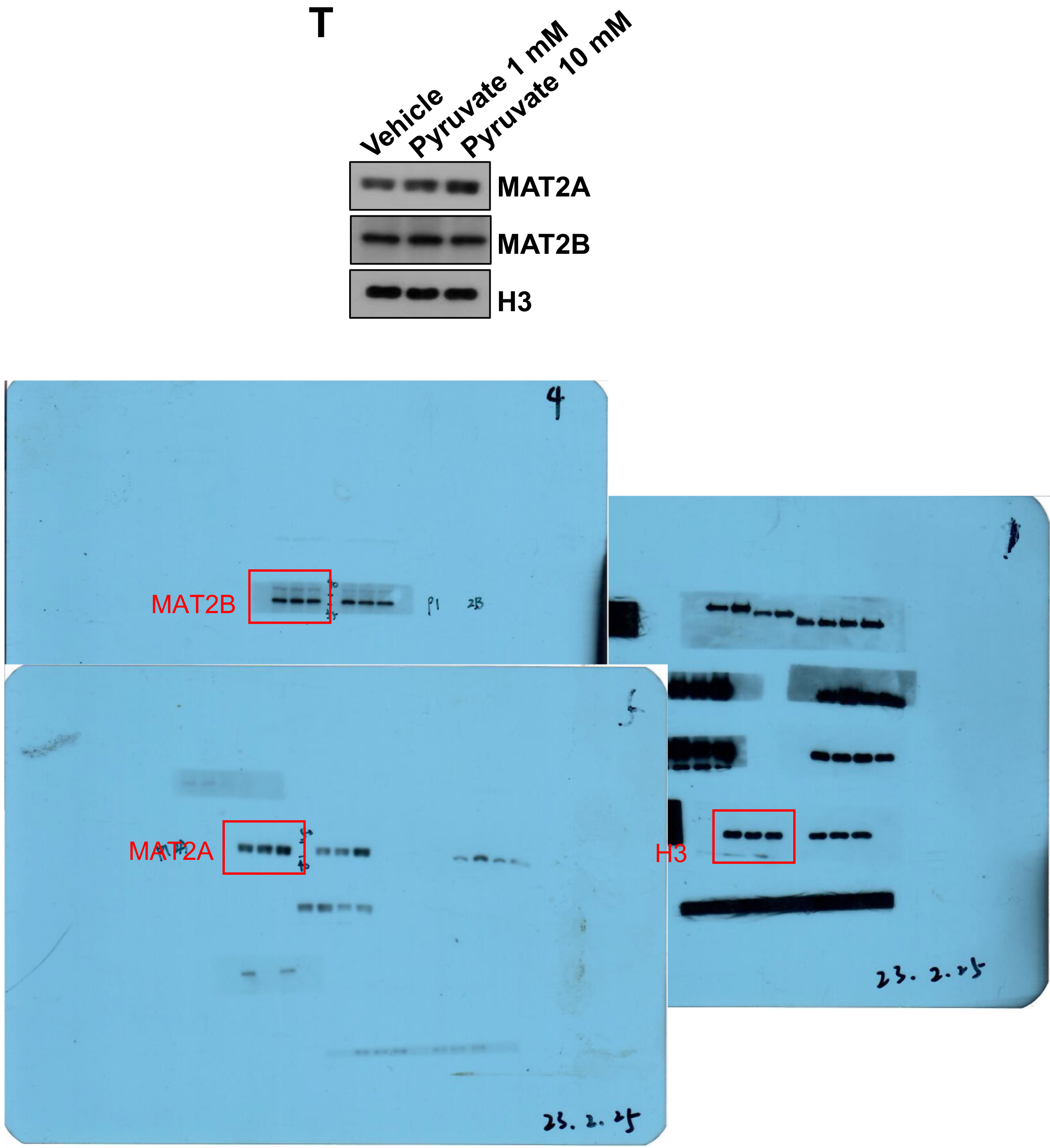
T
Vehicle
Pyruvate 1 mM
Pyruvate 10 mM
MAT2A
MAT2B
H3
MAT2B
MAT2A
H3

Figure S4

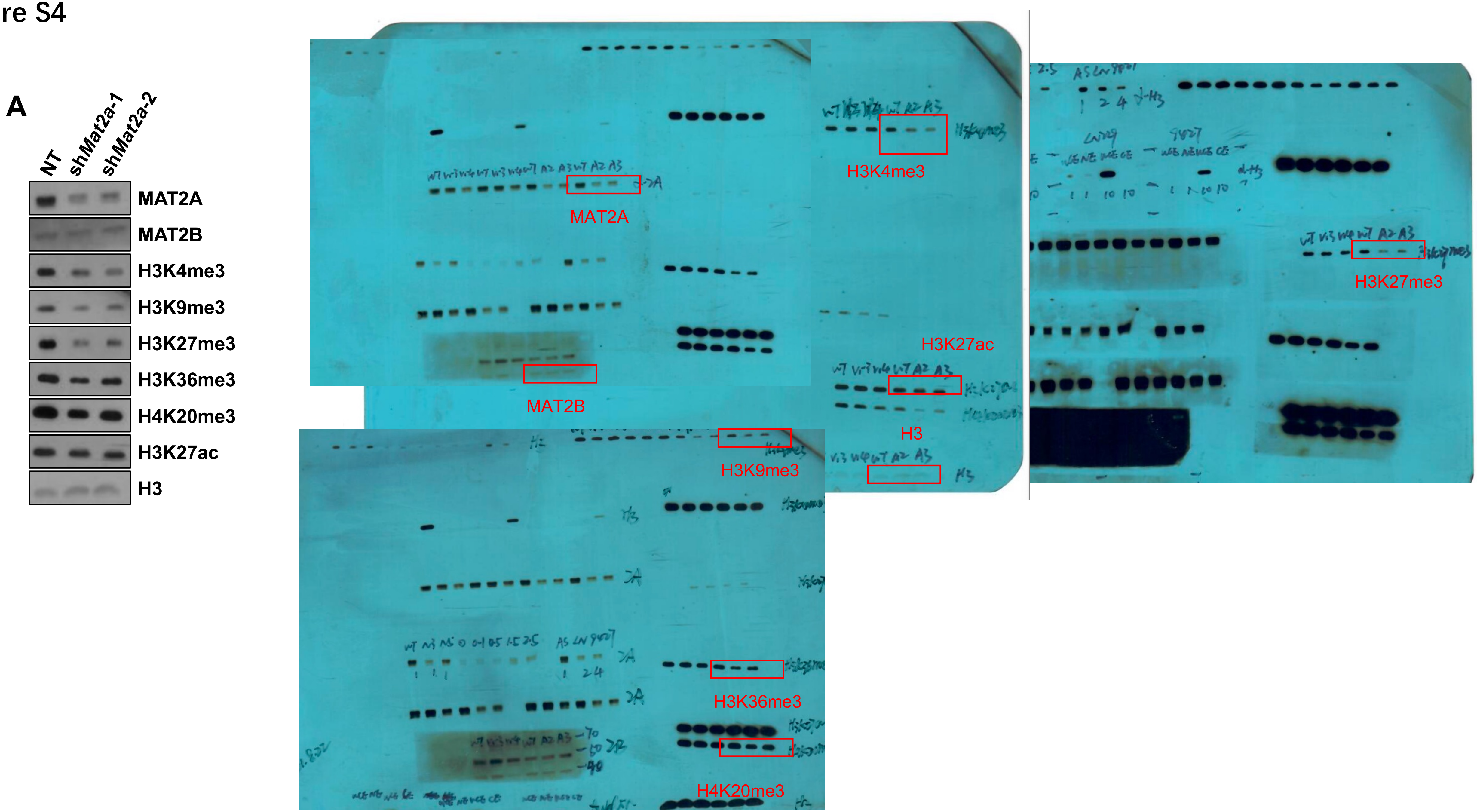
A
NT
shMat2a-1
shMat2a-2
MAT2A
MAT2B
H3K4me3
H3K9me3
H3K27me3
H3K36me3
H4K20me3
H3K27ac
H3
MAT2A
MAT2B
H3K4me3
H3K27ac
H3
H3K9me3
H3K36me3
H4K20me3
H3K27me3

Figure S4

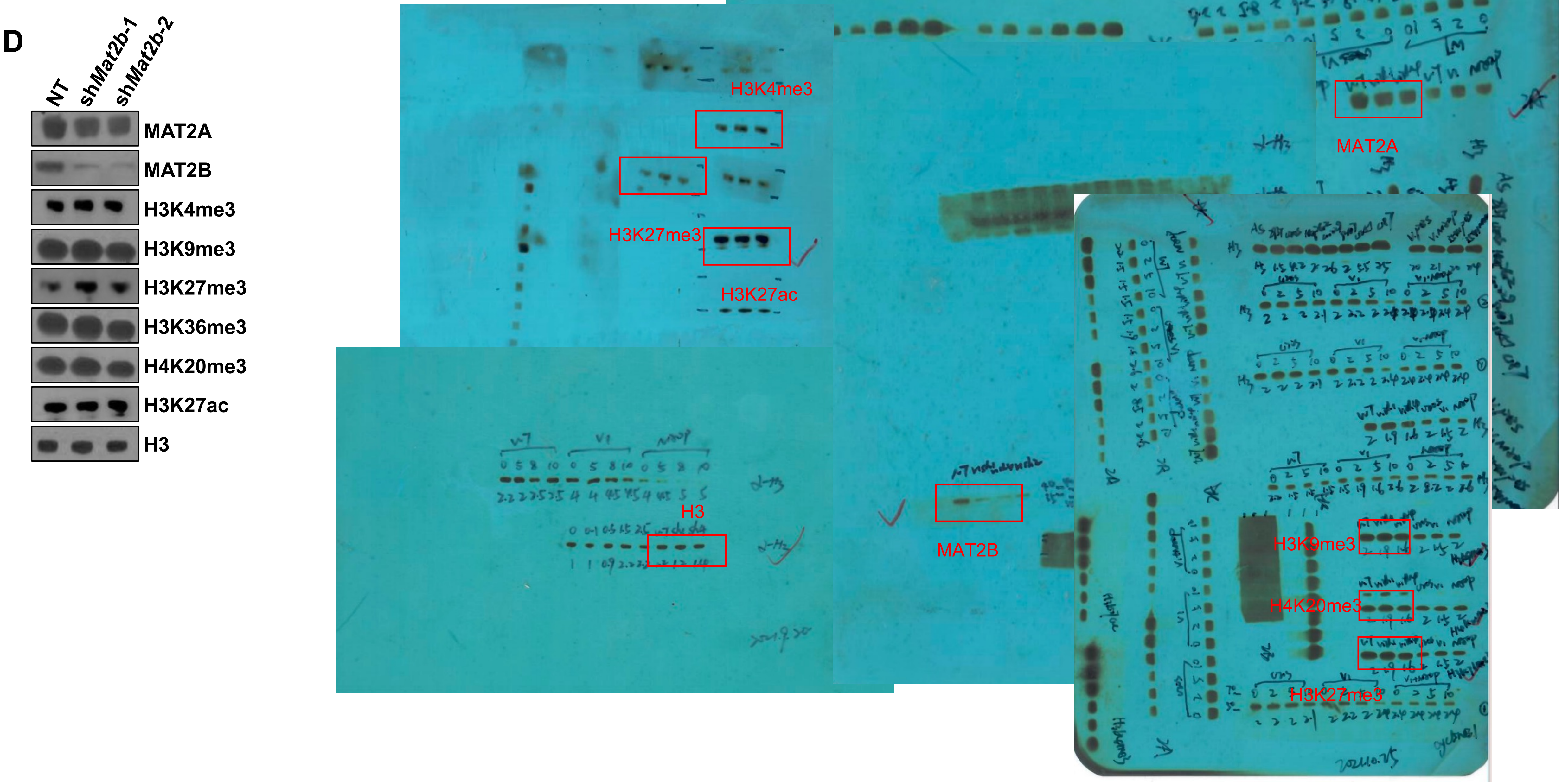
D
NT
shMat2b-1
shMat2b-2
MAT2A
MAT2B
H3K4me3
H3K9me3
H3K27me3
H3K36me3
H4K20me3
H3K27ac
H3
H3K4me3
H3K27me3
H3K27ac
H3
MAT2A
MAT2B
H3K9me3
H4K20me3
H3K27me3

Figure S4

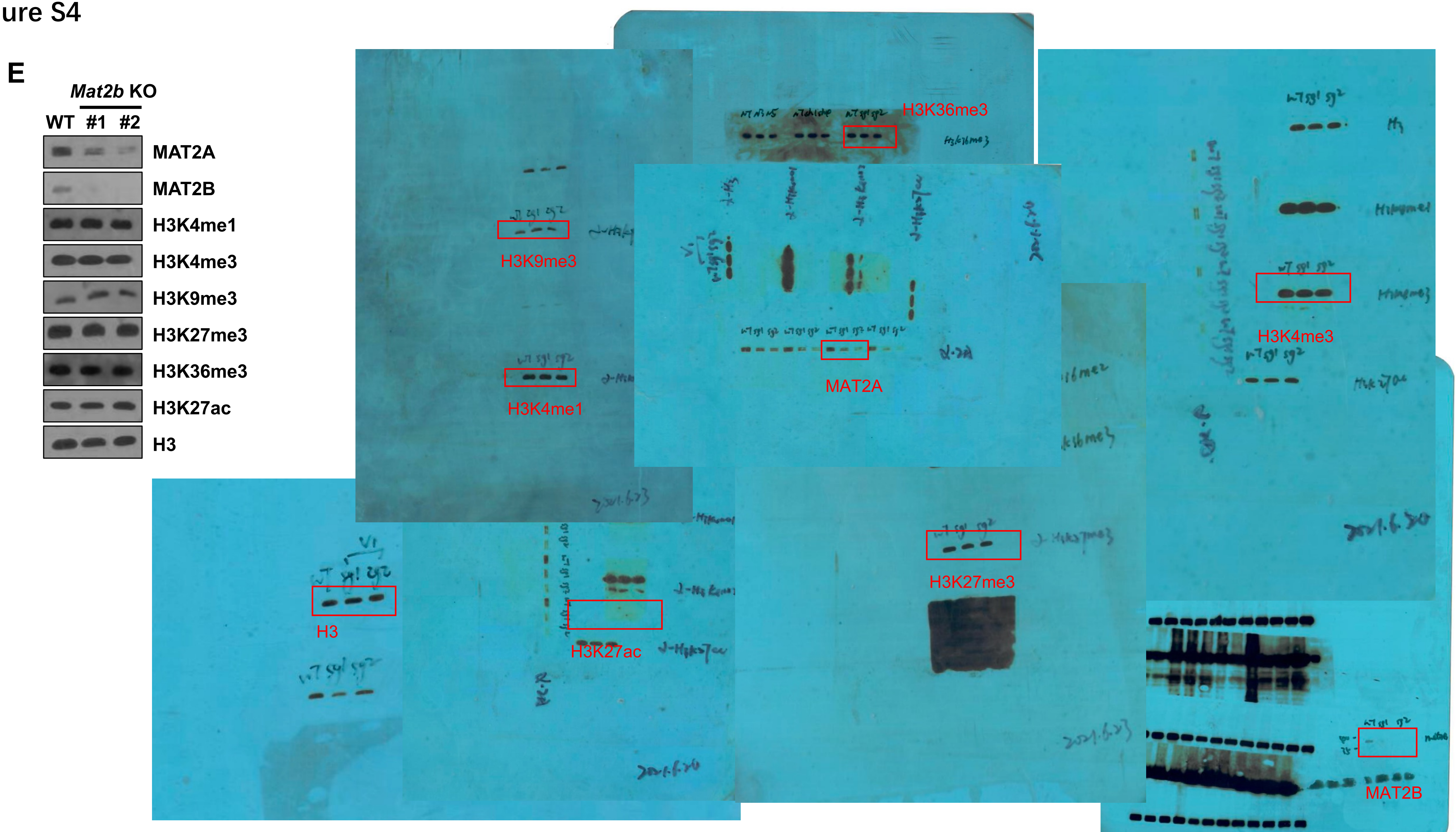
E
Mat2b KO
WT
#1
#2
MAT2A
MAT2B
H3K4me1
H3K4me3
H3K9me3
H3K27me3
H3K36me3
H3K27ac
H3
H3K36me3
H3K9me3
H3K4me1
MAT2A
H3K4me3
H3K27me3
H3
H3K27ac
MAT2B

Figure S4

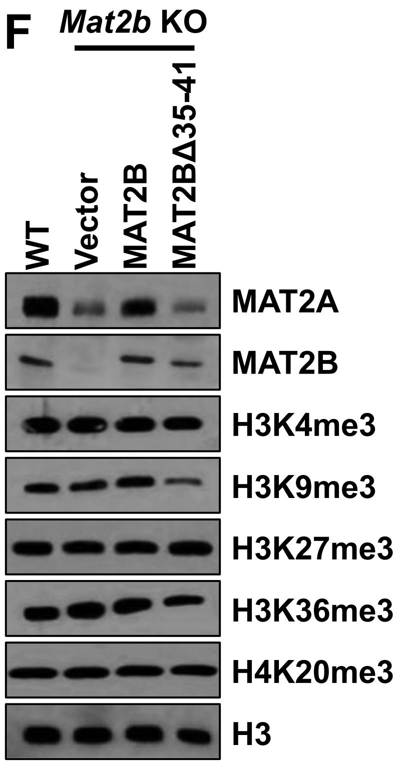
F
Mat2b KO
WT
Vector
MAT2B
MAT2BΔ35-41
MAT2A
MAT2B
H3K4me3
H3K9me3
H3K27me3
H3K36me3
H4K20me3
H3

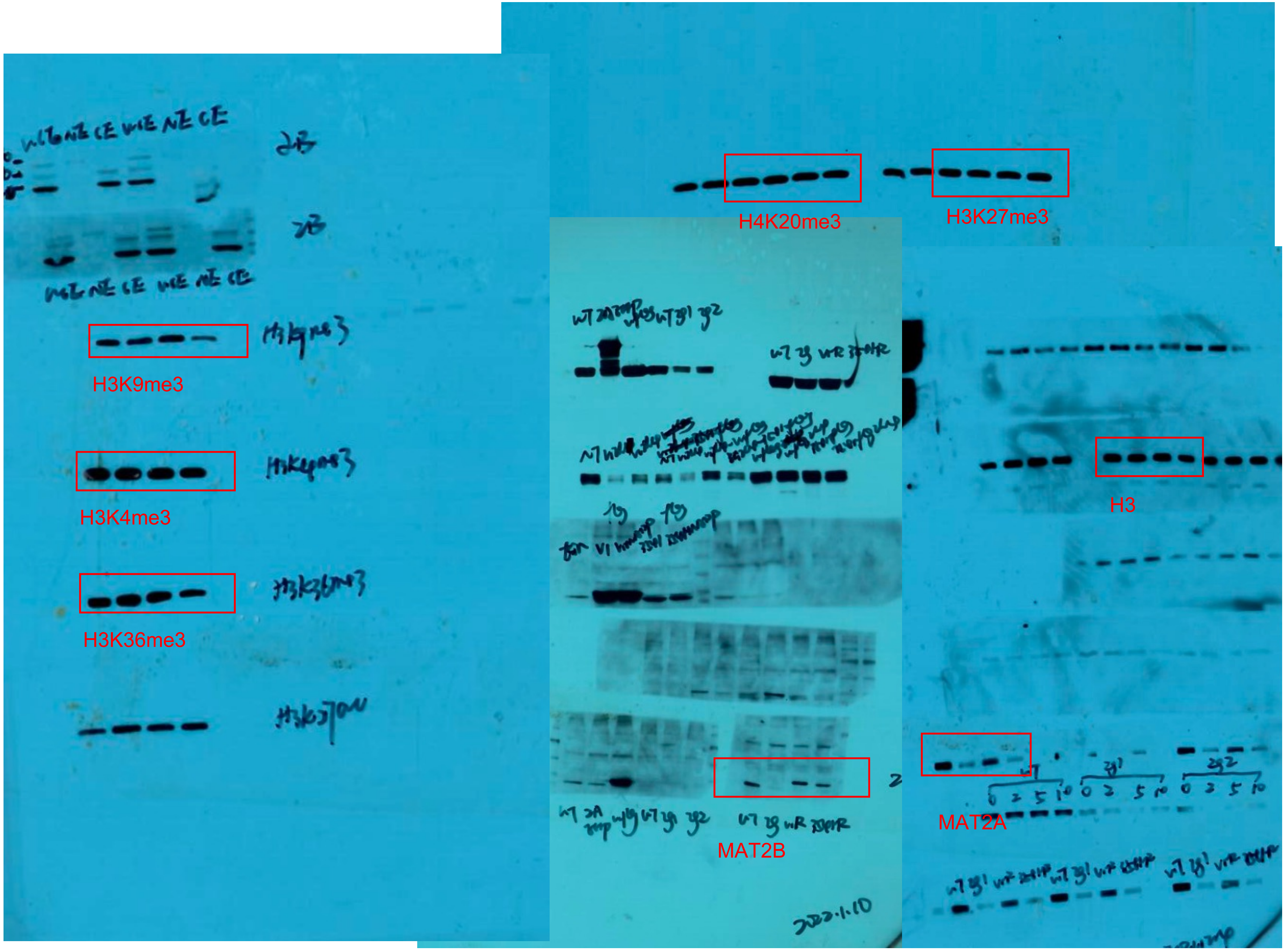
H4K20me3
H3K27me3
H3K9me3
H3K4me3
H3K36me3
H3
MAT2B
MAT2A

Figure S4

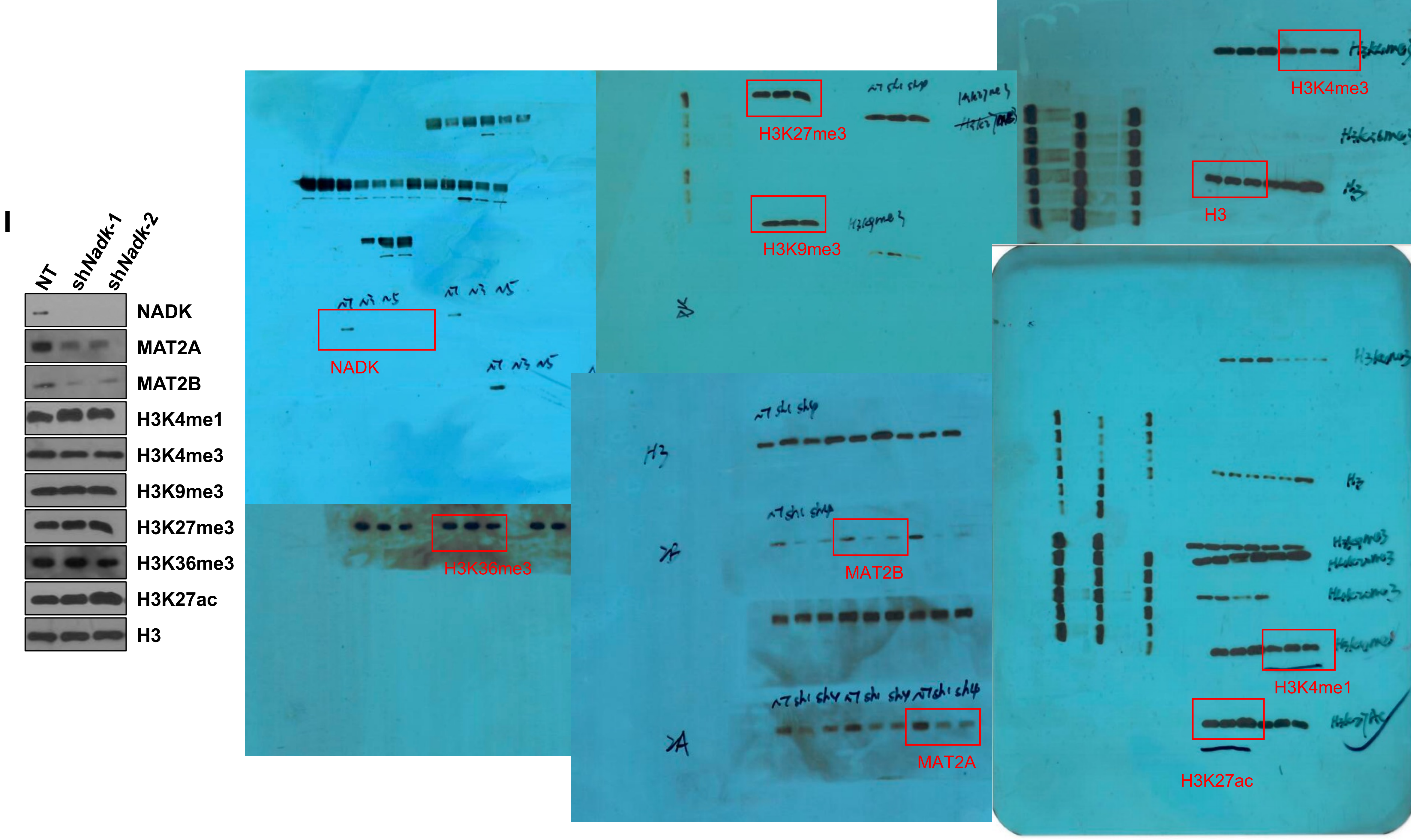
I
NT
shNadk-1
shNadk-2
NADK
MAT2A
MAT2B
H3K4me1
H3K4me3
H3K9me3
H3K27me3
H3K36me3
H3K27ac
H3
NADK
H3K36me3
H3K27me3
H3K9me3
MAT2B
MAT2A
H3K4me3
H3
H3K4me1
H3K27ac

Figure 5

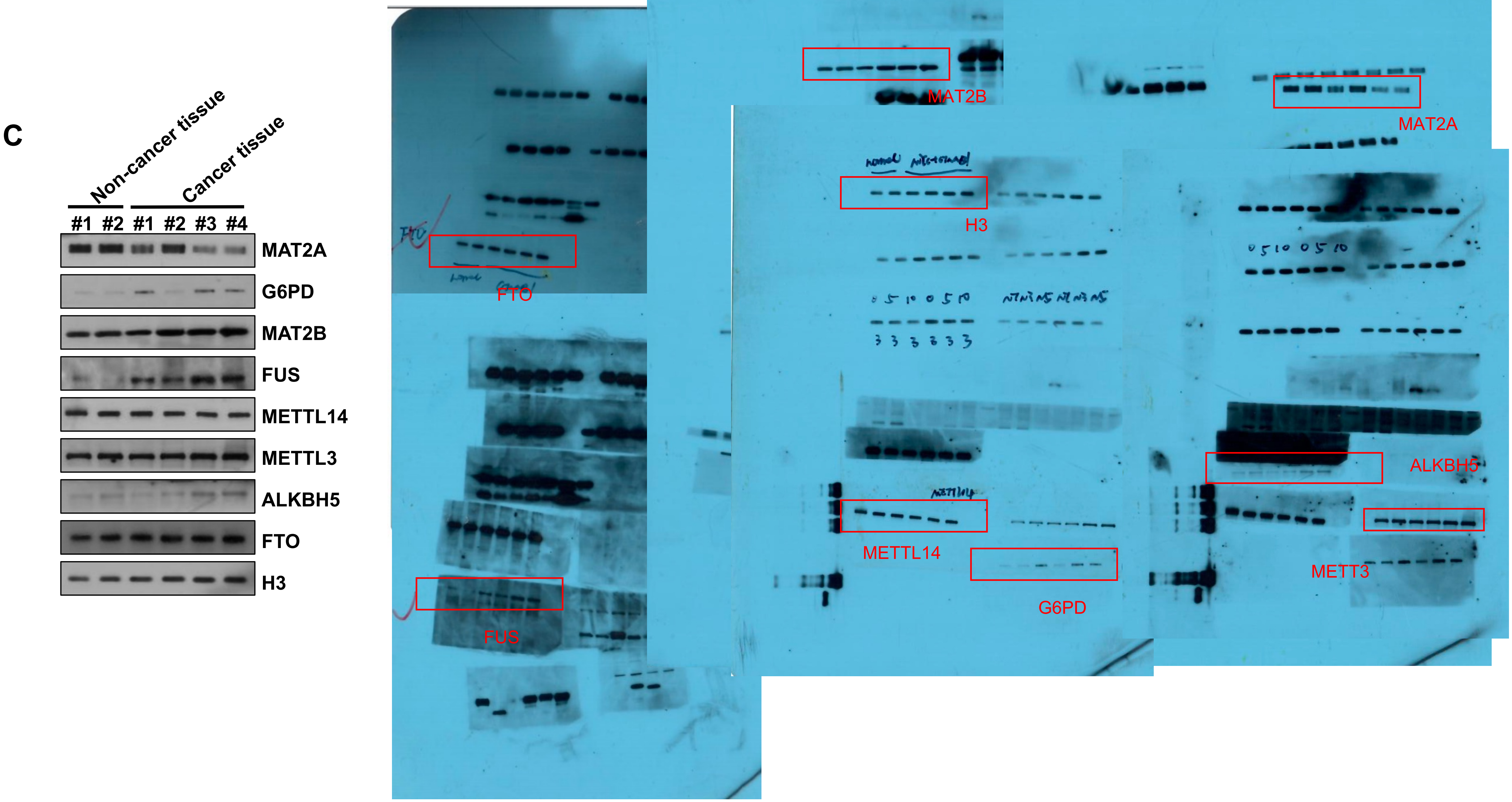
C
Non-cancer tissue
Cancer tissue
#1 #2 #1 #2 #3 #4
MAT2A
G6PD
MAT2B
FUS
METTL14
METTL3
ALKBH5
FTO
H3
FTO
FUS
MAT2B
H3
METTL14
G6PD
MAT2A
ALKBH5
METT3

Figure 5

F

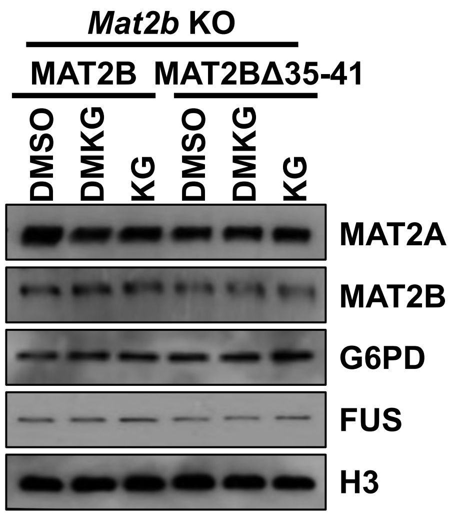

Mat2b KO
MAT2B
MAT2BΔ35-41
DMSO
DMKG
KG
DMSO
DMKG
KG
MAT2A
MAT2B
G6PD
FUS
H3

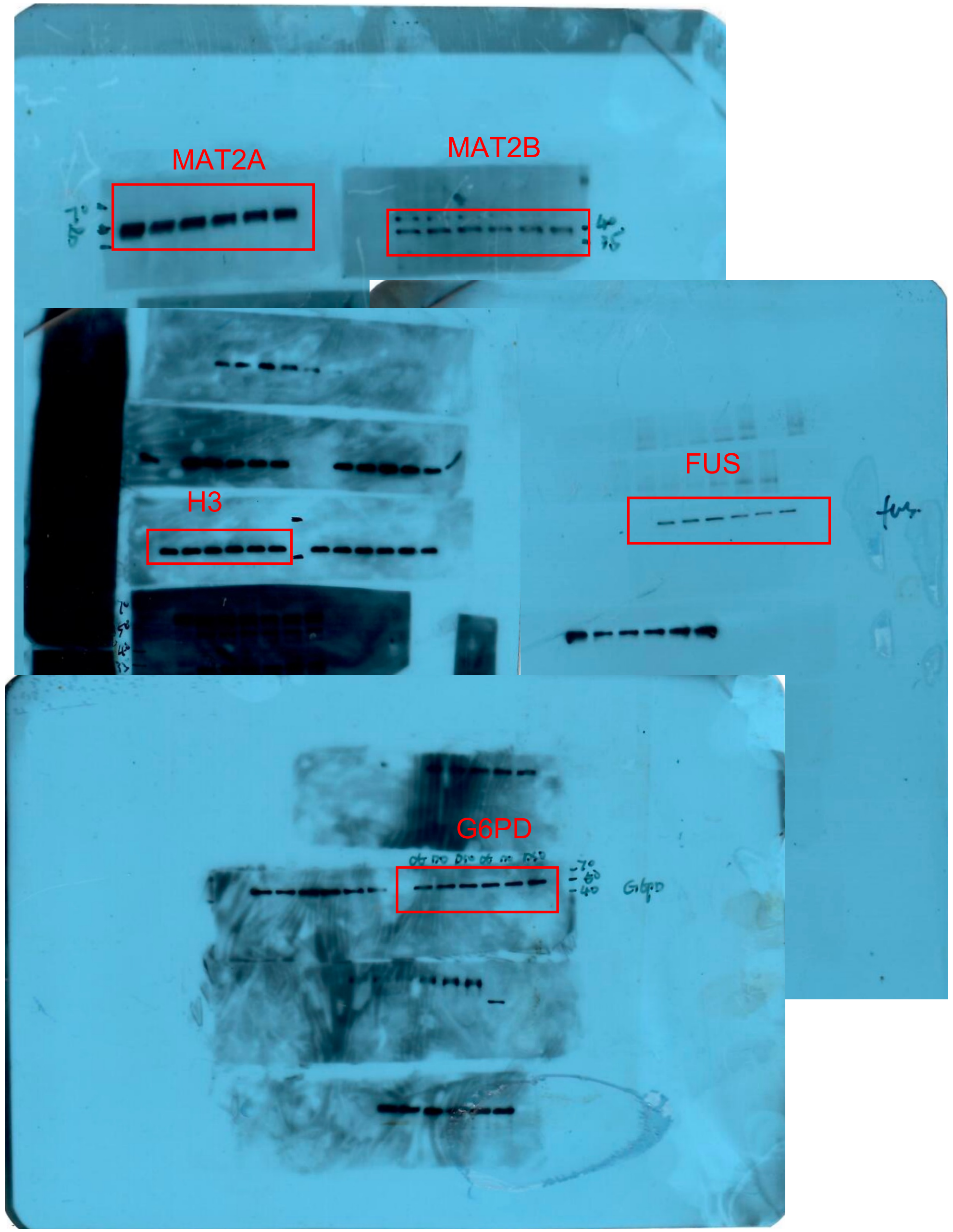

MAT2A
MAT2B
FUS
H3
G6PD

Figure S5

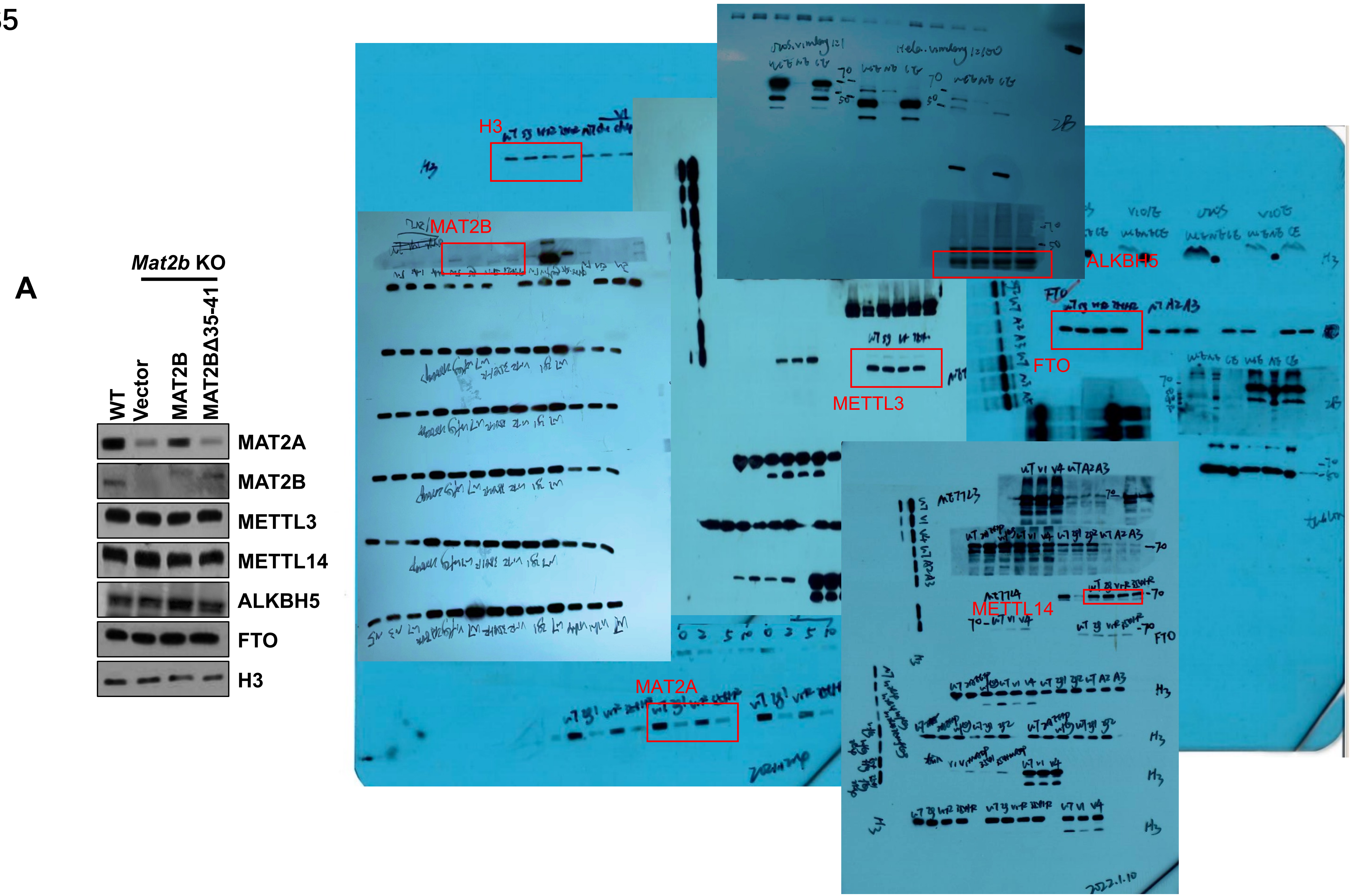
A
Mat2b KO
WT
Vector
MAT2B
MAT2BΔ35-41
MAT2A
MAT2B
METTL3
METTL14
ALKBH5
FTO
H3
H3
MAT2B
ALKBH5
FTO
METTL3
METTL14
MAT2A

Figure 6

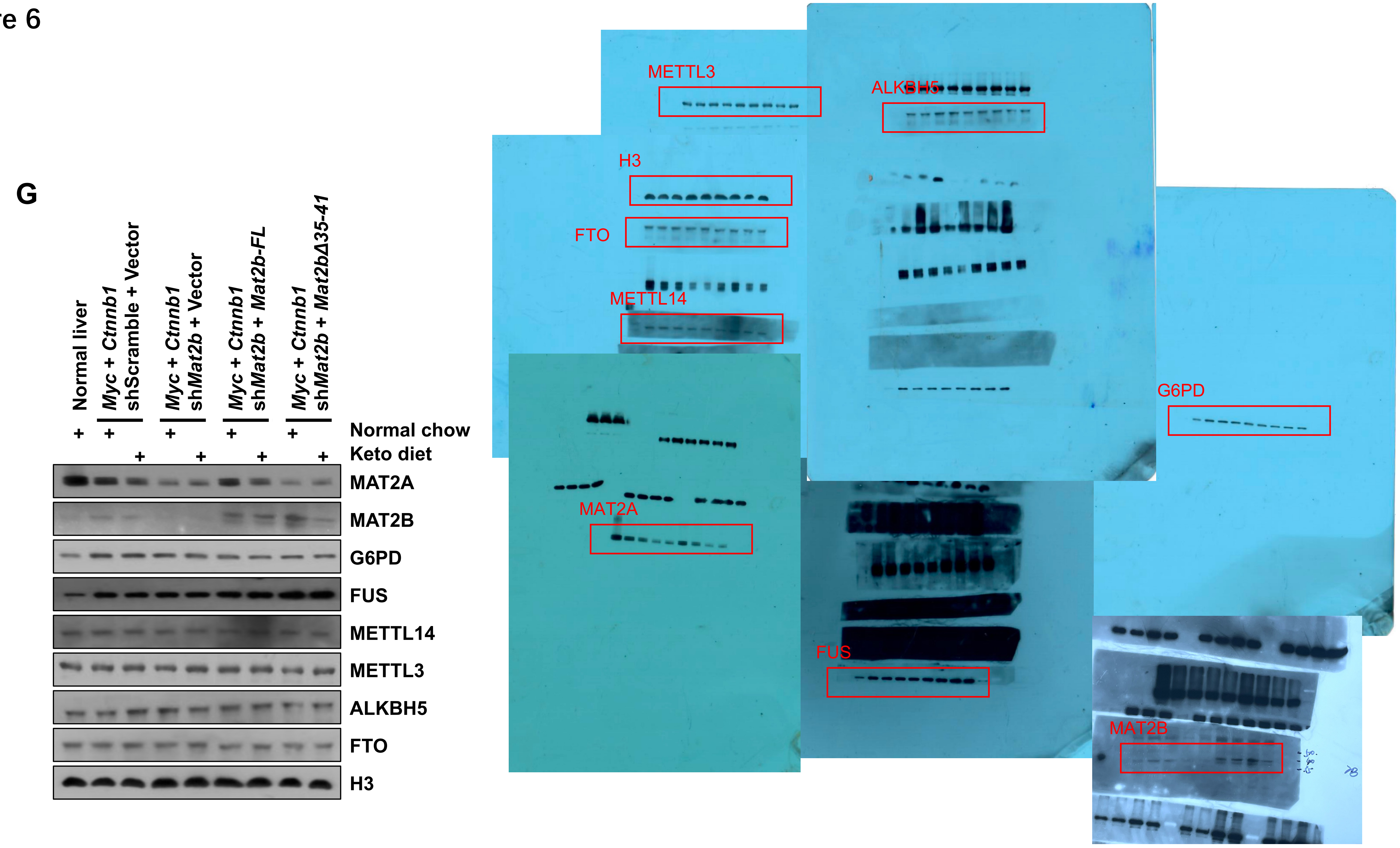
G
Normal liver
Myc + Ctnnb1 shScramble + Vector
Myc + Ctnnb1 shMat2b + Vector
Myc + Ctnnb1 shMat2b + Mat2b-FL
Myc + Ctnnb1 shMat2b + Mat2bΔ35-41
Normal chow
Keto diet
MAT2A
MAT2B
G6PD
FUS
METTL14
METTL3
ALKBH5
FTO
H3
METTL3
ALKBH5
H3
FTO
METTL14
MAT2A
G6PD
FUS
MAT2B

Figure S7

A

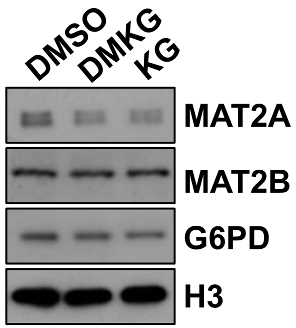

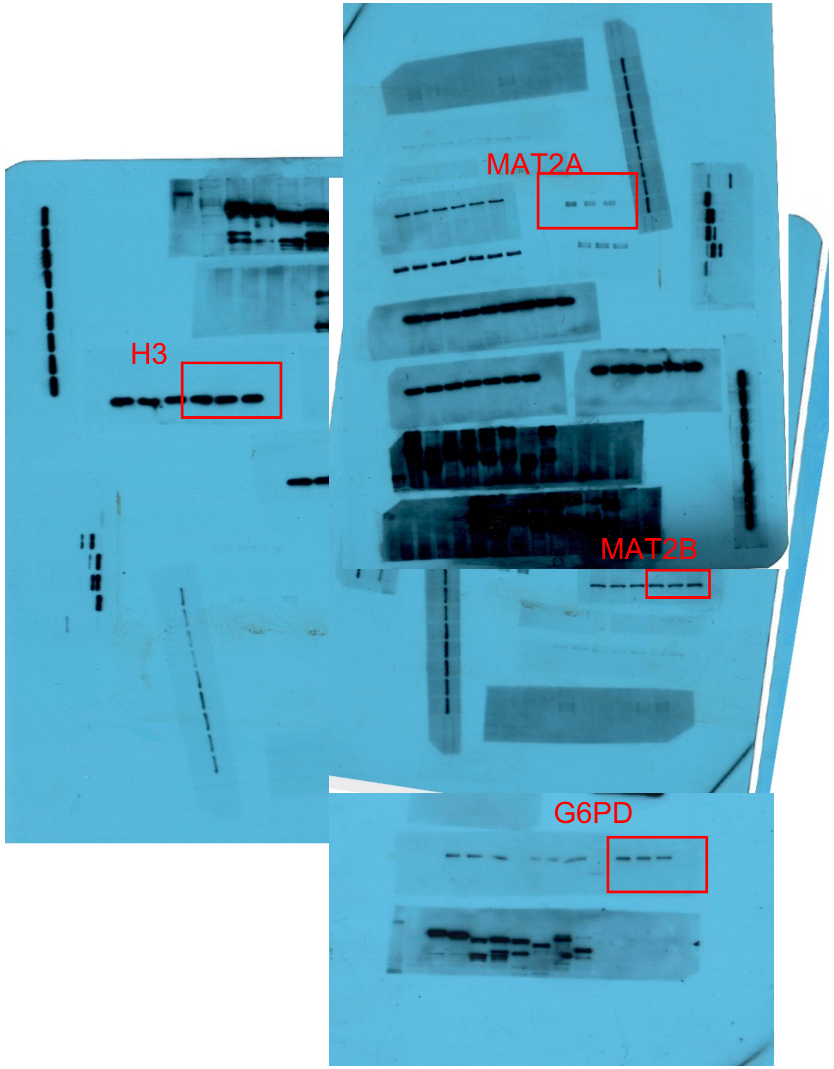

Figure S7

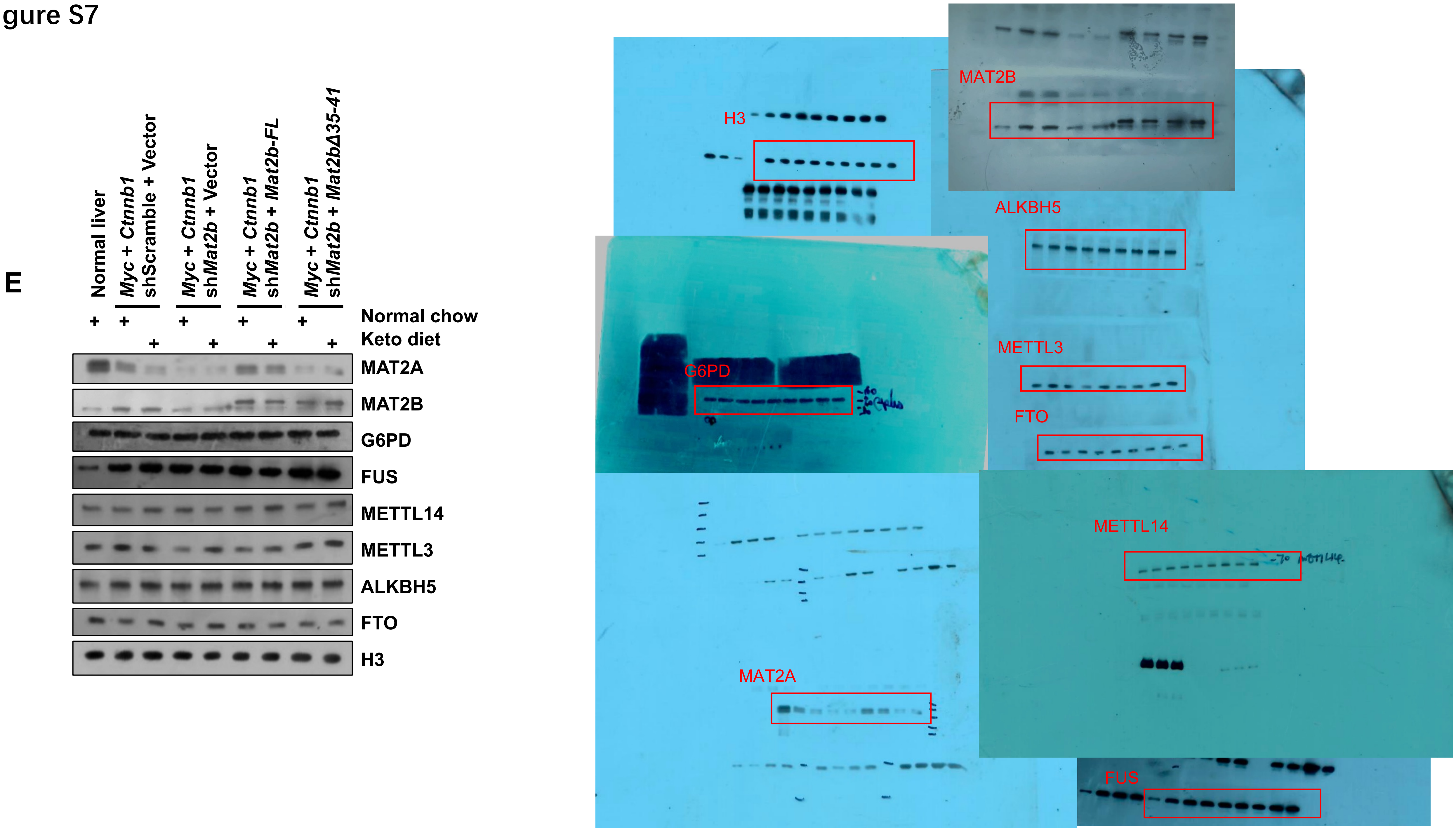
E
Normal liver
Myc + Ctnnb1 shScramble + Vector
Myc + Ctnnb1 shMat2b + Vector
Myc + Ctnnb1 shMat2b + Mat2b-FL
Myc + Ctnnb1 shMat2b + Mat2bΔ35-41
Normal chow
Keto diet
MAT2A
MAT2B
G6PD
FUS
METTL14
METTL3
ALKBH5
FTO
H3
H3
MAT2B
ALKBH5
G6PD
METTL3
FTO
METTL14
MAT2A
FUS

Figure S7

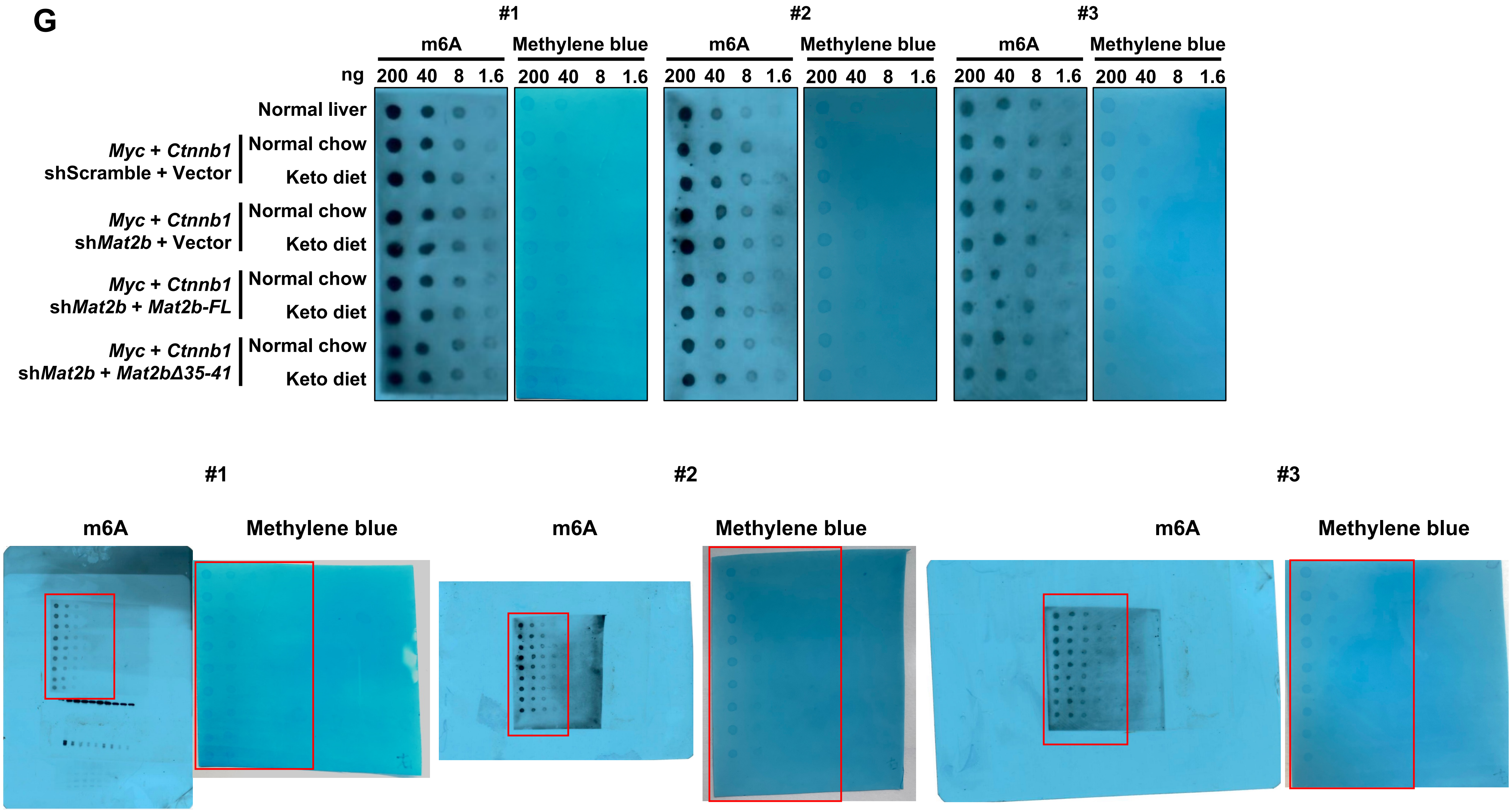
G
#1
#2
#3
m6A
Methylene blue
ng
200
40
8
1.6
Normal liver
Myc + Ctnnb1
shScramble + Vector
Normal chow
Keto diet
Myc + Ctnnb1
shMat2b + Vector
Normal chow
Keto diet
Myc + Ctnnb1
shMat2b + Mat2b-FL
Normal chow
Keto diet
Myc + Ctnnb1
shMat2b + Mat2bΔ35-41
Normal chow
Keto diet
#1
#2
#3
m6A
Methylene blue
m6A
Methylene blue
m6A
Methylene blue
